# Enzymatic synthesis of reactive RNA probes containing squaramate-linked cytidine or adenosine for bioconjugations and cross-linking with lysine-containing peptides and proteins

**DOI:** 10.1038/s42004-024-01399-6

**Published:** 2025-01-02

**Authors:** Ivana Ivancová, Tania Sánchez Quirante, Marek Ondruš, Radek Pohl, Marta Vlková, Eva Žilecká, Evžen Bouřa, Michal Hocek

**Affiliations:** 1 Institute of Organic Chemistry and Biochemistry, Czech Academy of Sciences, Flemingovo nam. 2, CZ-16000 Prague 6, Prague, Czech Republic; 2 Department of Organic Chemistry, Faculty of Science, Charles University, Hlavova 8, CZ-12843 Prague 2, Prague, Czech Republic

**Keywords:** Nucleic acids, RNA, Diversity-oriented synthesis

## Abstract

Protein-RNA interactions play important biological roles and hence reactive RNA probes for cross-linking with proteins are important tools in their identification and study. To this end, we designed and synthesized 5′-*O*-triphosphates bearing a reactive squaramate group attached to position 5 of cytidine or position 7 of 7-deazaadenosine and used them as substrates for polymerase synthesis of modified RNA. In vitro transcription with T7 RNA polymerase or primer extension using TGK polymerase was used for synthesis of squaramate-modified RNA probes which underwent covalent bioconjugations with amine-linked fluorophore and lysine-containing peptides and proteins including several viral RNA polymerases or HIV reverse transcriptase. Inhibition of RNA-depending RNA polymerases from Japanese Encephalitis virus was observed through formation of covalent cross-link which was partially identified by MS/MS analysis. Thus, the squaramate-linked NTP analogs are useful building blocks for the synthesis of reactive RNA probes for bioconjugations with primary amines and cross-linking with lysine residues.

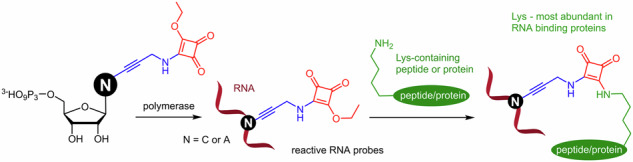

## Abstract (web summary)

RNA-protein interactions are an essential yet underexplored field of research. Here, the authors design and synthesize squaramate-linked cytidine and adenosine analogues and use them as building blocks for the synthesis of reactive RNA probes for bioconjugations with primary amines and cross-linking with lysine-containing peptides and proteins.

## Introduction

**Fig. 1:**
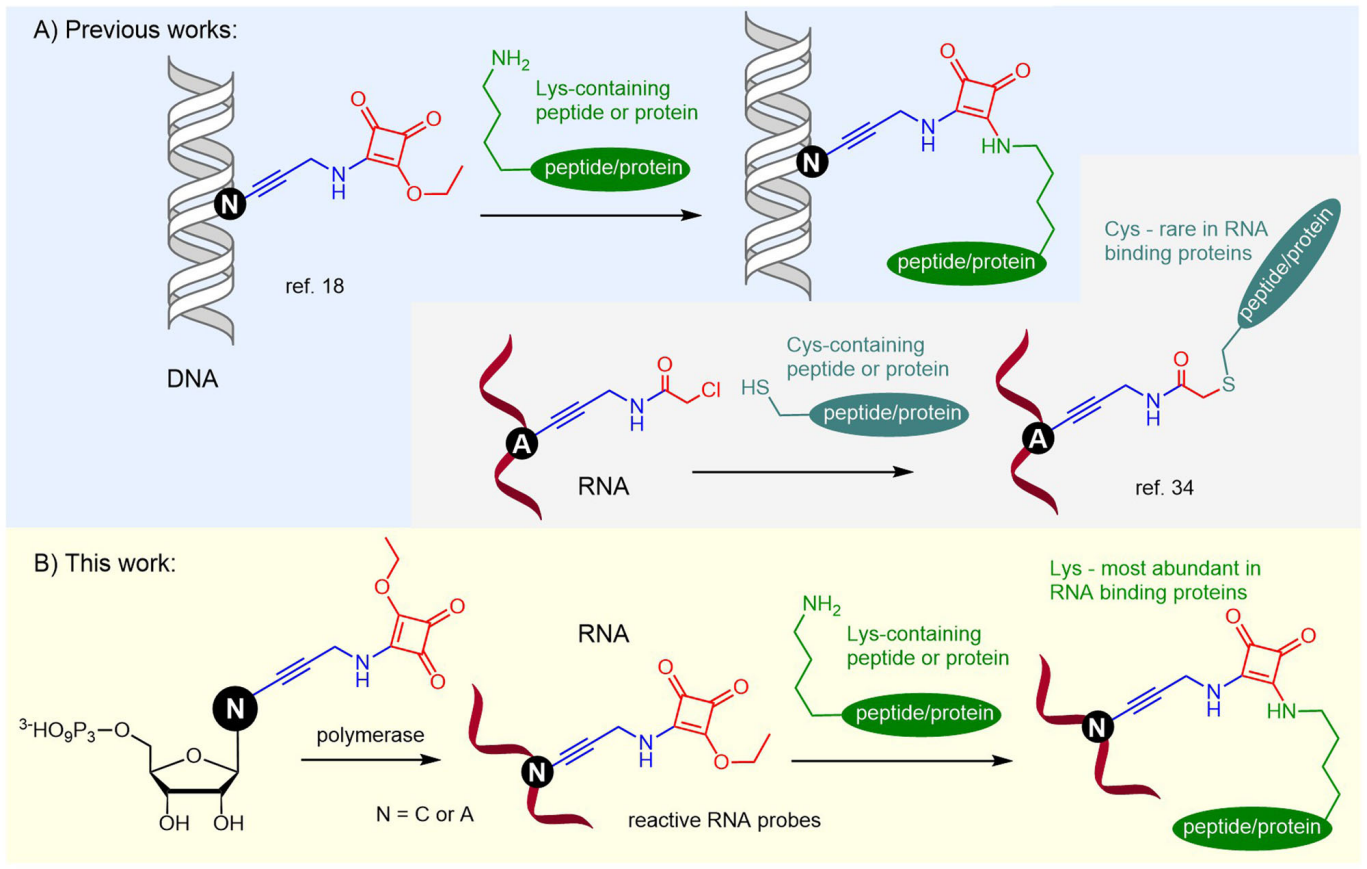
Overview of related reactive DNA and RNA probes. **A** Previous related works on reactive DNA and RNA probes and (**B**) conceptualization of this study: polymerase synthesis of squaramate-containing reactive RNA probes and their cross-linking with Lys-containing peptides or proteins.

RNA-protein interactions are a very important yet underexplored field of research^1^. One of the most efficient ways how to study them is the use of covalent cross-linking of RNA with interacting proteins^2–4^. So far, the common methods include photochemical cross-linking^5–8^ or the usage of external chemical cross-linking agents^9–11^. These methods are often inefficient, non-specific, and toxic for use in live cells. Therefore, there is a need for the introduction of specific reactive groups into RNA for cross-linking with peptides and proteins. In related DNA, many established reactive groups have been linked to nucleobases in 2’-deoxyribonucleoside triphosphates (dNTPs) and incorporated enzymatically^12^, e.g. Michael acceptors reacting with Cys^13^, chloroacetamide reacting with Cys or His^14^, and glyoxal^15^ or 1,3-diketones^16^ for Arg, as well as nitroso benzaldehyde^17^ or squaramate^18^ for Lys. On the other hand, there were only a few examples of protein-cross-linking modifications of RNA relying on 5-fluoro- or 5-azapyrimidine nucleotides that form covalent adducts with Cys-containing proteins, e.g. methyltransferases^19–21^. Other related examples of reactive modifications of RNA included posttranscriptional labelling through bioorthogonal reactions^22–29^ or alkylation of RNA with methyltransferases^30–33^. Recently, we reported chloroacetamide (CA)-linked ATP that can be incorporated into RNA by in vitro transcription (IVT) using T7 RNA polymerase (T7 RNAP) and cross-links to Cys- or His-containing peptides and proteins (Fig. 1A)^34^, but there is an urgent need for reactive groups and RNA probes specific for Lys as one of the most abundant amino acid in nucleic acids binding proteins^35–37^.

The well-established IVT with T7 RNAP using modified ribonucleoside triphosphates (NTPs) incorporates the modifications replacing natural counterparts at all sites^23–25,38–40^. Enzymatic site-specific single modification with RNA polymerases is very complicated^41^ and was previously reported only through laborious methods of limited efficiency, i.e. position-selective labeling by T7 RNAP (PLOR)^42^, splint ligation^43^ or use of the extended genetic alphabet^26–29^. Only very recently we^44^ and others^45^ have independently reported enzymatic synthesis of base-modified RNA using engineered TGK DNA polymerase and DNA template for extension of an RNA primer (PEX) and an expedient site-specific or segmented incorporation of one or more modifications through (repeated) single nucleotide incorporation followed by primer extension. This approach takes advantage of the high substrate tolerance and efficiency of DNA polymerases for incorporation of even highly modified NTPs and of the possibility to exchange single-stranded DNA template to synthesize specifically modified RNA not accessible by classical IVT.

In this paper, we present the design and development of Lys-reactive RNA probes through enzymatic synthesis by incorporation of the corresponding squaramate-modified ribonucleoside triphosphates (**N**^**ESQ**^**TPs**) with T7 RNAP or TGK DNA polymerase and applications in bioconjugations and cross-linking with Lys-containing peptides and proteins and studying inhibition of viral RNA polymerases (Fig. 1B).

## Results and discussion

### Synthesis of modified nucleosides and corresponding triphosphates

**Fig. 2:**
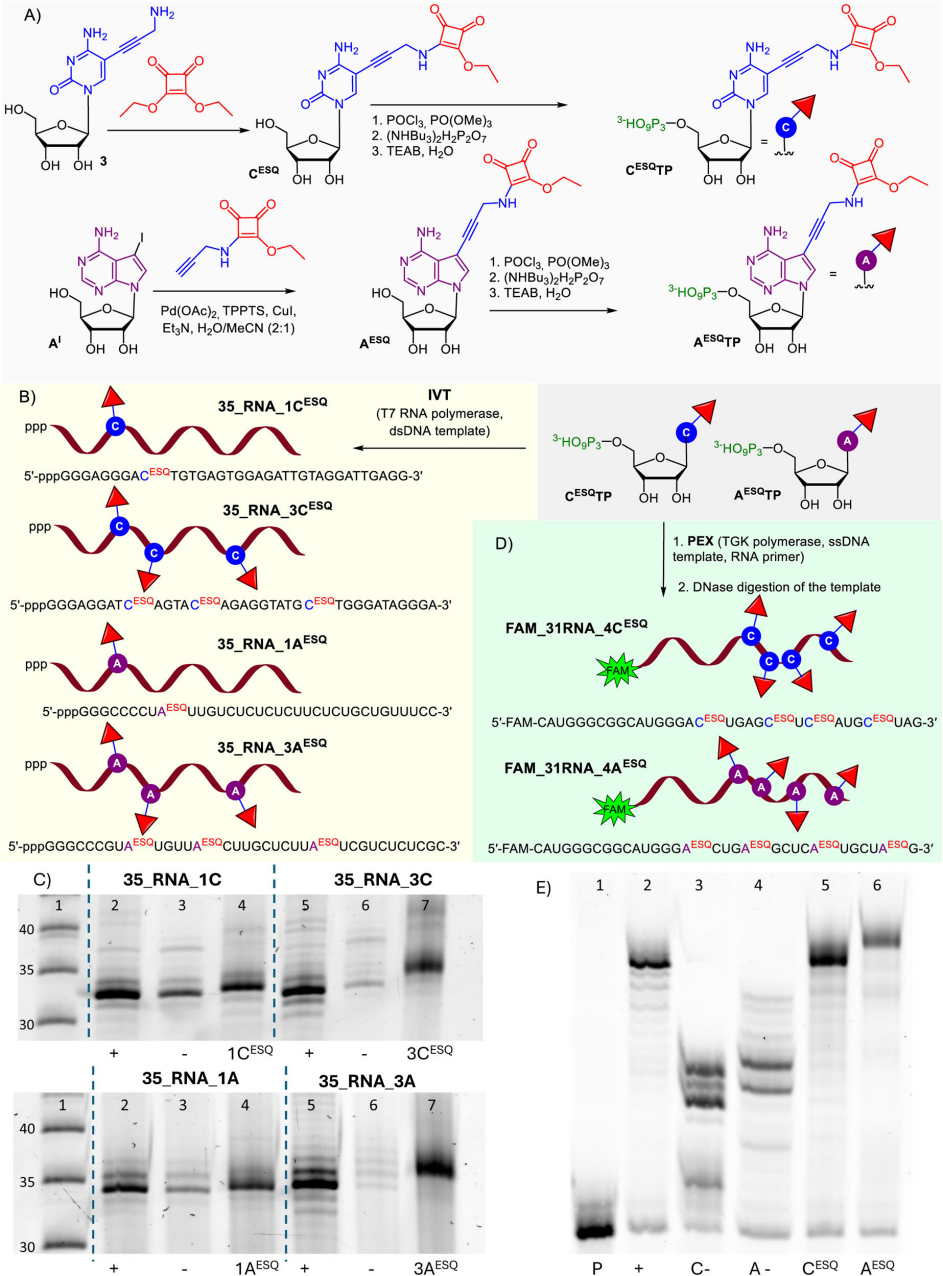
Synthesis of modified NTPs and RNA probes. **A** Synthesis of **C**^**ESQ**^**TP** and **A**^**ESQ**^**TP**. **B** IVT reactions with **C**^**ESQ**^**TP** or **A**^**ESQ**^**TP** and T7 RNAP. **C** PAGE analysis of IVT reactions. (+) positive controls with natural NTPs, (−) negative controls in the absence of CTP or ATP, (1C^ESQ^, 3C^ESQ^) IVT reactions with **C**^**ESQ**^**TP**, UTP, GTP, and ATP, (1A^ESQ^, 3A^ESQ^) IVT reactions with **A**^**ESQ**^**TP**, UTP, GTP, and CTP. **D** PEX reactions with **C**^**ESQ**^**TP** or **A**^**ESQ**^**TP** and TGK polymerase. **E** PAGE analysis of PEX reactions. (P) primer, (+) positive control with natural NTPs, (C−) negative control in the absence of CTP, (A−) negative control in the absence of ATP, (C^ESQ^) PEX reaction with **C**^**ESQ**^**TP**, UTP, GTP and ATP, (A^ESQ^) PEX reaction with **A**^**ESQ**^**TP**, UTP, GTP and CTP. For the uncropped gels see Supplementary Fig. 1.

We designed the squaramate-modified ribonucleoside triphosphates in a similar way to the previously reported dNTP. The squaramate was linked to position 5 of cytosine through a propargyl amide linker to ensure good compatibility with polymerase incorporation. The synthesis (Fig. S1 in SI and Fig. 2A) started by the Sonogashira reaction of 5-iodocytidine with *N*-propargyl trifluoroacetamide followed by deacylation to give 5-(3-aminopropyn-1-yl)-cytidine **3** in very good yield (87%). The reaction of nucleoside **3** with 2 equiv. of diethyl squarate gave squaramate-linked ribonucleoside (**C**^**ESQ**^) in acceptable yield (22%). The triphosphorylation^46^ of **C**^**ESQ**^ with POCl_3_ in trimethyl phosphate followed by treatment with tributyl ammonium pyrophosphate gave the corresponding **C**^**ESQ**^**TP** (38%). Additionally, we synthesized **A**^**ESQ**^**TP** bearing squaramate group at position 7 of 7-deazaadenine base tethered through aminopropagyl linker. The two-step synthesis (Fig. S2 in SI and Fig. 2A) started by the Sonogashira cross-coupling reaction of 5-iodo-adenosine (**A**^**I**^) with *N*-(propargyl)-squaramate (**PAS**) in the presence of Pd(OAc)_2_, CuI, TPPTS, and Et_3_N in a mixture of H_2_O/acetonitrile (2:1). Ethoxy squarate modified adenosine (**A**^**ESQ**^) was isolated after column chromatography in plausible yield (26%). Subsequent triphosphorylation reaction^43^ of **A**^**ESQ**^ gave the corresponding **A**^**ESQ**^**TP** (2%). The yield of **A**^**ESQ**^**TP** was significantly affected by the extensive purification procedures.

### Synthesis of ESQ-modified RNA by in vitro transcription using T7 RNAP

First, we tested the ability of **C**^**ESQ**^**TP** to serve as a CTP analog for T7 RNA polymerase in IVT. To describe the base generality, we also tested **A**^**ESQ**^**TP** as an ATP analog (Fig. 2B). We performed the IVT reactions in the presence of various DNA templates (for sequences, see Supplementary Table S1). The templates contained one, three, seven, or eight dG/dT residues in the coding region that guided the incorporation of one to eight **C**^**ESQ**^ or **A**^**ESQ**^ nucleotides into RNA transcript. We used two 5’-terminal 2’-MeO ribonucleotides of antisense strands of DNA templates to minimize non-templated nucleotide addition^47^. All transcription reactions were analyzed on a polyacrylamide gel and phosphor-imaged (Supplementary Fig. S3) or post-stained using Sybr-Gold (Fig. 2C, Supplementary Figs. S5–6). Since commercial transcription buffer supplied with the T7 RNAP contains spermidine and the squaramate group is known to readily react with primary amines^48^, we had to optimize the IVT conditions and composition of the buffers to prevent an undesired reaction of the ESQ-linked nucleotide or RNA with spermidine. Hence the spermidine was either replaced with quaternary amine betaine or we used a buffer with no amines (Supplementary Figs. S4, S7, S21, and S35). The IVT synthesis of RNA containing one or three **C**^**ESQ**^ or **A**^**ESQ**^ modifications (i.e. **35_RNA_1C**^**ESQ**^ or **35_RNA_3C**^**ESQ**^) proceeded smoothly with good conversions (Fig. 2C), whereas the formation of RNA with higher number of reactive modifications (i.e. **35_RNA_7C**^**ESQ**^) was less efficient (Supplementary Figs. S3, 4). In some cases, we have observed formation of minor amounts of n + 1 products of non-templated extension of the RNA products (Fig. 2C and Supplementary Figs. S3–6). In case of IVT with templates encoding for incorporation of just one modified nucleotide, the negative control experiments in the absence of the corresponding non-modified NTP also showed some formation of full-length product due to misincorporation of uridine, which is quite common in such cases^49^. In all cases, the identity of the full-length modified RNA products was also confirmed by MALDI-TOF (Supplementary Fig. S22-27) or LC-MS (Supplementary Figs. S33, 34, S37, 38). Additional control analysis of the reaction mixture on both native and denaturing protein gels excluded crosslinking of squaramate-modified RNA with the T7 RNA polymerase during the transcription reaction (Supplementary Fig. S13).

### Synthesis of ESQ-modified RNA by PEX using TGK polymerase

Following our recent paper on the enzymatic synthesis of base-modified RNA using engineered DNA polymerases^44^, we tested **C**^**ESQ**^**TP** and **A**^**ESQ**^**TP** as substrates for RNA synthesis by PEX using TGK polymerase (Fig. 2D)^50^. We used a 31-nt single-stranded DNA template encoding for four cytosines and four adenosines respectively in the complementary strand and a 5’-FAM-labelled 15-nt RNA primer (for sequences see Supplementary Table S1). The PEX reaction was followed by the digestion of the template by Turbo DNase, and we analyzed the products by polyacrylamide gel electrophoresis (PAGE) (Fig. 2E and Supplementary Fig. S8). The formation of natural **FAM_31RNA** and the desired modified **FAM_31RNA_4C**^**ESQ**^ and **FAM_31RNA_4A**^**ESQ**^ containing four modified **C**^**ESQ**^ or **A**^**ESQ**^ nucleotides was also confirmed by LC-MS (Supplementary Fig. S30-32, Supplementary Table S3). MS analysis revealed a non-templated addition of one additional GMP resulting in n + 1 longer RNAs.

### Conjugation reactions of ESQ-modified RNA with amine and Lys-containing peptide

**Fig. 3:**
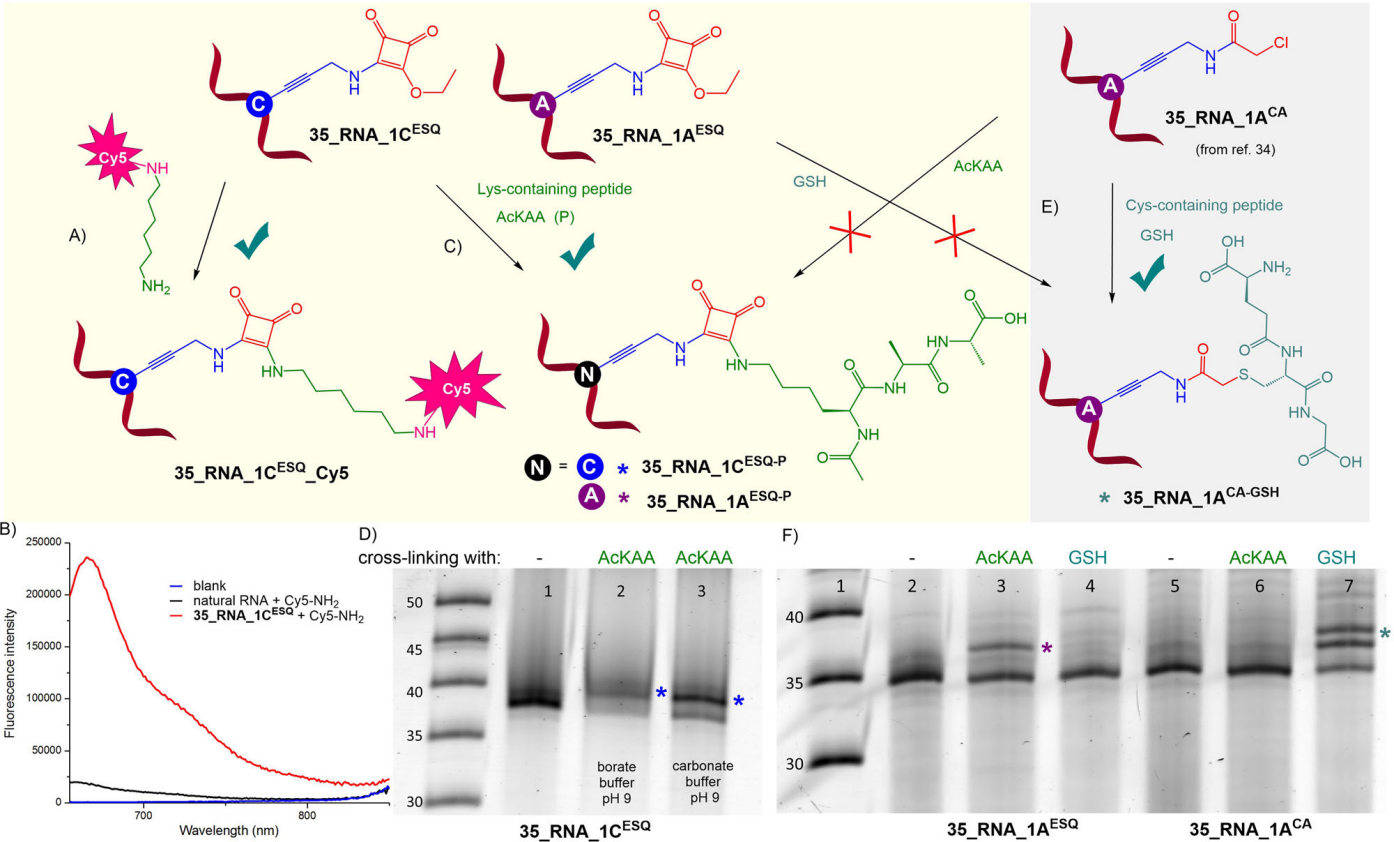
Reactivity and specificity of modified RNA probes. Cross-linking reaction scheme of ESQ-modified RNA with (A) amine-linked Cy5 fluorophore, (C) Lys-containing peptide, and (E) crossreactivity in comparison to CA-modified RNA with Cys-containing GSH. B) Fluorescence measurement of cross-linked **35_RNA_1C**^**ESQ**^**_Cy5** (red line) and non-cross-linked natural RNA (black line). D) PAGE analysis of cross-linking reactions of **35_RNA_1C**^**ESQ**^ with AcKAA peptide. (lane 1) **35_RNA_1C**^**ESQ**^; (lane 2) cross-linking reaction in borate or (lane 3) carbonate buffer. F) PAGE analysis of crosslinking reactions of **35_RNA_1A**^**ESQ**^ and **35_RNA_1A**^**CA**^ with AcKAA and GSH. (lane 2) **35_RNA_1A**^**ESQ**^, (lane 3) **35_RNA_1A**^**ESQ**^ with AcKAA, (lane 4) **35_RNA_1A**^**ESQ**^ with GSH, (lane 5) **35_RNA_1A**^**CA**^; (lane 6) **35_RNA_1A**^**CA**^ with AcKAA, (lane 7) **35_RNA_1A**^**CA**^ with GSH. For the uncropped gels see Supplementary Fig. 1.

First, we tested the reactivity of **C**^**ESQ**^-modified RNA with a primary amine. We used amine-linked Cy5 fluorophore (Fig. 3A, Supplementary Fig. S9) for site-specific RNA labelling. Non-labelled natural RNA (for control experiment) and squaramate-modified **35_RNA_1C**^**ESQ**^ were prepared by in vitro transcription followed by DNase I treatment and purified by spin columns. Model conjugation reaction was performed with amine-linked Cy5 fluorophore (100 - 400 equiv.) in buffer (pH 7.4-7.9) at 37 °C. After overnight incubation, the Cy5-labelled RNA conjugate was purified from the excess of the free dye and analyzed by fluorescence spectroscopy and fluorescence imaging of the denaturing PAGE. The strong fluorescence signal was observed only for cross-linked **35_RNA_1C**^**ESQ**^**_Cy5** while the non-modified RNA was not labelled under the same conditions (Fig. 3B, Supplementary Fig. S9a). The fluorescence imaging of the denaturing PAGE gel, shown in Supplementary Fig. S9b, supports the conclusion of fluorescence measurements that a stable covalent conjugate of Cy5 with **35_RNA_1C**^**ESQ**^ has been formed.

Then we tested the reactions of ESQ-modified RNAs with a Lys-containing peptide (AcKAA, P). We used **35_RNA_1C**^**ESQ**^ and **35_RNA_3C**^**ESQ**^ prepared by in vitro transcription using T7 RNA polymerase followed by DNase I treatment, purified and characterized by denaturing PAGE (Fig. 3C, D and Supplementary Fig. S5). Using **35_RNA_3C**^**ESQ**^ containing three reactive modifications, we observed formation of mixtures of conjugate products containing one, two or three peptides (Supplementary Fig. S11). We initially tested the influence of peptide excess (500 or 5000 equiv.) in borate buffer of different pH (9 or 10) for RNA-peptide conjugation efficacy. The buffer pH did not show any significant effect on the conjugation performance. On the other hand, increased excess of peptide pushed the conversion towards triply modified RNA-peptide conjugate but we never reached full conversion at all positions and we still observed a mixture of RNA-peptide conjugates with two and three peptide molecules attached. Having different electrophoretic mobilities, we could identify them on denaturing PAGE (Supplementary Fig. S10, S11b) and confirm by LC-MS (Supplementary Fig. S40-41, Supplementary Table S4).

We then used the higher peptide excess (9000 equiv.) in two different buffer systems (borate buffer, pH 9, and carbonate-bicarbonate buffer, pH 9.5) for the conjugation with **35_RNA_1C**^**ESQ**^ to yield the corresponding covalent conjugates **35_RNA_1C**^**ESQ-P**^ in 80 and 75% conversion, respectively (Fig. 3D, Supplementary Fig. S11a and S39). To compare the substrate specificity of squaramate-linked RNA probes, we performed a direct comparison experiment of cross-linking reactions of **A**^**ESQ**^-modified **35_RNA_1A**^**ESQ**^ with previously published chloroacetamide (CA)-modified RNA (**35_RNA_1A**^**CA**^)^34^. We used Cys-containing Glutathione (GSH) and the Lys-containing peptide in reactions with **35_RNA_1A**^**ESQ**^ and **35_RNA_1A**^**CA**^ (Fig. 3E, F, Supplementary Figs. S12a,S36). As expected, squaramate-modified **35_RNA_1A**^**ESQ**^ reacted only with Lys-containing peptide and not with GSH. On the other hand, the chloroacetamide-linked **35_RNA_1A**^**CA**^ reacted exclusively only with GSH showing the selectivity of both RNA probes towards specific amino acid residues. These results are supported by LC-MS measurements of both RNA-peptide and RNA-GSH conjugates (Supplementary Figs. S42, S43).

### Cross-linking of ESQ-modified RNA with proteins

**Fig. 4:**
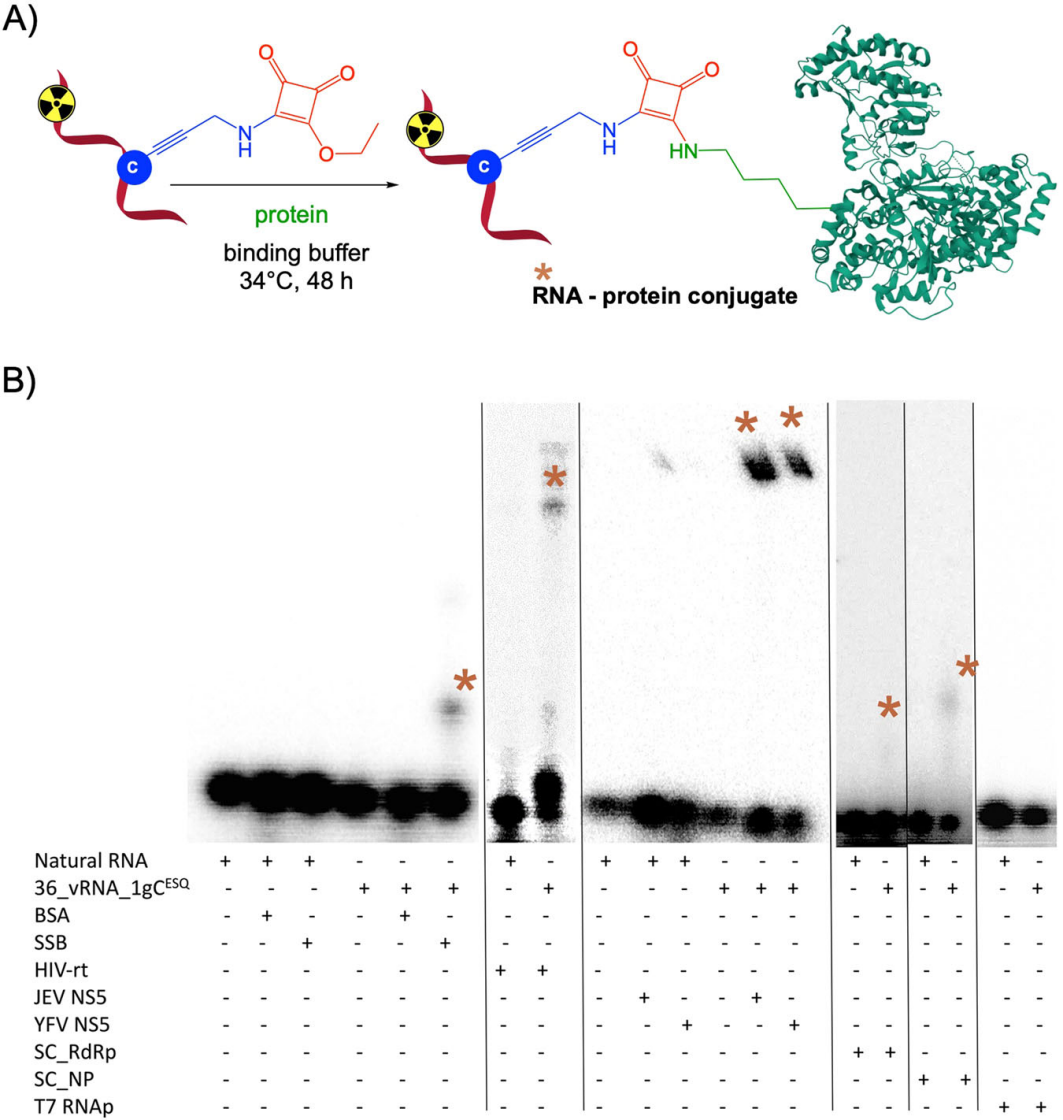
Reactivity of modified RNA probe with recombinant proteins. **A** Cross-linking of ESQ-modified RNA (**36_vRNA_1gC**^**ESQ**^) with Lys-containing protein. **B** 10% SDS-PAGE analysis of cross-linking experiments with natural or ESQ-modified RNA and various recombinant proteins (2–20 equiv. of protein; for the uncropped gels see Supplementary Fig. 1).

For cross-linking reactions of the ESQ-modified RNA with RNA binding proteins (RBP) we selected three RNA-dependent RNA polymerases (RdRp; Japanese encephalitis virus (JEV) NS5 protein, Yellow fever virus (YFV) NS5^51^ and SARS-CoV-2 RdRp (SC_RdRp))^52^, as well as SARS-CoV-2 nucleoprotein (SC_NP)^53^ and HIV reverse transcriptase (HIV-rt)^54^ as biologicaly relevant targets each of them containing several lysine amino acid residues. Bovine serum albumin (BSA) as an RNA non-binding protein was used as a negative control together with single-strand binding protein (SSB) having a 10-fold lower affinity for ssRNA compared to DNA^55^. For all RNA-protein crosslinking studies, radioactively labelled natural or ESQ-modified RNA was used (prepared by IVT reaction described in the method section). RNA-protein cross-linking reactions were performed at pH 7.4-8, with 2 equiv. of HIV-rt, 4 equiv. of JEV and YFV NS5 protein, 8 equiv. of SC_RdRp and SC_NP, and higher excess of RNA non-binding or RNA weakly binding proteins (20 equiv. of BSA and SSB) (Fig. 4A). The ability of proteins to bind **36_vRNA_1gC**^**ESQ**^ bearing one modification (for sequence see Supplementary Table S1) was monitored by electrophoretic mobility shift assay (EMSA).

Reaction analysis by 10% native PAGE confirmed that the squaramate modification did not prevent protein binding to RNA (Supplementary Figs. S14a, S15a, S17a, S18a). In all shown cases, except for incubation of RNA with T7 RNA polymerase, a band with slower mobility was observed, although the complex formation between RNA and SC_RdRp was very weak (Fig. 4B). As expected, no RNA-protein complexes were formed in the case of T7 RNAP and BSA (Fig. 4A, Supplementary Fig. S13 and S14). The denaturing SDS-PAGE revealed new bands with slower electrophoretic mobility indicating the formation of corresponding RNA-protein covalent cross-linked conjugates with moderate to good conversions (**RNA**^**ESQ_SSB**^ 9%, **RNA**^**ESQ_HIV_rt**^ 15%, **RNA**^**ESQ_JEV_NS5**^ 43%, **RNA**^**ESQ_YFV_NS5**^ 40%; Fig. 4B, Supplementary Figs. S14b, S18b, S15b, Table 1). The identity of the covalent **36_vRNA_1gC**^**ESQ_SSB**^ conjugate was confirmed by MS (Supplementary Fig. S44). Only traces of covalent cross-link were detected with SC_RdRp and SC_NP proteins. More squaramate modifications within the RNA strand in the case of **36_vRNA_3gC**^**ESQ**^ did not increase protein binding, nor the conversions (Supplementary Figs. S15, S17, Table S5). It should be noted that proximity effect is needed for an efficient cross-linking reaction and hence it is not the number of lysines but the accessibility and optimum positioning of one or more lysines in the vicinity to the reactive group is the driving force and decisive factor for the outcome of these reactions.

**Table 1:** Summary of cross-linking reactions between 36_vRNA_1gC^ESQ^ and proteins

Protein	Molecular weight [kDa]	RNA binding	No. of Lys residues	Conversion %
BSA	69.3	no	60	0%
T7 RNAP	99	no	66	0%
SSB	18.9	weakly	6	9%
HIV rt	117	yes	108	15%
JEV NS5	103.3	yes	58	43%
YFV NS5	103.3	yes	60	40%
SC_RdRp	62.2	yes	72	traces
SC_NP	45.6	yes	31	traces

Note: Conversions were determined from the gel using ImageJ quantificator.

### Gel-based RdRp polymerase assays in vitro

**Fig. 5:**
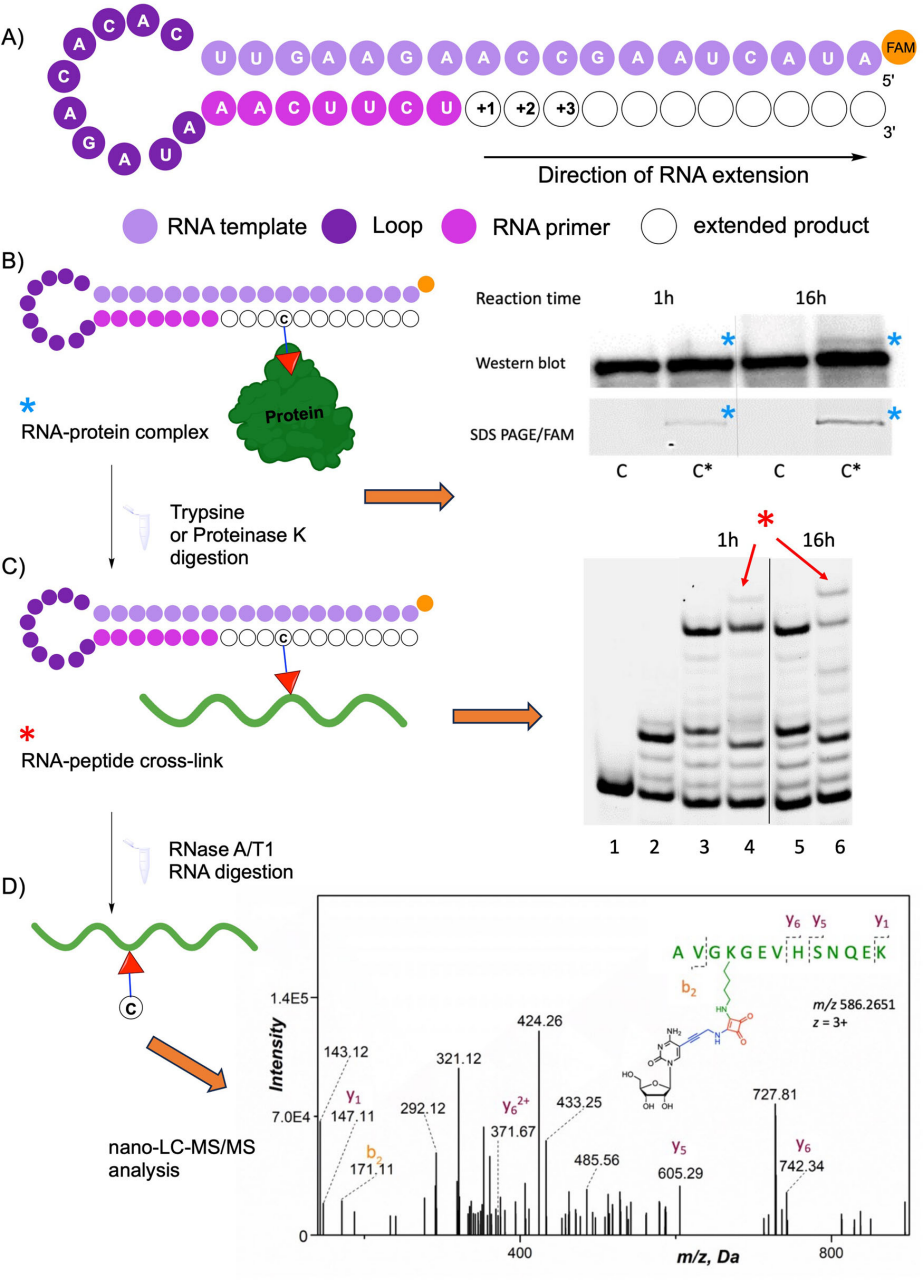
Summary of RdRp assay. **A** General scheme of RdRp polymerase assay. Hairpin RNA substrate with template-product scaffold. **B** Gel-based JEV RdRp assay analyzed by western blot and SDS-PAGE (lane C) positive control – reaction with natural NTPs; (lane C*) reaction with **C**^**ESQ**^**TP**, GTP, ATP, UTP. For the uncropped gels see Supplementary Fig. 1. **C** Proteinase K or Trypsin digestion of the RNA-protein conjugate; (lane 1) template; (lane 2) negative control – reaction without CTP; (lanes 3 and 5) positive control – reaction with natural NTPs; (lanes 4 and 6) reaction with **C**^**ESQ**^**TP**, GTP, ATP, UTP. **D** RNA digestion for nano LC-MS/MS analysis and spectrum of **46_RNA_HP**^**C4_JEVNS5**^ with identification of K269 (m/z acquired: 586.26 Da; Supplementary Fig. S45).

Following the cross-linking studies that have shown that the RdRps from *Flaviviridae* family readily cross-linked with squaramate-modified RNAs, we decided to investigate how the modified **C**^**ESQ**^**TP** influences the polymerase activity of the JEV recombinant RdRp (JEV_NS5) and SARS-CoV-2 RdRp (SC_RdRp) on an RNA template-product scaffold in vitro (Fig. 5A and Supplementary Figs. S19, S20). We used gel-based RdRp polymerase assay conditions similar to those published for various flaviviruses^56^ and non-modified templates that represent the 3’-end of the JEV genome of the c5’-UTR, that folds into a hairpin with 5’-FAM-labelled overhang as a template for primer extension (for sequences see Supplementary Table 2)^57^. RdRp polymerases are known to go through repeated cycles of abortive initiation before starting elongation mode^58^ and consecutive cytosines in the template may cause polymerase slippage^59^ which leads to the presence of short abortive products and ladder-like patterns on the gel. SARS-CoV-2 RdRp produced full-length ESQ-modified RNA, which was confirmed by PAGE (Supplementary Fig. S20) and MALDI (Supplementary Figs. S28, 29). On the other hand, in the case of the JEV recombinant RdRp, we observed the full-length product of natural RNA and only traces of squaramate-modified RNA (Supplementary Fig. S19a-b). After subsequent treatment of the reaction mixture with Proteinase K (pK) to release all RNA from the JEV NS5 protein-RNA complex, both natural and modified full-length RNAs were observed (Fig. 5C, lanes 3 and 4, respectively; Supplementary Fig. S19a, b). In the case of ESQ-modified RNA, an additional band of slower mobility was formed which intensity increased after prolonged reaction time (Fig. 5C, lane 6; Supplementary Fig. S19b). We assumed that this band might be the squaramate-modified RNA cross-linked to short peptide resulting from JEV NS5 protein digestion. Additionally, the presence of the cross-linked product of newly modified RNA and full-length JEV NS5 RdRp was independently proved by the comparison of 10% SDS protein gel visualized both by FAM for RNA and immunodetection of JEV NS5 (Fig. 5B; Supplementary Fig. S19d, e). These results suggest that the JEV RdRp incorporates **C**^**ESQ**^**TP** and further cross-links with produced ESQ-modified RNA during the extension reaction.

### Proteomic analysis of ESQ-modified RNA-JEV conjugate

To characterize cross-linked **46_RNA_HP**^**C4-JEV**^ conjugate and to determine the RNA-protein binding sites, two independent samples of the reaction mixture after polymerase assay were submitted to LC-MS/MS proteomic analysis (Fig. 5D; and Supplementary Fig. S45, 46) which revealed three lysine residues participating in protein cross-linking to **C**^**ESQ**^ in newly synthesized RNA (K269 in sample 1; K462 and/or K463 in sample 2). We identified fragments corresponding to cross-linking to neighbouring lysine residues in the spectrum of sample 2, and it can not be distinguished whether it is on K462 or K463. For sequence coverage of analyzed protein samples see Supplementary Fig. S47, 48 and Supplementary Table S6 in supporting material. The comparison with the published crystal structure of JEV RdRp (PDB 4K6M) reveals the location of the K269 in the 10-residue linker region (residues 266-275) which connects the MTase on its C-terminus (Supplementary Fig. S49). The sequence of this linker varies highly among genus flavivirus. The lysines K462 and K463 are in the ring finger domain of RdRp (residues 453-479) that forms the roof of the NTP entry channel at the -5 position from the NTP binding motif F (Phe467). These amino acids are highly conserved in flaviviruses^60^.

## Conclusions

Herein we report the synthesis of squaramate-modified ribonucleotides **C**^**ESQ**^**TP** and **A**^**ESQ**^**TP** and their incorporation into the RNA by in vitro transcription. We have found that the spermidine (primary amine) present in the commercial reaction buffer interfered with the ESQ moiety during the transcription reaction. After optimization of reaction conditions by exchanging the spermidine for quaternary betaine or using a buffer with no amine, full-length RNA transcripts containing one to eight **C**^**ESQ**^ and **A**^**ESQ**^ modifications were synthesized. We have also successfully used **C**^**ESQ**^**TP** and **A**^**ESQ**^**TP** in PEX for the synthesis of modified RNAs using engineered TGK DNA polymerase in analogy to our recent paper^44^. The ESQ-modified RNA was used in post-synthetic labelling with amine-linked Cy5 fluorophore and conjugation with short Lys-containing peptide. The reactions proceeded with high conversion only in the presence of a large excess of the amine or peptide. Squaramate moiety in RNA probes showed its specificity for cross-linking reactions with lysine-containing peptides and no reactivity with cysteine-containing peptides and thus is fully complementary to the previously reported^34^ chloroacetamide (CA) modification that reacts with Cys (and His) but not Lys. Most importantly, the ESQ-modified RNA probes reacted with various lysine-containing RNA-binding proteins (i.e., JEV and YFV NS5, and HIV-rt) and gave the covalently cross-linked conjugates in 9-43% conversions. On the other hand, negative control experiments with RNA non-binding or weakly binding proteins did not show any significant formation of covalent conjugates confirming that the proximity effect is crucial for good conversion. Finally, we investigated how **C**^**ESQ**^**TP** affects the polymerase activity of RNA-dependent RNA polymerases in vitro. Squaramate modification did not interfere with SARS-CoV-2 RdRp and gave full-length RNA. On the other hand, experimental results showed that newly synthesized RNA with incorporated **C**^**ESQ**^**TP** cross-linked with JEV RdRp during polymerase reaction. Proteomic analysis of the reaction mixtures of JEV RdRp RNA extension identified K269, K462, and K463 lysine residues modified by **C**^**ESQ**^ from modified RNA. Hence there is certain potential in using reactive modified nucleotides for inhibition of viral RNA polymerases, though probably not in the therapeutically relevant mode.

The squaramate moiety specifically reacts with primary amines including lysine to form stable amide bonds under physiological conditions without any external reagents and hence it has a good potential in post-synthetic labelling of RNA, bioconjugation of RNA with various biomolecules, cross-linking with proteins with abundant lysine or in developing irreversible inhibitors of RNA-binding enzymes containing free lysine residues (e.g., viral polymerases). It is complementary to the previously reported chloroacetamide reactive group^34^ and together or even in combination they can be used for RNA proteomics to identify new RNA-binding proteins.

## Methods

Complete experimental part including the chemical syntheses of modified nucleotides and their characterization, all enzymatic IVT and PEX reactions together with cross-linking reactions, and others are given in Supplementary Methods section in Supplementary Information.

### In vitro transcription with A^ESQ^TP and C^ESQ^TP

In vitro transcription reaction (10 µL) contained dsDNA template (1 µM, ssDNA strands annealed in 10 mM Tris, 50 mM NaCl, 1 mM EDTA, pH 7.8), natural NTPs (2 mM), **N**^**ESQ**^**TP** (1 mM for **C**^**ESQ**^**TP** or 2 mM for **A**^**ESQ**^**TP**), T7 RNA polymerase (2 U/µL) and buffer (40 mM Tris-HCl, 6 mM MgCl_2_, 10 mM DTT, 10 mM NaCl, pH 7.9). Water was used instead of **C**^**ESQ**^**TP** or **A**^**ESQ**^**TP** in the negative control experiments. The mixture was incubated at 37 °C for 5 h. When the reaction was completed, the dsDNA template was digested with DNase I (0.1 U/µL) for 1 h at 37 °C followed by the addition of EDTA (50 mM) and heated for 15 min at 75 °C. The reaction mixture was purified using Monarch Kit 50 µg for further use.

### PEX using TGK polymerase

PEX reaction (10 µL) contained DNA template Prb4basII (4.8 µM), FAM-labelled DNA primer Prim248-short (4 µM), natural NTPs (0.8 mM), **N**^**ESQ**^**TP** (0.8 mM), TGK polymerase (1.5 µM) and ThermoPol reaction buffer (1X). The mixture was heated at 95 °C for 30 sec and followed by 60 °C for 2 h. Positive control experiment was performed using natural CTP (0.8 mM) and negative control in the absence of **N**^**ESQ**^**TP**. Reactions were then treated with TurboDNase (2U) and heated at 37 °C for 30 min. The crude reaction mixture was purified using a QIAquick kit.

### Cross-linking reaction of ESQ-modified RNA (35_RNA_1A^ESQ^) and CA-modified RNA (35_RNA_1A^CA^) with AcKAA peptide and Glutathione

Cross-linking reaction (25 µL) contained **35_RNA_1A**^**ESQ**^ or **35_RNA_1A**^**CA**^ (2 µM), peptide AcKAA (10000 equiv.) or GSH (10000 equiv) and borate buffer (0.1 M, pH 9). The mixture was incubated at 37 °C for 48 h. Crude reaction mixtures were purified using a Monarch RNA cleanup kit.

### Cross-linking of ESQ-modified RNA with proteins NS5 of Japanese Encephalitis Virus (JEV) and Yellow Fever Virus (YFV)

Radioactively-labelled natural RNA and **36_vRNA_1gC**^**ESQ**^**, 36_vRNA_3fC**^**ESQ**^ (with one and three modifications respectively) were prepared by in vitro transcription as described above. Natural or modified RNA (0.5 µM) was incubated with 2 µM of NS5 protein (JEV resp. YFV) in 10X binding buffer (50 mM Tris-HCl of pH 7.4, 100 mM DTT, 5% Triton X-100, 10% glycerol; 2 µL), 10 mM MnCl_2_ (2 µL), 10 mM MgCl_2_ (2 µL), 50% glycerol (2 µL) and DEPC water (total reaction volume 20 µL) at 34 °C. After 1 hour 3 µL of the reaction mixture was separated by 5-7% native PAGE (acrylamide/bisacrylamide 37.5:1; 4 °C, 200 V, 1× Tris-glycine). The rest of the reaction was incubated for 48 h at 34 °C, then diluted with 2×VPS loading buffer, denatured for 10 min at 95 °C before loading, and analyzed by 5-10% SDS denaturing PAGE (acrylamide/bisacrylamide 37.5:1; 0.250 M Tris, 0.192 M glycine, 0.100% SDS) at room temperature (230 V, 70 min).

### SARS-CoV-2 RdRp mediated RNA extension assay^61^

The polymerase activity of SARS-CoV-2 RdRp was determined in a PEX reaction using fluorescently-labelled RNA templates. The reaction mixture (20 µL) contained a reaction buffer (10 mM Tris-HCl, pH. 8.0, 2 mM MgCl_2_, 10 mM KCl, 1 mM β ME), 10 μM NTPs, 0.5 µM template, 1 μM nsp12 and 3 μM nsp7/8 proteins. In the positive control, all natural NTPs were used and in the modified version 10 μM **C**^**ESQ**^**TP** instead of CTP was used. The reactions were incubated for 1 h at 30 °C. Reactions were stopped by adding of 2×PAGE stop solution and denatured at 95 °C for 10 min before loading. Samples were separated by 12.5% PAGE (acrylamide/bisacrylamide 19:1, 25% urea) under denaturing conditions (TBE 1×, 42 mA, 1 hour).

For MALDI TOF analysis, the reaction mixture was desalted with Bio-Spin6/Biorad columns (buffer was exchanged for water according to the supplied protocol).

### LC-MS characterization of ESQ-modified RNA and their corresponding peptide conjugates

LC-MS analyses were carried out using bioZen 1.7 µm Oligo, 2.1 × 150 mm column (Kinetex) using mobile phase A (15 mM Et_3_N, 300 mM HFIP in H_2_O) and mobile phase B (15 mM Et_3_N, 300 mM HFIP in MeOH) by 30 min gradient from 5% B to 50% B. Deconvolutions of LC-MS spectra were carried out using the UniDec program^62^.

## Supplementary information

Transparent Peer Review file

Supplementary information

Description of Additional Supplementary Files

Supplementary Data 1

Supplementary data 2

## Data Availability

Detailed procedures and data are given in Supplementary Information. NMR data are provided in Supplementary Data 1. Uncropped gels from Figs. 1–5 are provided in Supplementary Data 2. Primary data are available from repository: 10.48700/datst.6pjaf-g8g07.
