# Supplementary material for: Enzymatic synthesis of reactive RNA probes containing squaramate-linked cytidine or adenosine for bioconjugations and cross-linking with lysine-containing peptides and proteins
Source: Commun Chem. 2025 Jan 2;8:1. doi: 10.1038/s42004-024-01399-6 (PMC11696893; doi:10.1038/s42004-024-01399-6)
Supplement: Supplementary file 2 — Supplementary information [file 42004_2024_1399_MOESM2_ESM.pdf]

Supplementary information

Enzymatic Synthesis of Reactive RNA Probes Containing Squaramate-Linked Cytidine or Adenosine for Bioconjugations and Cross-Linking with Lysine-Containing Peptides and Proteins

Contents

1. Supplementary Methods	S2
1.1 Chemical synthesis	S2
1.1.1 General remarks for the synthetic part	S2
1.1.2 Synthesis and characterization of ESQ-modified cytidine and cytidine triphosphate	S3
1.1.3 Synthesis and characterization of ESQ-modified adenosine and adenosine triphosphate	S8
1.2 Biochemistry	S11
1.2.1 General remarks for the biochemistry part	S11
1.2.2 DNA and RNA oligonucleotides used in this study	S13
1.2.3 Synthesis of ESQ-modified RNA and its conjugation with amine-linked fluorescent label, peptides and proteins	S16
1.2.4 Gel-based polymerase assays in vitro	S36
1.2.5 Characterization of individual ethoxy squarate-modified RNAs and their cross-linking products with peptide and individual proteins by mass spectrometry	S40
2. Supplementary References	S66

1. Supplementary Methods

1.1 Chemical synthesis

1.1.1 General remarks for the synthetic part

NMR spectra were recorded on a Bruker Avance-IIIHD 500 (500.0 MHz for ^{1}H, 202.4 MHz for ^{31}P, 125.7 MHz for ^{13}C, and 376.5 MHz for ^{19}F) and Bruker Avance-IIIHD 400 (400.0 MHz for ^{1}H) spectrometer. ^{1}H and ^{13}C resonances were assigned using H, H-COSY, H,C-HSQC and H,C-HMBC experiments. The samples were measured in D_2O, CD_3OD or DMSO-d_6. Chemical shifts (δ scale, in ppm) were referenced as follows: D_2O (referenced to *t*-BuOH as internal standard: 1.24 ppm for ^{1}H NMR and 39.43 ppm ^{13}C NMR); CD_3OD (referenced to solvent signal: 3.31 ppm for ^{1}H NMR and 49.00 ppm for ^{13}C NMR); DMSO-d_6 (referenced to solvent signal: 2.50 ppm for ^{1}H NMR and 39.70 ppm for ^{13}C NMR). ^{31}P chemical shifts were referenced to H_3PO_4 as an external standard (0 ppm). Coupling constants (*J*) are given in Hz. High resolution mass spectra were measured on LTQ Orbitrap XL (Thermo Fisher Scientific) using ESI ionization technique and were performed by the MS service at IOCB. All materials were purchased from commercial suppliers (SigmaAldrich, Fluorochem) and used without further purification unless otherwise stated. $POCl_3$ and $PO(OMe)_3$ were distilled prior to use. The water used in synthetic part was of HPLC quality. Purification of nucleoside triphosphates was performed using HPLC (Waters) on a C18 reversed phase column (Phenomenex, Luna C18 (2) 100 Å).

1.1.2 Synthesis and characterization of ESQ-modified cytidine and cytidine triphosphate

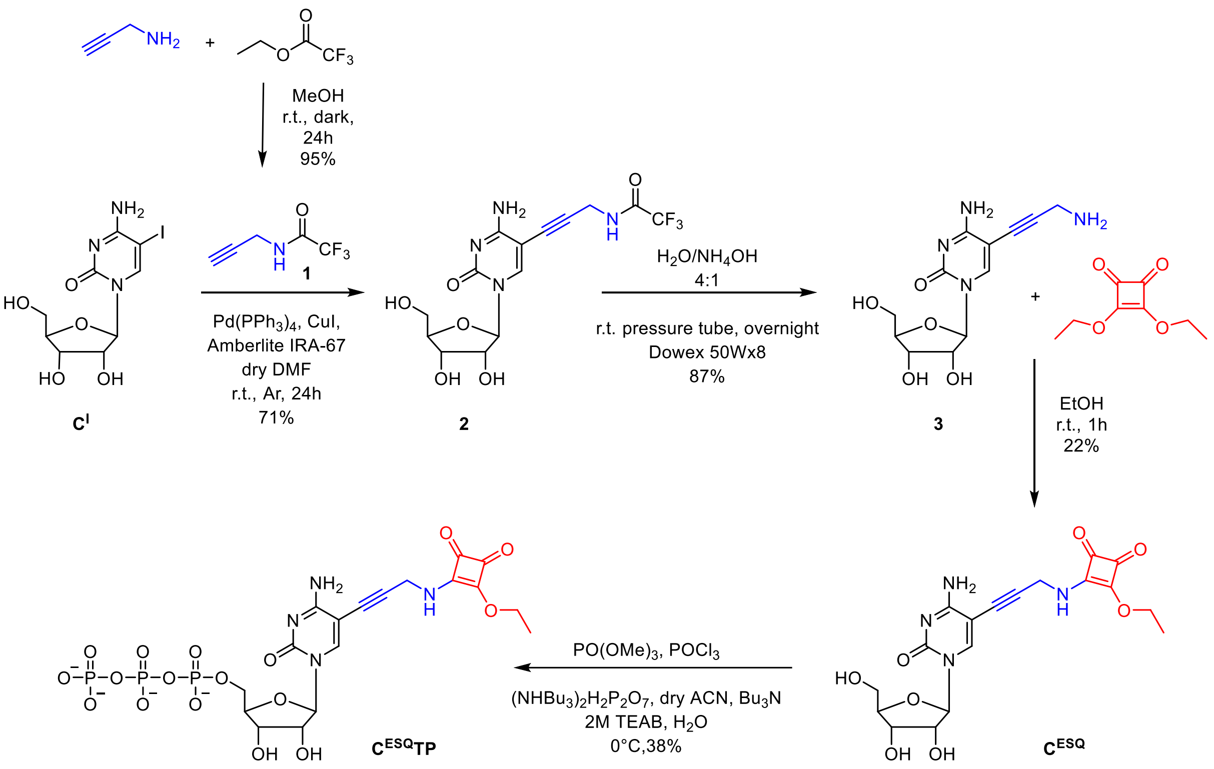

Figure S1. Chemical synthesis of ESQ-modified cytidine.

2, 2, 2-Trifluoro-*N*-(prop-2-ynyl)acetamide[1]

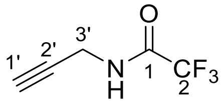

Propargylamine (1.50 g, 27.2 mmol) and ethyl trifluoroacetate (5.03 g, 35.4 mmol) were dissolved in methanol (50 mL) and stirred for 24 h in the dark at room temperature. After removal of the solvent under reduced pressure saturated $NaHCO_3$ solution (56 mL) was added to the residue and the aqueous phase was extracted with DCM (4 x 70 mL). The combined organic layers were dried over Na_2SO_4, filtered and the solvent was removed under reduced pressure. Desired product was obtained as yellow oil in 95% yield (3.89 g) and used without further purification.

All spectral data are consistent with the published literature.

5-[3-(Trifluoroacetamido)-prop-1-ynyl]-cytidine (C^{PACF3}) [2,3]

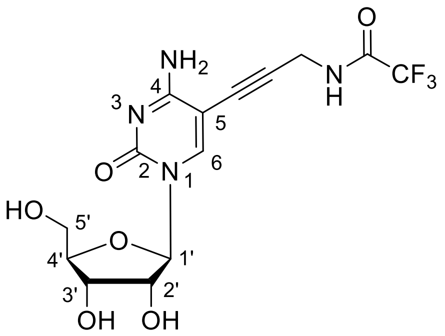

5-Iodo-cytidine (C^{I}; 0.443 g, 1.20 mmol), *N*-propargyl trifluoroacetamide (0.544 g, 3.60 mmol), CuI (0.045 g, 0.240 mmol), $Pd(PPh_3)_4$ (0.139 g, 0.120 mmol) and Amberlite Ira-67 resin (1.07 g) were placed in a 50 mL round bottom flask and dissolved in anhydrous DMF (6 mL) under argon atmosphere. The reaction mixture was stirred in the dark at r.t. for 24 hours then filtered over bed of SiO_2 topped with celite (eluted with mixture of DCM/methanol 7:1). The solvents were removed under reduced pressure and the title product was obtained by column chromatography using DCM/methanol (8:2) as a mobile phase in 71% yield (0.336 g) as an orange oil.

^{1}H NMR (400.1 MHz, CD_3OD): 3.76 (dd, 1H, J_{gem} = 12.4, $J_{5'b,4'}$ = 2.9, H-5′b); 3.91 (dd, 1H, J_{gem} = 12.4, $J_{5'a,4'}$ = 2.6, H-5′a); 4.03 (ddd, 1H, $J_{4',3'}$ = 5.6, $J_{4',5'}$ = 2.9, 2.6, H-4′); 4.09 – 4.18 (m, 2H, H-2′,3′); 4.31 (s, 2H, CH_2N); 5.84 (d, 1H, $J_{1',2'}$ = 2.9, H-1′); 8.41 (s, 1H, H-6).

^{13}C NMR (125.7 MHz, CD_3OD): 30.96 (CH_2N); 61.51 (CH_2-5′); 70.36 (CH-3′); 75.39 (**C**≡CCH_2); 76.37 (CH-2′); 85.83 (CH-4′); 90.92 (C≡**C**CH_2); 92.04 (C-5); 92.22 (CH-1′); 117.26 (q, $J_{C,F}$ = 286.5, **C**F_3CO); 146.43 (CH-6); 156.86 (C-2); 158.73 (q, $J_{C,F}$ = 37.5, CF_3**C**O); 166.30 (C-4).

^{19}F NMR (376.5 MHz, CD_3OD): -77.27.

HR/MS (ESI$^+$) for $C_{14}H_{16}O_6N_4F_3$: $[M + H]^+$ calcd 393.09, found 393.10.

5-(3-Amino-propynyl)- cytidine (C^{PA})

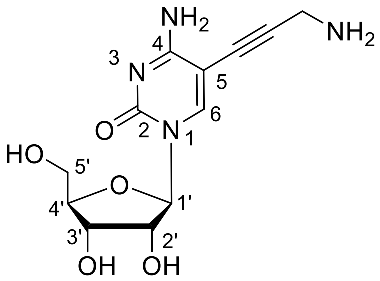

C^{PACF3} (0.336 g, 0.855 mmol) was dissolved in the mixture of 6 mL of HPLC water and 24 mL of aqueous ammonium hydroxide in the pressure tube and the tube was closed properly. The reaction was stirred overnight at r.t. and then concentrated down. The crude product was redissolved in HPLC H_2O (10 mL) and DOWEX 50x8 resin (2.50 g) was added. The mixture was stirred for 50 min and filtered over a bed of Dowex 50x8 resin (2.50 g) which was then washed with HPLC H_2O and the product was eluted off the resin with HPLC H_2O/conc. NH_4OH (4:1, 400 mL). Final product was obtained as orange-brown oil (0.219 g, 87%) after removal of the solvents under reduced pressure.

^{1}H NMR (400.1 MHz, CD_3OD): 3.62 (s, 2H, CH_2N); 3.76 (dd, 1H, J_{gem} = 12.4, $J_{5'b,4'}$ = 2.8, H-5′b); 3.91 (dd, 1H, J_{gem} = 12.4, $J_{5'a,4'}$ = 2.5, H-5′a); 4.03 (ddd, 1H, $J_{4',3'}$ = 5.6, $J_{4',5'}$ = 2.8, 2.5, H-4′); 4.08 – 4.17 (m, 2H, H-2′,3′); 5.84 (d, 1H, $J_{1',2'}$ = 2.9, H-1′); 8.37 (s, 1H, H-6).

HR/MS (ESI$^+$) for $C_{12}H_{16}O_5N_4Na$: $[M + Na]^+$ calcd 319.10, found 319.10.

4-[5- (3-Amino-propynyl) -cytidine]-3- ethoxycyclobut-3-ene-1,2-dione (C^{ESQ})

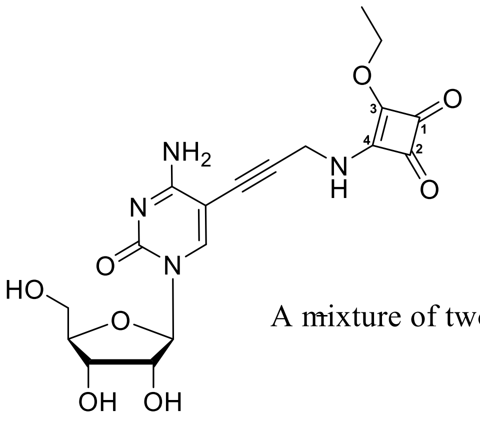

A mixture of two isomers 1:1

$\mathbf{C^{PA}}$ (0.120 g, 0.405 mmol) was well suspended in 2 ml of ethanol. Diethyl squarate (0.120 mL, 0.810 mmol) was added dropwise during 30 min to the stirred solution and the reaction mixture was stirred another 1 hour at room temperature. Column chromatography using DCM/methanol (8:2) as a mobile phase gave the desired product as white powder in 22% yield (0.037 g).

^{1}H NMR (500.0 MHz, DMSO-d_6): 1.37 (t, 6H, J_{vic} = 7.1, C**H3**CH2O); 3.55, 3.68 (2 × ddd, 2 × 2H, J_{gem} = 12.1, $J_{5',OH}$ = 4.9, $J_{5',4'}$ = 2.9, H-5′); 3.83 (dt, 2H, $J_{4',3'}$ = 5.2, $J_{4',5'}$ = 2.9, H-4′); 3.91 – 3.96 (m, 4H, H-2′,3′); 4.37, 4.56 (2 × bs, 2 × 2H, CH_2N); 4.66 (q, 4H, J_{vic} = 7.1, $CH_3C\mathbf{H_2}O$); 4.49 (m, 2H, OH-3′); 5.15 (t, 2H, $J_{OH,5'}$ = 4.9, OH-5′); 5.36 (m, 2H, OH-2′); 5.75 (d, 2H, $J_{1',2'}$ = 3.5, H-1′); 6.82, 6.84 (2 × bs, 2 × 1H, $NH_a\mathbf{H_b}$); 7.84 (s, 2H, $N\mathbf{H_a}H_b$); 8.26 (s, 2H, H-6); 8.95, 9.11 (2 × bs, 2 × 1H, NH).

^{13}C NMR (125.7 MHz, DMSO-d_6): 15.17 ($\mathbf{C}H_3CH_2O$); 34.06, 34.51 (CH_2N); 60.29 (CH_2-5′); 69.19 (CH-3′); 69.28 ($CH_3\mathbf{C}H_2O$); 74.48 (CH-2′); 76.42, 76.55 (cyt-**C**≡C-CH_2); 84.45 (CH-4′); 89.12 (C-5); 89.95 (CH-1′); 90.22, 90.82 (cyt-C≡**C**-CH_2); 145.08 (CH-6); 153.83 (C-2); 164.43 (C-4); 171.80, 172.47 (C-4-cyclobut); 177.33, 177.76 (C-3-cyclobut); 182.47, 182.79, 188.92, 189.48 (C-1,2-cyclobut).

HR/MS (ESI$^+$) for $C_{18}H_{21}O_8N_4$: $[M + H]^+$ calcd 421.13, found 421.13.

4-{[5- (3-Amino-propynyl) -cytidine]-5'-*O*-triphosphate}-3- ethoxycyclobut-3-ene-1,2-dione (C^{ESQ}TP)

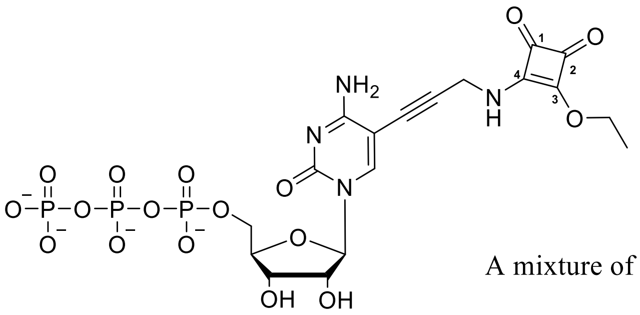

A mixture of two isomers 1:1

Ethoxy squarate modified nucleoside (**C^{ESQ}**, 0.031 g, 0.074 mmol) was dried at 80 °C for 2 hours in vacuo. After cooling, $PO(OMe)_3$ (0.240 mL) and $POCl_3$ (8.40 µL) were added on ice under argon atmosphere. The reaction mixture was stirred for 90 min at 0 °C. In a separate flask, the mixture of $(NHBu_3)H_2P_2O_7$ (0,220 g) and Bu_3N (54 µL) in dry acetonitrile (0.600 mL) was prepared under argon atmosphere, cooled to 0 °C and then added by the syringe to the reaction mixture. The mixture was stirred for 1 hour at 0 °C. The reaction was stopped by addition of TEAB (2 M, 0.400 mL) and water (1.19 mL). The product was purified by C18 reversed-phase HPLC using water/methanol (5 to 50%) containing 0.1 M TEAB buffer as eluent. Several co-distillations with water and conversion to sodium salt (Dowex 50Wx8 in Na^+ cycle) followed by freeze-drying from water gave the product **C^{ESQ}TP** as white powder in 38% yield (0.202 g).

^{1}H NMR (500.0 MHz, D_2O): 1.45 (bt, 6H, J_{vic} = 7.0, C**H**$_3$CH$_2$O-A,B); 4.25 – 4.33 (m, 8H, H-2′,4′,5′); 4.40 (t, 2H, $J_{3',2'}$ = $J_{3',4'}$ = 5.4, H-3′); 4.57, 4.67 (2 × bs, 2 × 2H, CH$_2$N); 4.70 – 4.85 (m, 4H, CH$_3$C**H$_2$**O, overlapped with water signal); 5.94 (d, 2H, $J_{1',2'}$ = 3.8, H-1′); 8.25 (s, 2H, H-6). ^{13}C NMR (125.7 MHz, D_2O): 17.87 (**C**H$_3$CH$_2$O); 37.20, 37.46 (CH$_2$N); 67.26 (d, $J_{C,P}$ = 4.4, CH$_2$-5′); 71.51 (CH-3′); 73.64 (CH$_3$**C**H$_2$O); 77.26 (CH-2′); 78.04 (cyt-**C**≡C-CH$_2$); 85.51 (d, $J_{C,P}$ = 9.0, CH-4′); 92.54 (CH-1′); 93.37, 93.79 (cyt-C≡**C**-CH$_2$); 94.79 (C-5); 148.09 (CH-6); 159.03 (C-2); 167.86 (C-4); 175.84, 176.01 (C-4-cyclobut); 180.21, 180.88 (C-3-cyclobut); 186.61, 186.67, 191.50, 191.88 (C-1,2-cyclobut).

^{31}P{^{1}H} NMR (202.4 MHz, D_2O): -21.94, -21.89 (2 × bt, J = 19.7, 2P, P$_\beta$); -10.64 (d, 2P, J = 19.7, P$_\alpha$); -8.10 (br, 2P, P$_\gamma$).

HR/MS (ESI$^-$) for $C_{18}H_{22}O_{17}N_4P_3$: [M - H]$^-$ calcd 659.02, found 659.02.

1.1.3 Synthesis and characterization of ESQ-modified adenosine and adenosine triphosphate

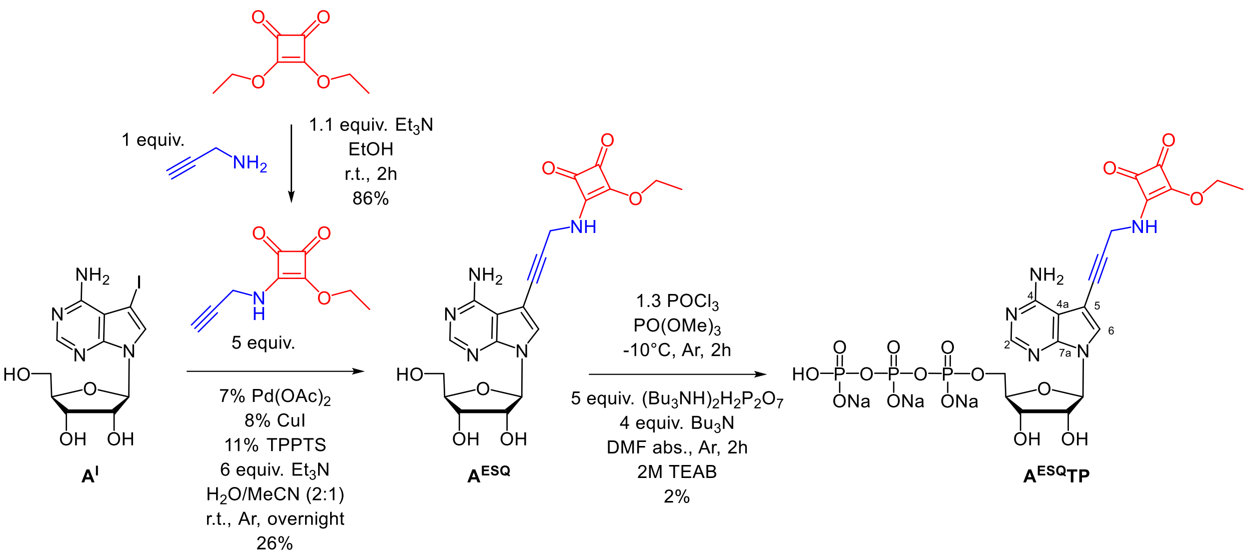

Figure S2. Chemical synthesis of ESQ-modified ATP.

3-Ethoxy-4-(prop-2-yn-1-ylamino)cyclobut-3-ene-1,2-dione (PAS)

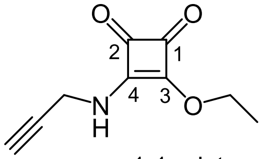

Diethyl squarate (0.870 mL, 5.88 mmol) was dissolved in EtOH (5 mL), and Et_3N (0.890 mL, 6.42 mmol) was added. Propargylamine (0.380 mL, 5.88 mmol) was added dropwise within 2 h at room temperature. Column chromatography using DCM/MeOH gave the desired propargyl-amino-squarate (**PAS**) as a yellow powder in 86% yield (0.910 g).

^{1}H NMR (500.0 MHz, DMSO-d_6): 1.37 (t, 6H, J_{vic} = 7.1, C**H**$_3$CH$_2$O); 3.35 (bt, 2H, ^4J = 2.4, HC≡C); 4.10, 4.31 (2 × bs, 2 × 2H, CH$_2$N); 4.56 (q, 4H, J_{vic} = 7.1, CH$_3$C**H$_2$**O); 8.96, 9.14 (2 × bs, 2 × 1H, NH).

^{13}C NMR (125.7 MHz, DMSO-d_6): 15.61 (**C**H_3CH_2O); 32.68, 33.09 (CH_2N); 69.06 (CH_3**C**H_2O); 75.35, 75.46 (H**C**≡C); 79.58, 79.96 (HC≡**C**); 171.80, 172.62 (C-4); 177.26, 177.55 (C-3-cyclobut); 182.49, 182.72, 188.69, 188.98 (C-1,2-cyclobut).
HR/MS (ESI$^+$) for $C_9H_9NO_3$: $[M+H]^+$ calcd 180.06, found 180.06.

3-((3-(4-Amino-7-((2*R*,3*R*,4*S*,5*R*)-3,4-dihydroxy-5-(hydroxymethyl)tetrahydrofuran-2-yl)-7*H*-pyrrolo[2,3-*d*]pyrimidin-5-yl)prop-2-yn-1-yl)amino)-4-ethoxycyclobut-3-ene-1,2-dione (A^{ESQ})

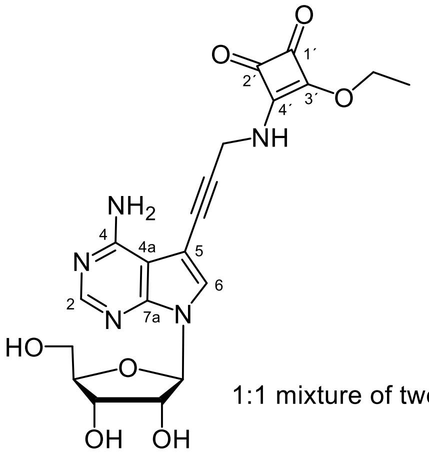

5-Iodo-adenosine (**A^I**, 0.300 g, 765 mmol), $Pd(OAc)_2$ (0.118 g, 54 mmol), CuI (0.117 g, 61 mmol), TPPTS (0.048 g, 84 mmol) and **PAS** (0.411g, 2.29 mol) was suspended in 6 mL of H_2O/MeCN (2:1). After the addition of Et_3N (0.640 mL, 4.59 mol), the reaction mixture was stirred overnight at room temperature. Column chromatography, after additional trituration with water, gave the desired product as white powder in 26% yield (0.088 g).
^{1}H NMR (500.0 MHz, DMSO-d_6): 1.38 (t, 6H, J_{vic} = 7.1, C**H$_3$**CH_2O); 3.53 (ddd, 2H, J_{gem} = 11.9, $J_{5'b,OH}$ = 6.2, $J_{5'b,4'}$ = 3.7, H-5′b); 3.62 (ddd, 2H, J_{gem} = 11.9, $J_{5'a,OH}$ = 5.0, $J_{5'a,4'}$ = 3.7, H-5′a); 3.89 (td, 2H, $J_{4'5'}$ = 3.7, $J_{4',3'}$ = 3.3, H-4′); 4.08 (ddd, 2H, $J_{3',2'}$ = 5.0, $J_{3',OH}$ = 4.8, $J_{3',4'}$ = 3.3, H-3′); 4.36 (ddd, 2H, $J_{2',OH}$ = 6.3, $J_{2',1'}$ = 6.0, $J_{2',3'}$ = 5.0, H-2′); 4.42, 4.61 (2 × bs, 2 × 2H, CH_2N); 4.64 – 4.72 (bm, 4H, CH_3C**H$_2$**O); 5.12 (d, 2H, $J_{OH,3'}$ = 4.8, OH-3′); 5.19 (dd, 2H, $J_{OH,5'}$ = 6.2, 5.0, OH-5′); 5.33 (d, 2H, $J_{OH,2'}$ = 6.3, OH-2′); 6.01 (d, 2H, $J_{1',2'}$ = 6.0, H-1′); 7.78 (s, 2H, H-6); 8.12 (s, 2H, H-2); 9.07, 9.25 (2 × bs, 2 × 1H, NH).
^{13}C NMR (125.7 MHz, DMSO-d_6): 15.66 (**C**H_3CH_2O); 33.86, 34.27 (CH_2N); 61.49 (CH_2-5′); 69.14 (CH_3**C**H_2O); 70.52 (CH-3′); 74.05 (CH-2′); 77.28, 77.49 (**C**≡C-CH_2); 85.26 (CH-4′); 87.12 (C≡**C**-CH_2); 87.26 (CH-1′); 87.60 (C≡**C**-CH_2); 94.05 (C-5); 102.21 (C-4a); 127.19 (CH-6); 149.65

(C-7a); 152.74 (CH-2); 157.51 (C-4); 171.74, 172.44 (C-4-cyclobut); 177.22, 177.71 (C-3-cyclobut); 182.36, 182.60, 188.82, 189.30 (C-1,2-cyclobut).

HR/MS (ESI$^+$) for $C_{20}H_{21}N_5O_7$: $[M+H]^+$ calcd 444.14, found 444.15.

Sodium ((2*R*,3*S*,4*R*,5*R*)-5-(4-amino-5-(3-((2-ethoxy-3,4-dioxocyclobut-1-en-1-yl)amino)prop-1-yn-1-yl)-7*H*-pyrrolo[2,3-*d*]pyrimidin-7-yl)-3,4-dihydroxytetrahydrofuran-2-yl)methyl hydrogen triphosphate (A^{ESQ}TP)

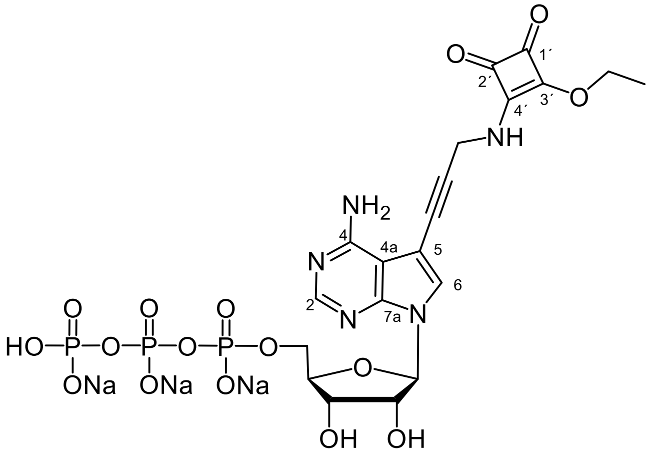

Ethoxy squarate modified nucleoside (**A^{ESQ}**, 0.051 g, 115 mmol) was dried at 50 °C for 30 min in a vacuum. After cooling with ice and NaCl, $PO(OMe)_3$ (1 mL) and $POCl_3$ (14 µL, 150 mmol) were added under an argon atmosphere. The reaction mixture was stirred for 2 h at -10 °C. A mixture of $(Bu_3NH)_2H_2P_2O_7$ (0.316 g, 575 mmol) and Bu_3N (0.110 mL, 460 mmol) in dry DMF (1 mL) was added to the reaction mixture and stirred for another 2 h at -10 °C. The reaction was stopped by addition of TEAB (2 M, 3 mL), evaporated and purified on C18 Kinetex EVO (Phenomenex) column using 0.1 M TEAB in water (phase A) and 0.1 M TEAB in 50% MeOH followed by purification using Sepharose column in water (phase A) to 0.8 M TEAB (phase B) and then another C18 Kinetex EVO column. After several co-distillations with water, conversion to sodium salt by Dowex (50Wx8 in Na^+ form), and freeze-drying, product **A^{ESQ}TP** was isolated as a white powder in 2% yield (1.90 mg). Yield was significantly affected by the extensive purification procedures.

^{1}H NMR (500.0 MHz, D_2O, ref(*t*BuOH) = 1.24 ppm): 1.44 (t, 3H, J_{vic} = 7.1, C**H$_3$**CH$_2$O); 4.15 (ddd, 1H, J_{gem} = 11.8, $J_{H,P}$ = 4.6, $J_{5'b,4'}$ = 3.1, H-5′b); 4.29 (ddd, 1H, J_{gem} = 11.8, $J_{H,P}$ = 6.9, $J_{5'a,4'}$ = 2.9, H-5′b); 4.35 (m, 1H, H-4′); 4.58 – 4.63 (m, 2H, H-3′, CH$_a$**H$_b$**N); 4.67 – 4.72 (m, 2H, H-2′, C**H$_a$**H$_b$N); 4.73 – 4.83 (m, 2H, CH$_3$C**H$_2$**O, overlapped with water signal); 6.25 (d, 1H, $J_{1',2'}$ = 6.8, H-1′); 7.86 (s, 1H, H-6); 8.18 (s, 1H, H-2).

^{13}C NMR (125.7 MHz, D_2O, ref(*t*BuOH) = 32.43 ppm): 17.88 (br, **C**H_3CH_2O); 37.44 (br, CH_2N); 68.14 (d, $J_{C,P}$ = 5.8, CH_2-5′); 73.32 (CH-3′); 73.68 (br, CH_3**C**H_2O); 76.68 (CH-2′); 79.98, 80.15 (br, deazaA-**C**≡C-CH_2); 86.80 (d, $J_{C,P}$ = 8.7, CH-4′); 88.59 (CH-1′); 90.41 (br, deazaA-C≡**C**-CH_2); 99.03 (C-5); 105.99 (C-4a); 129.80 (br, CH-6); 152.36 (C-7a); 155.48 (CH-2); 160.55 (C-4); C-1,2,3,4-cyclobut not detected.
^{31}P{^{1}H} NMR (202.4 MHz, D_2O): -21.97 (dd, *J* = 20.1, 19.6, P_β); -11.12 (d, *J* = 19.6, P_α); 5.99 (d, *J* = 20.1, P_γ).
HR/MS (ESI$^-$) for $C_{20}H_{21}N_5Na_3O_{16}P_3$: [M-H]$^-$ calcd 747.99, found 747.98.

1.2 Biochemistry

1.2.1 General remarks for the biochemistry part

Mass spectra of oligonucleotides were measured on UltrafleXtreme MALDI-TOF/TOF (Bruker) mass spectrometer with 1 kHz smartbeam II laser. Mass spectra of oligonucleotides for conjugations with peptide were measured on Agilent 1290 Infinity II Bio system with DAD detector and mass spectrometer MSD XT. UV-Vis spectra were measured at room temperature on NanoDrop1000 (ThermoScientific). Fluorescence was measured on a Fluoromax 4 spectrofluorimeter (HORIBA Scientific). Samples were concentrated on CentriVap Vacuum Concentrator system (Labconco). Synthetic DNA oligonucleotides (**Table S1**) were purchased from Generi Biotech (Czech Republic) or Eurofins Genomics (Germany). Synthetic RNA (**Table S2**) oligonucleotides were purchased from Sigma Aldrich. Sulfo-Cyanine5 amine from Lumiprobe. [α-^{32}P]-GTP from M.G.P. spol. s r.o. Natural nucleoside triphosphates (ATP, GTP, UTP, CTP), BSA, T7 RNA polymerase, Ribolock RNase inhibitor, Monarch RNA cleanup kit and HiScribe T7 High Yield RNA Synthesis kit were purchased from New England Biolabs. SSB protein was purchased from Sigma Aldrich. DNase I and Ultra Low Range DNA Ladder were purchased from ThermoFisher Scientific. Sars-Cov-2 nucleoprotein from ProteoGenix and HIV-rt from Merck Millipore. Tripeptide Acetyl-Lys-Ala-Ala was purchased from Merck Life Science. QIAquick® Nucleotide Removal Kit from Qiagen, NucAway spin columns from Ambion and Bio-Spin 6 from Biorad. Japanese encephalitis virus NS5 polyclonal antibody and goat anti-rabbit IgG (H + L) secondary antibody, HRP were purchased from Invitrogen. Immobilon-P transfer membrane from Millipore. Western blott detection reagent SuperSignal West Femto

Chemiluminescent Substrate from Thermo Scientific. PageBlue™ protein staining solution from ThermoFisher Scientific. Milli-Q water was used for all experiments. For all RNA experiments DEPC water was used.

JEV NS5, YFV NS5 and SC nsp12, nsp7/8 were expressed as described before.[4,5] Briefly, SC nsp7/8 complex and flaviviral NS5 proteins were expressed in bacterial cells (*E. coli* BL-21 CodonPlus (DE3)-RIL), except for the nsp12 protein that was expressed in insect cells (*Sf*9 cell line from *Spodoptera frugiperda*). Subsequently, the recombinant proteins were purified using Ni^{2+} affinity chromatography followed by tag cleavage and further purified using size exclusion chromatography.

Buffer composition (10X):

Buffer A: 400 mM Tris-HCl (pH 7.9), 60 mM $MgCl_2$, 100 mM DTT, 100 mM NaCl, 20 mM spermidine.

Buffer B: 400 mM Tris-HCl (pH 7.9), 60 mM $MgCl_2$, 100 mM DTT, 100 mM NaCl, 20 mM betain.

Buffer C: 400 mM Tris-HCl (pH 7.9), 60 mM $MgCl_2$, 100 mM DTT, 100 mM NaCl.

Buffer D: 100 mM Tris (pH. 8), 20 mM $MgCl_2$, 100 mM KCl, 12 mM βME.

Buffer E: 50 mM Tris-HCl (pH. 7.4), 100 mM DTT, 5% Triton X-100, 10% glycerol.

PAGE stop solution contains: 80% [v/v] formamide, 20 mM EDTA, 0.0250% [w/v] bromophenol blue and 0.0250% [w/v] xylene cyanol.

VPS loading buffer used for protein SDS gels contains: 0.0500 M TRIS (pH 6.8), 17% glycerol, 16 mM mercaptoethanol (βME), 3.50% SDS, bromophenol blue.

1.2.2 DNA and RNA oligonucleotides used in this study

Table S1. Sequences of DNA templates, RNA primer and RNA transcripts.[a]

	Sequence	Length
52_DNA_1C	5′-TAATACGACTCACTATAGGGAGGGACTGTGAGTGGAGATTGTAGGATTGAGG-3′	52
	3′-ATTATGCTGAGTGATATCCCTCCCT**G**ACACTCACCTCTAACATCCTAACT[mC][mC]-5′	52
**35_RNA_1C**^{ESQ}	5′-pppGGGAGGGA**C**^{ESQ}UGUGAGUGGAGAUUGUAGGAUUGAGG-3′	35
52_DNA_3C	5′-TAATACGACTCACTATAGGGAGGATCAGTACAGAGGTATGCTGGGATAGGGA-3′	52
	3′-ATTATGCTGAGTGATATCCCTCCTA**G**TCAT**G**TCTCCATAC**G**ACCCTATCCT[mC][mU]-5′	52
**35_RNA_3C**^{ESQ}	5′-pppGGGAGGAU**C**^{ESQ}AGUA**C**^{ESQ}AGAGGUAUG**C**^{ESQ}UGGGAUAGGGA-3′	35
52_DNA_7C	5′-TAATACGACTCACTATAGGGGATGTCTGAGCCTAAGGATGCCAACTGTTGTC-3′	52
	3′-ATTATGCTGAGTGATATCCCCTACA**G**ACTC**GG**ATTCCTAC**GG**TT**G**ACAAC[mA][m**G**]-5′	52
**35_RNA_7C**^{ESQ}	5′-pppGGGGAUGU**C**^{ESQ}UGAG**C**^{ESQ}**C**^{ESQ}UAAGGAUG**C**^{ESQ}**C**^{ESQ}AA**C**^{ESQ}UGUUGU**C**^{ESQ}-3′	35
53_DNAv_8C	5′-TAATACGACTCACTATAGATACTAAGCCAAGAAGTTCACACAGATAAACTTCT-3′	53
	3′-ATTATGCTGAGTGATATCTAT**G**ATTC**GG**TTCTTCAA**G**T**G**T**G**TCTATTT**G**AA[m**G**][mA]-5′	53
**36_vRNA_8C**^{ESQ}	5′-pppGAUA**C**^{ESQ}UAAG**C**^{ESQ}**C**^{ESQ}AAGAAGUU**C**^{ESQ}A**C**^{ESQ}A**C**^{ESQ}AGAUAAA**C**^{ESQ}UU**C**^{ESQ}U-3′	36
52_DNAv_1eC	5′-TAATACGACTCACTATAGATAGTAAGGCATAAATTGAGAGAGATAAATTTAT-3′	52
	3′-ATTATGCTGAGTGATATCTATCATTCC**G**TATTTAACTCTCTCTATTTAAA[mU][mA]-5′	52

**35_vRNA_1eC^{ESQ}**	5′-pppGAUAGUAAGG**C**^{ESQ}AUAAAUUGAGAGAGAUAAA<br>UUUAU-3′	35
53_DNAv_3fC	5′-TAATACGACTCACTATAGATACTAAGCCAATAAA<br>TTGAGAGAGATAAATTTAT-3′<br>3′-ATTATGCTGAGTGATATCTAT**G**ATTC**GG**TTATTT<br>AACTCTCTCTATTTAAA[mU][mA]-5′	53<br><br>53
**36_vRNA_3fC^{ESQ}**	5′-<br>pppGAUA**C**^{ESQ}UAAG**C**^{ESQ}**C**^{ESQ}AAUAAAUUGAGAGAGAUAA<br>AUUUAU-3′	36
53_DNAv_1gC	5′-TAATACGACTCACTATAGATACTAAGGGAATAAA<br>TTGAGAGAGATAAATTTAT-3′<br>3′-ATTATGCTGAGTGATATCTAT**G**ATTCCCTTATTT<br>AACTCTCTCTATTTAAA[mU][mA]-5′	53<br><br>53
**36_vRNA_1gC^{ESQ}**	5′-pppGAUA**C**^{ESQ}UAAGGGAAUAAAUUGAGAGAGAUAAA<br>UUUAU-3′	36
52_DNA_1A	5′TAATACGACTCACTATAGGGCCCCTATTGTCTCTCTCTT<br>CTCTGCTGTTTCC-3′<br>3′-ATTATGCTGAGTGATATCCCGGGGA**T**AACAGAGAG<br>AGAAGAGACGACAAA[mG][mG]-5′	52<br><br>52
**35_RNA_1A^{ESQ}**	5´pppGGGCCCCU**A**^{ESQ}UUGUCUCUCUCUUCUCUGCUGUUU<br>CC-3´	35
**35_RNA_1A^{CA}**	5´pppGGGCCCCU**A**^{CA}UUGUCUCUCUCUUCUCUGCUGUUUC<br>C-3´	35
52_DNA_3A	5′TAATACGACTCACTATAGGGCCCGTATGTTACTTGCTCT<br>TATCGTCTCTCGC-3′<br>3′-ATTATGCTGAGTGATATCCCGGGCA**T**ACAA**T**GAACGAG<br>AA**T**AGCAGAGAG[mC][mG]-5′	52<br><br>52
**35_RNA_3A^{ESQ}**	5´pppGGGCCCGU**A**^{ESQ}UGUU**A**^{ESQ}CUUGCUCUU**A**^{ESQ}UCGUC<br>UCUCGC-3´	35
Prb4basII	5′-C**TAG**CA**TGAG**C**T**CA**GT**CCCATGCCGCCCATG-3′	31
Prim248-short	5′-FAM-CAUGGGCGGCAUGGG-3′	15
**FAM_31RNA_4C^{ESQ}**	5′CAUGGGCGGCAUGGGA**C**^{ESQ}UGAG**C**^{ESQ}U**C**^{ESQ}AUG**C**^{ESQ}U<br>AG-3′	31

**FAM_31RNA_4A^{ESQ}**	5′CAUGGGCGGCAUGGG**A**^{ESQ}CUG**A**^{ESQ}GCUC**A**^{ESQ}UGCU**A**^{ESQ} G-3′	31

[a] Promoter region in the antisense strand is underlined. The two 5’-terminal nucleotides in the antisense strand were 2’-MeO ribonucleotides (m) to minimize non-templated nucleotide addition. The templated bases directing transcription of modified bases are marked bold and the modified bases in the transcripts are marked bold in red.

Table S2. Sequences of RNA templates and RNA products used in *in vitro* RdRp polymerase assay.[a]

	Sequence (5' → 3')	Length
35_RNA_tHP^{C4}	5′-[6FAM]AUACUAA**G**CCA**AGAAGUUCACACAGAUAAA CUUCU**-3′	35
**46_RNA_HP^{C4}**	5′-[6FAM]AUACUAA**G**CCA**AGAAGUUCACACAGAUAAA CUUCU**UGG**C**UUAGUAU-3′	46
**46_RNA_HP^{C4_ESQ}**	5′-[6FAM]AUACUAA**G**CCA**AGAAGUUCACACAGAUAAA CUUCU**UGG**C**^{ESQ}UUAGUAU-3′	46
29_RNA_tSC^{C1}	5′-[6FAM]AACAAAACAU**G****CGCGUAGUUUUCUACGCG**-3′	29
**40_RNA_SC^{C1}**	5′-[6FAM]AACAAAACAU**G****CGCGUAGUUUUCUACGC G****C**AUGUUUUGUU-3′	40
29_RNA_tSC^{C4}	5′-[6FAM]AACAAAA**G**AUC**CGCGUAGUUUUCUACGCG**-3′	29
**40_RNA_SC^{C4}**	5′-[6FAM]AACAAAA**G**AUC**CGCGUAGUUUUCUACGC G**GAU**C**UUUUGUU-3′	40

[a] Hairpin part is marked bold in blue, base that is directing position of modified base is marked bold, whereas the modified base in the product is marked bold in red.

1.2.3 Synthesis of ESQ-modified RNA and its conjugation with amine-linked fluorescent label, peptides and proteins

In vitro transcription with $\mathbf{C^{ESQ}TP}$

A solution of template DNA oligonucleotides (100 µM each) in 1:1 ratio in annealing buffer [Tris (10 mM, NaCl (50 mM), EDTA (1 mM), pH 7.8] was heated to 95 °C for 5 minutes and slowly cooled to 25 °C in 45 minutes. The resulting DNA (50 µM) was used as a template for transcription reactions.

In vitro transcription reactions were performed in the total volume of 20 µL containing: dsDNA template (2 µM), ATP (1 mM), UTP (1 mM), GTP (0.800 mM), CTP (1 mM) for positive control, $C^{ESQ}TP$ (1.60 mM) for modified RNA, [α-^{32}P]-GTP (111 TBq mmol^{-1}, 370 MBq mL^{-1}, 0.200 µL), T7 RNA polymerase (2.50 U µL^{-1}), $MgCl_2$ (4.80 mM), dithiothreitol (DTT; 12 mM), Triton X-100 (0.120%), dimethyl sulfoxide (DMSO; 5%) and 1x transcription buffer B (40 mM Tris-HCl (pH 7.9), 6 mM $MgCl_2$, 10 mM DTT, 10 mM NaCl, 2 mM betain). Water was used instead of the solution of $\mathbf{C^{ESQ}TP}$ in the negative control experiment. The mixture was incubated at 37 °C for 2 h. The aliquots (3 µL) were mixed with 2×PAGE stop solution, denatured (75 °C, 10 min) and analyzed by 12.5% PAGE (7 M urea, AA/bisAA 19:1, 40 mA/1h, 1×TBE). Visualization was performed by phosphoimaging (**Figure S3**).

DNase I treatment

The DNA template was digested by DNase I to attain pure RNA. Transcription mixture (25 µL), 4 µL of DNase I buffer (supplied with the enzyme), 4 µL of DNAse I (4 U), and 7 µL DEPC water were incubated 60 min at 37 °C. The enzyme was heat deactivated at 75 °C for 10 min followed with cooling on ice. All samples were purified on NucAway Spin Columns (50 µL) for further use.

Note

When performing transcription reaction with the buffer supplied with T7 RNAp by manufacturer (buffer A), newly synthetized squaramate modified RNA (**35_RNA_1C$^{\mathbf{ESQ}}$**) reacted with spermidine from the buffer solution. Comparative transcription reaction analysis in the presence of spermidine, betaine and without any amine present in the reaction buffer is shown in the **Figure S4**.

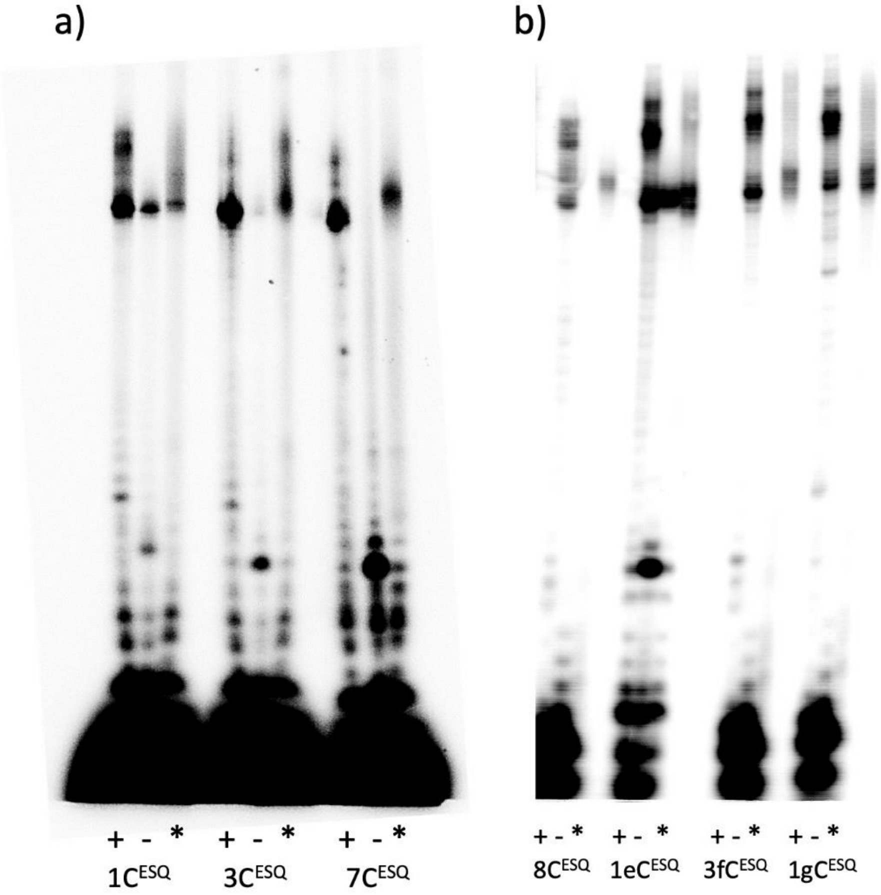

Figure S3. Phosphor image of transcripts obtained by *in vitro* transcription in the presence of CTP (+), **C^{ESQ}TP** (*) and in their absence for negative control (-) using transcription buffer with betaine and templates a) **52_DNA_1/3/7C** or b) templates designed for JEV 3'-end region of the c5'-UTR.

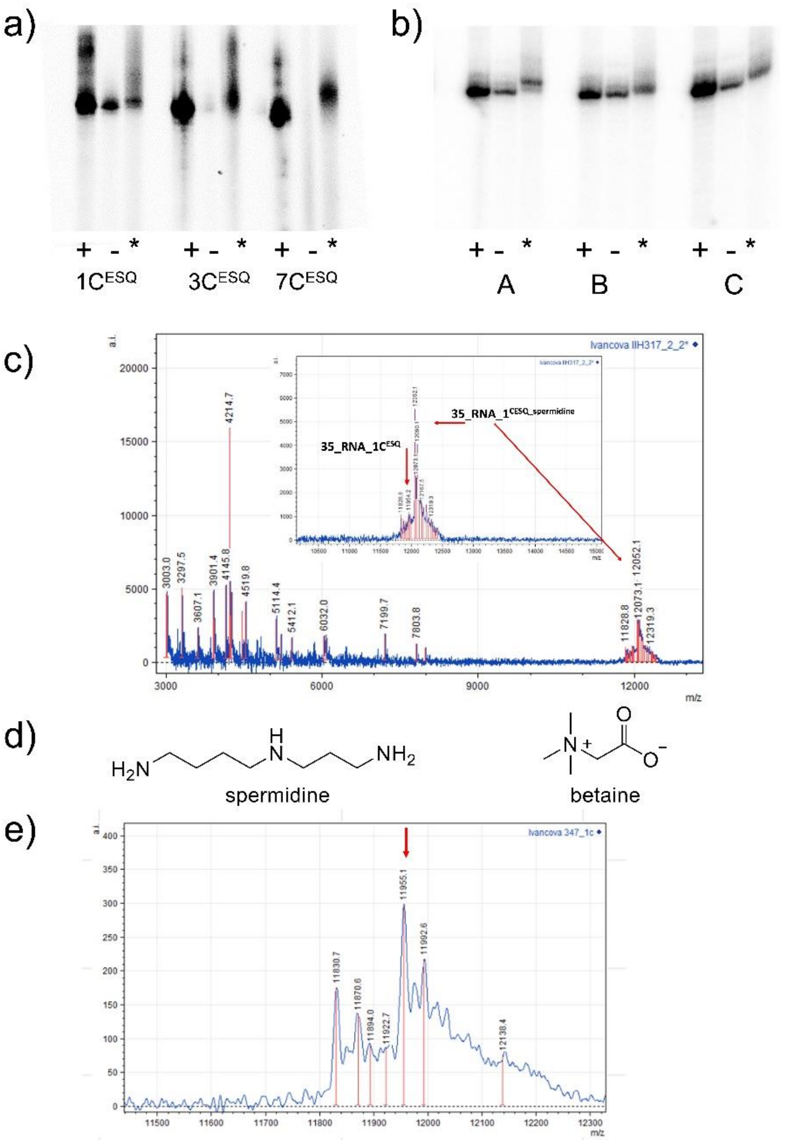

Figure S4. a) Phosphor image of transcripts obtained by *in vitro* transcription in the presence of CTP (+), **C^{ESQ}TP** (*) and in their absence for negative control (-) using the commercial T7RNAp buffer. b) Phosphor image of transcripts obtained by *in vitro* transcription using buffer supplied by manufacturer containing spermidine (A), buffer with betaine (B) and without any amine (C). c) MALDI-TOF MS spectrum of **35_RNA_1C^{ESQ}** after transcription reaction in commercial T7 RNAP reaction buffer. M (calc.) = 11954 Da, M (found) = 11954.2 Da. The peak at *m/z* = 12052.1 can be assigned to the **35_RNA_1C^{ESQ}** reacted with spermidine (M = 145.25 Da) from reaction buffer. d) Structures of amines used in transcription buffers. e) MALDI-TOF MS spectrum of **35_RNA_1C^{ESQ}** obtained after transcription reaction with buffer B. M (calc.) = 11954 Da, M (found) = 11955.1 Da $[M + H]^+$.

RNA synthesis by *in vitro* transcription with A^{ESQ}TP and C^{ESQ}TP using T7 RNAP

Reactions were performed in a total volume of 10 µL. Reaction mixtures contained dsDNA template (1 µM, ssDNA strands annealed in 10 mM Tris, 50 mM NaCl, 1 mM EDTA, pH 7.8, **Table S1**), three natural NTPs (2 mM for **35_RNA_1A^{ESQ}** and **35_RNA_3A^{ESQ}**; 1mM for **35_RNA_1C^{ESQ}** and **35_RNA_3C^{ESQ}**), **A^{ESQ}** (2 mM for **35_RNA_1A^{ESQ}** and **35_RNA_3A^{ESQ}** or **C^{ESQ}TP** (1 mM for **35_RNA_1C^{ESQ}** and **35_RNA_3C^{ESQ}**), T7 RNA polymerase (2 U µL^{-1}) and buffer (40 mM Tris-HCl, 6 mM $MgCl_2$, 10 mM DTT, 10 mM NaCl, pH 7.9). Positive control experiments contained natural NTPs (2mM for **35_RNA_1A** and **35_RNA_3A**; 1mM for **35_RNA_1C** and **35_RNA_3C**). Water was used instead of **C^{ESQ}TP** or **A^{ESQ}TP** in the negative control experiments. The mixture was incubated at 37 °C for 5 h. When the reaction was completed, the dsDNA template was digested with DNase I (0.1 U µL^{-1}) for 1 h at 37 °C followed by the addition of EDTA (50 mM) and heated for 15 min at 75 °C. The reaction mixture was purified using Monarch Kit 50 µg and the product was characterized by LC-MS (**Table S4**) and by 20% PAGE post-stained by Sybr-Gold (**Figure S5, S6**).

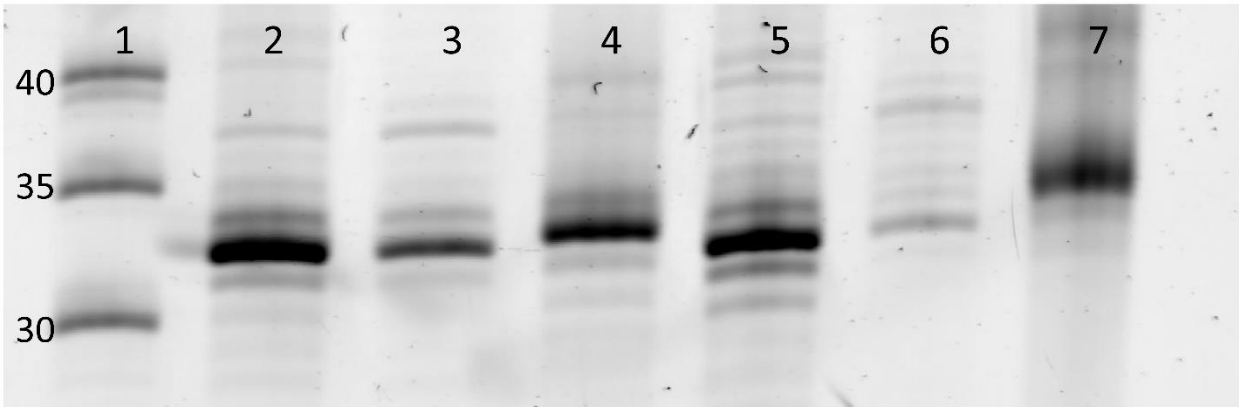

Figure S5. 20% denaturing PAGE of transcription reaction with **C^{ESQ}TP**. RNA ladder (lane 1); positive control with natural CTP, **35_RNA_1C** (lane 2); negative controls in the absence of **C^{ESQ}TP** (lanes 3 and 6); **35_RNA_1C^{ESQ}** (lane 4); positive control with natural CTP, **35_RNA_3C** (lane 5); **35_RNA_3C^{ESQ}** (lane 7).

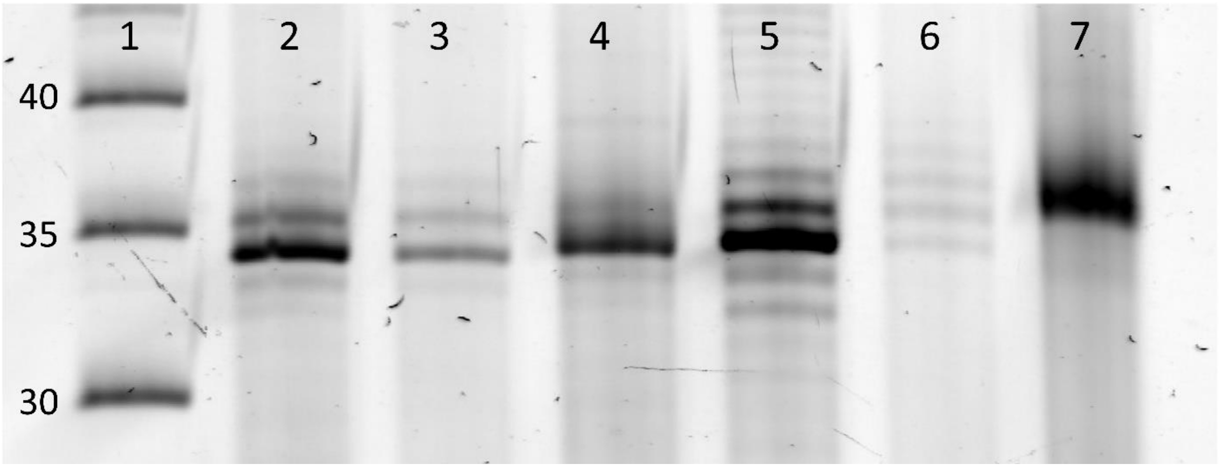

Figure S6. 20% denaturing PAGE of transcription reaction with **A^{ESQ}TP**. RNA ladder (lane 1); positive control with natural ATP, **35_RNA_1A** (lane 2); negative controls in the absence of **A^{ESQ}TP** (lanes 3 and 6); **35_RNA_1A^{ESQ}** (lane 4); positive control with natural ATP, **35_RNA_3A** (lane 5); **35RNA_3A^{ESQ}** (lane 7).

Note

As well in this case newly synthetized squaramate modified RNA reacted with spermidine from the supplied buffer solution. Comparative transcription reaction analysis in the presence of spermidine, betaine and without any amine present in the reaction buffer is shown in the **Figure S7**.

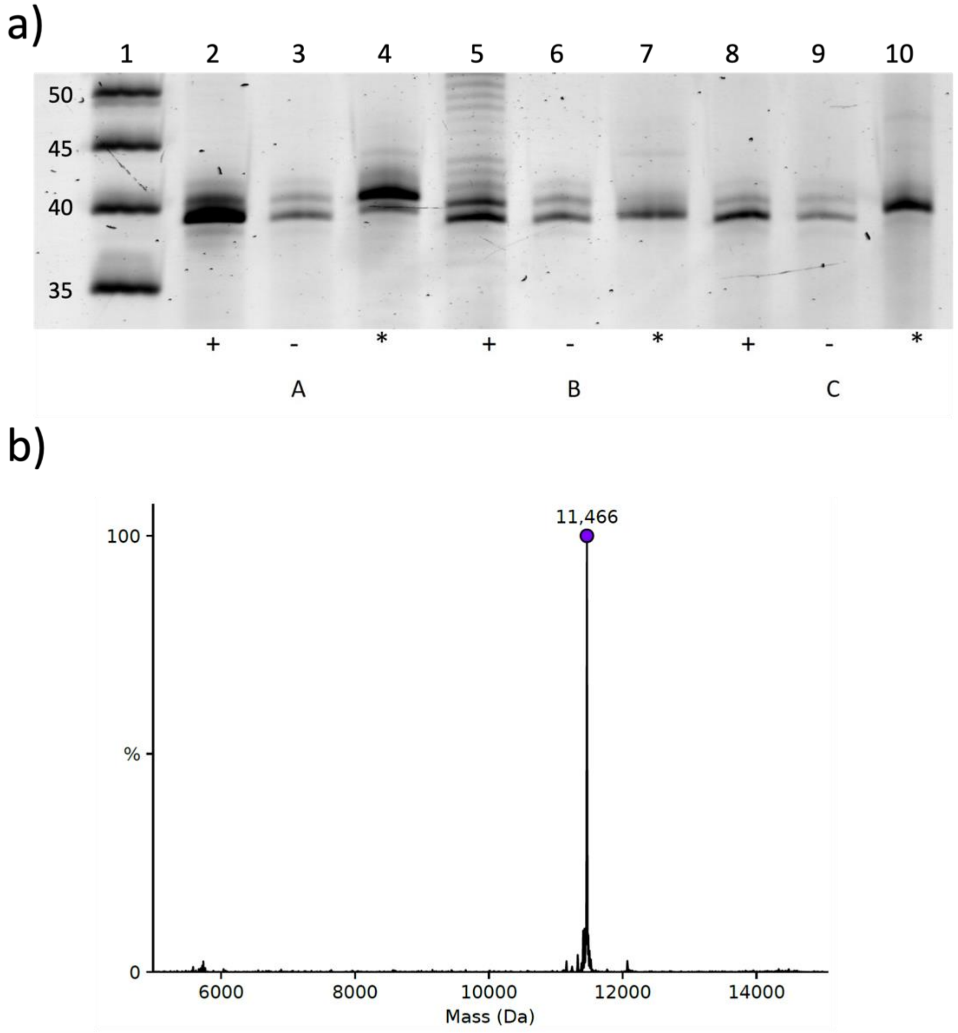

Figure S7. a) 20% denaturing PAGE analysis of transcription reactions using commercial T7 RNAp buffer containing primary amine - spermidine (A), home-made buffer with quarternary amine - betaine (B) and home-made buffer with no amine (C). RNA ladder (lane 1); **35_RNA_1A** as positive control with natural ATP (+); negative control in the absence of $\mathbf{A^{ESQ}}$ (-); transcription with $\mathbf{A^{ESQ}TP}$ (*). b) MS spectrum of $\mathbf{35\_RNA\_1A^{ESQ\_spermidine}}$ – RNA reacted with buffer containing amine (the intense band of lane 4), M (calc.) = 11461 Da, M (found) 11466 Da. MS spectrum of $\mathbf{35\_RNA\_1A^{ESQ}}$ from reaction with no amine (lane 10) is shown on **Figure S34**.

RNA Synthesis using TGK polymerase

Reactions were performed in a total volume of 10 µL. Reaction mixtures containing ssDNA template Prb4basII (4.80 µM final concentration, **Table S1**), FAM-labelled RNA primer Prim248-short (4 µM final, **Table S1**), three natural NTPs (0.800 mM final concentration), **C^{ESQ}TP** or **A^{ESQ}TP** (0.8 mM final concentration), TGK polymerase (1.5 µM final concentration) and ThermoPol reaction buffer (1×final concentration) were heated at 95 °C for 30 sec and followed by 60 °C for 2 h. Positive control was performed using natural CTP/ATP (0.800 mM final concentration) and negative control in the absence of **C^{ESQ}TP** or **A^{ESQ}TP**. Reactions were then treated with TurboDNase (2U) and heated at 37 °C for 30 min. Crude reaction mixture was purified using QIAquick (Qiagen) kit and characterized by 20% denaturing PAGE (**Figure S8**) and LC-MS (**Table S3**).

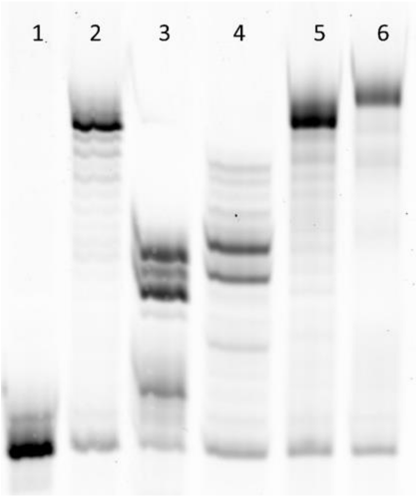

Figure S8. 20% denaturing PAGE analysis of PEX reactions. (1) RNA primer; (2) positive control with natural NTPs, **FAM_31_RNA**; (3) negative control in absence of **C^{ESQ}TP**; (4) negative control in absence of **A^{ESQ}TP**; (5) **FAM_31_RNA_4C^{ESQ}**; (6) **FAM_31RNA_4A^{ESQ}**.

Table S3. LC-MS data of RNA oligonucleotides prepared by PEX.

	Size (nt)	Sequence (5´-3´ direction)	Calc. mass (Da)	Found mass (Da)
**FAM_31_RNA**	31	CAUGGGCGGCAUGGGACUGAGCUCA UGCUAG	10566	10566
**FAM_31_RNA_4C^{ESQ}**	31	CAUGGGCGGCAUGGGA**C**^{ESQ}UGAG**C**^{ESQ}U**C**^{ESQ}A UG**C**^{ESQ}UAG	11274	11670
**FAM_31_RNA_4A^{ESQ}**	31	CAUGGGCGGCAUGGG**A**^{ESQ}CUG**A**^{ESQ}GCUC**A**^{ESQ}U GCU**A**^{ESQ}G	11274	11658

Reaction of ethoxy squarate-modified RNA (35_RNA_1C^{ESQ}) with amine-linked Cy5 molecule

Fluorescence measurements

Unlabelled natural RNA and **35_RNA_1C^{ESQ}** were prepared by *in vitro* transcription reaction described above. The reaction mixtures for sulfo-Cy-5-amine addition (10 µL) were prepared from purified transcribed product (5 µM) reconstituted in PBS buffer (pH 7.4) and sulfo-Cy-5-amine was added (2 mM). All samples were incubated on 37 °C overnight. The products were purified on NucAway Spin Columns and the fluorescence of the products was measured on a Fluoromax 4 spectrofluorimeter (**Figure S9-a**).

PAGE analysis

Unlabelled natural RNA and **35_RNA_1C^{ESQ}** were prepared by *in vitro* transcription reaction described above. The reaction mixtures for sulfo-Cy-5-amine addition (15 µL) were prepared from purified transcription product (9 µM) in TRIS-HCl buffer (40 mM, pH 7.9) (19 µL) and sulfo-Cy-5-amine (0.900 mM). All samples were incubated on 37 °C overnight. The products were purified on NucAway Spin Columns mixed with 2×PAGE stop solution without xylene cyanol and heated for 5 min at 95 °C. Samples were separated by 12.5% PAGE (acrylamide/bisacrylamide 19:1, 25% urea) under denaturing conditions (TBE 1×, 42 mA, 1 hour), and the gel was stained with Sybr Gold. Visualization was performed by fluorescence imaging using Typhoon FLA 9500, GE Healthcare (**Figure S9-b**).

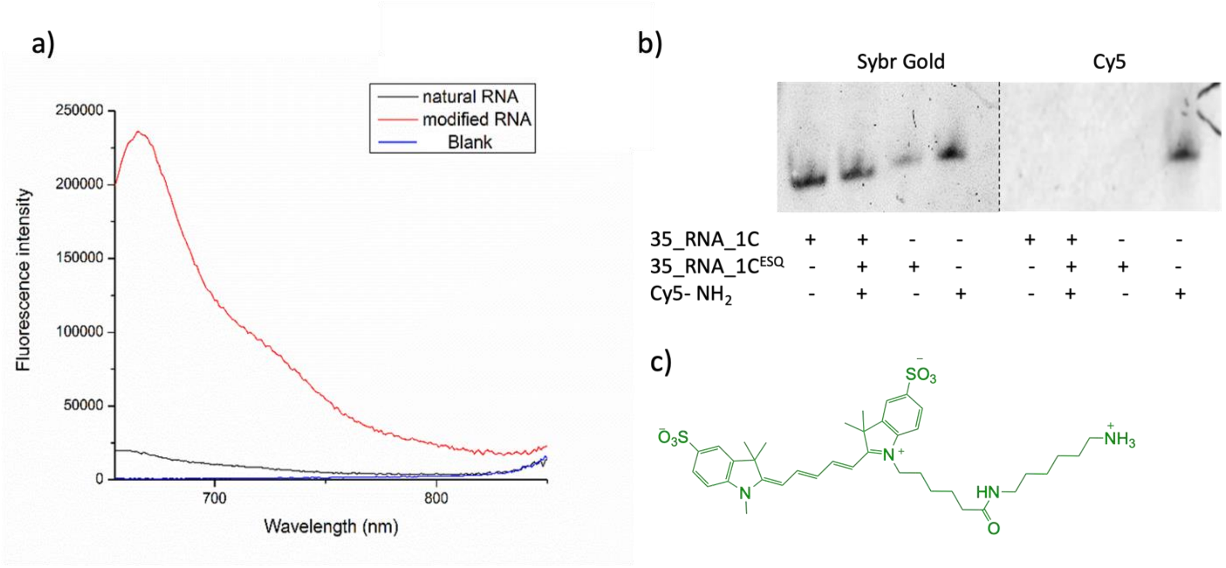

Figure S9. Conjugation of natural (**35_RNA_1C**) and ethoxy squarate modified **35_RNA_1C^{ESQ}** with sulfo-Cy-5-amine. a) Fluorescence measurements. Conditions: unlabeled natural RNA (Control line in black) or labeled **35_RNA_1C^{ESQ}_Cy5** (Reaction line in red), PBS buffer (pH 7.4), 37 °C, overnight. Fluorescence of the products was measured on spectrofluorimeter (water; λ_{ex}=646 nm, λ_{em}=662 nm). b) PAGE analysis: Tris HCl buffer (40 mM, pH 7.9), 37 °C, overnight. Excited with 473 nm for Sybr Gold and 635 nm for Cy5. c) Structure of sulfo-Cy-5-amine used in this reaction.

Reaction of ethoxy squarate-modified RNA (35_RNA_1C^{ESQ}, 35_RNA_1A^{ESQ} and 35_RNA_3C^{ESQ}) with various ratios of tripeptide (Ac-Lys-Ala-Ala)

Reaction mixtures (40 µL total volume) containing **35_RNA_1C^{ESQ}** or **35_RNA_3C^{ESQ}** (2 µM final concentration), tripeptide AcKAA (**P**; 500/5000/9000 equiv., **Figure S12b**) and buffer (100 mM borate pH 9 or 20 mM carbonate-bicarbonate buffer pH 9.5) were heated at 37 °C for 48 h. Crude reaction mixtures were purified using Monarch RNA cleanup kit and characterized by 20% denaturing PAGE (**Figure S10-11**).

a)

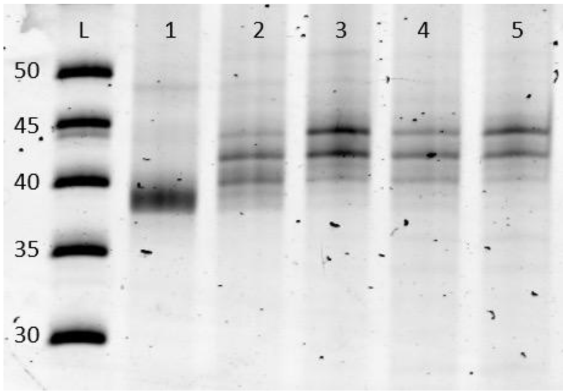

Figure S10. 20% denaturing PAGE of cross-linking reactions of **35_RNA_3C^{ESQ}** with different RNA/peptide ratio in 100 mM borate buffer pH 9 (lanes 2-3) and pH 10 (lanes 4-5). L: dsDNA ladder; Lane 1: **35_RNA_3C^{ESQ}**; Lanes 2 and 4: 500 equiv. of peptide; Lanes 3 and 5: 5000 equiv. of peptide.

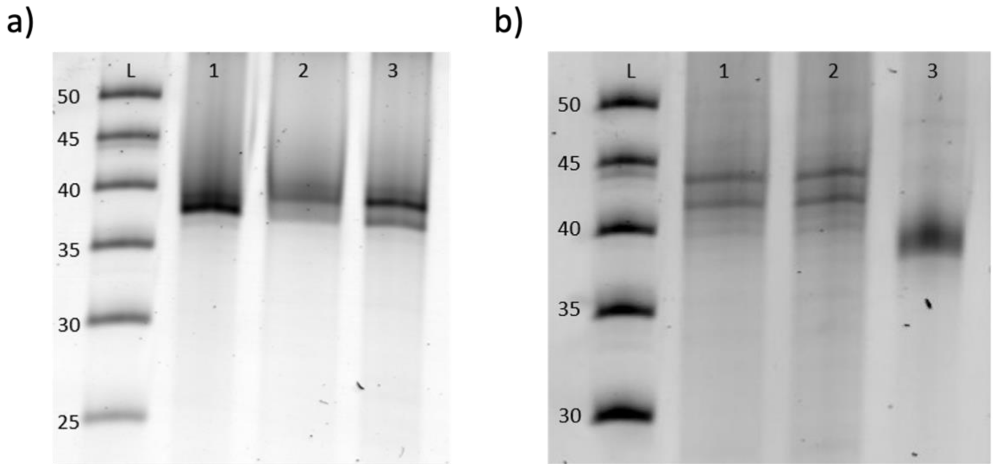

Figure S11. 20% dPAGE analysis of cross-linking reactions of a) **35_RNA_1C^{ESQ}** with 9000 equiv. of peptide in two different buffers. L: dsDNA ladder; Lane 1: **35_RNA_1C^{ESQ}**; Lane 2: 100 mM borate buffer pH 9; Lane 3: carbonate-bicarbonate buffer pH 9.5. b) **35_RNA_3C^{ESQ}** with 9000 equiv. of peptide in two different buffers. L: dsDNA ladder; Lane 1: 100 mM borate buffer pH 9; Lane 2: carbonate-bicarbonate buffer pH 9.5; Lane 3: **35_RNA_3C^{ESQ}**.

Reaction of ethoxy squarate-modified RNA (35_RNA_1A^{ESQ}) and chloroacetamide-modified RNA (35_RNA_1A^{CA}) with peptide (AcKAA) and Glutathione (GSH)

Reaction mixtures (25 µL total volume) containing **35_RNA_1A^{ESQ}** or **35_RNA_1A^{CA}** (2 µM final concentration), tripeptide AcKAA (10000 equiv.) or GSH (10000 equiv) in 100 mM borate buffer (pH 9) were incubated at 37 °C for 48 h. Crude reaction mixtures were purified using Monarch RNA cleanup kit and characterized by 20% denaturing PAGE (**Figure S12a**) and LC-MS (**Table S4**).

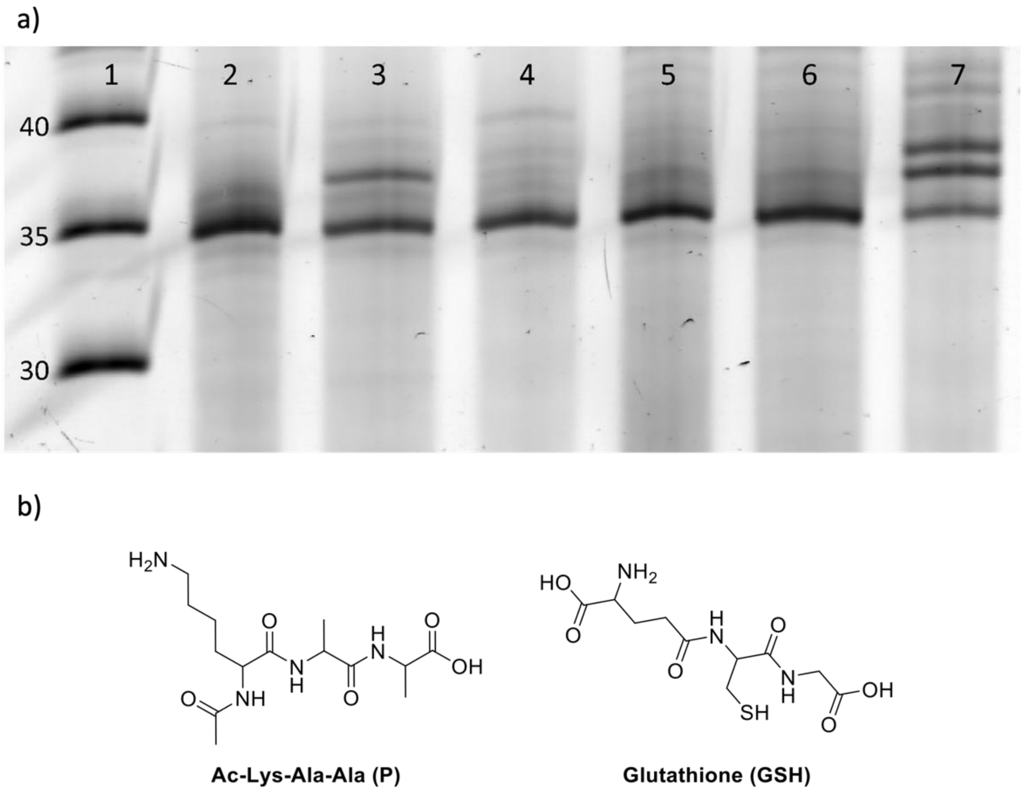

Figure S12. a) 20% denaturing PAGE of cross-linking reactions. (1) RNA ladder; (2) **35_RNA_1A$^{\mathbf{ESQ}}$**; (3) reaction of **35_RNA_1A$^{\mathbf{ESQ}}$** with peptide, **35_RNA_1A$^{\mathbf{ESQ\text{-}P}}$**; (4) reaction of **35_RNA_1A$^{\mathbf{ESQ}}$** with GSH, no product formation; (5) **35_RNA_1A$^{\mathbf{CA}}$**; (6) reaction of **35_RNA_1A$^{\mathbf{CA}}$** with peptide, no product formation; (7) reaction of **35_RNA_1A$^{\mathbf{CA}}$** with GSH, **35_RNA_1A$^{\mathbf{CA\text{-}GSH}}$**. b) structures of peptide (P) and Glutathione (GSH) used in the cross-linking reactions with modified RNAs.

Table S4. LC-MS data of RNA oligonucleotides prepared by transcription reaction using T7RNAP and products of modified RNA crosslinking with peptide and GSH.

	Size (nt)	Sequence (5´-3´ direction)	Calc. mass (Da)	Found mass (Da)
**35_RNA_1C^{ESQ}**	35	pppGGGAGGGA**C**^{ESQ}TGTGAGTGGAGATTGTA GGATTGAGG	11954	11978
**35_RNA_1C^{ESQ-P}**	35	pppGGGAGGGA**C**$^{ESQ-P}$TGTGAGTGGAGATTG TAGGATTGAGG	12238	12279
**35_RNA_3C^{ESQ}**	35	pppGGGAGGAT**C**^{ESQ}AGTA**C**^{ESQ}AGAGGTAT G**C**^{ESQ}TGGGATAGGGA	12275	12314
**35_RNA_3C^{ESQ-P}**	35	pppGGGAGGAT**C**$^{ESQ-P}$AGTA**C**$^{ESQ-P}$ AGAGGTATG**C**$^{ESQ-P}$TGGGATAGGGA	13127	13150
**35_RNA_1A^{ESQ}**	35	pppGGGCCCCUA**A**^{ESQ}UUGUCUCUCUCUUCUC UGCUGUUUCC	11313	11339
**35_RNA_1A^{CA}**	35	PppGGGCCCCUA**A**^{CA}UUGUCUCUCUCUUCUCU GCUGUUUCC	11268	11324
**35_RNA_1A^{ESQ-P}**	35	pppGGGCCCCUA**A**$^{ESQ-P}$UUGUCUCUCUCUUCUCU GCUGUUUCC	11597	11595
**35_RNA_1A^{CA-GSH}**	35	pppGGGCCCCUA**A**$^{CA-GSH}$UUGUCUCUCUCUUCU CUGCUGUUUCC	11539	11572
**35_RNA_3A^{ESQ}**	35	pppGGGCCCGUA**A**^{ESQ}UGUUA**A**^{ESQ}CUUGCUC UUA**A**^{ESQ}UCGUCUCUCGC	11793	11789

Cross-linking of ESQ-modified RNAs with proteins

Note

Conversions of cross-linking reactions were determined from the gel using ImageJ Quantificator.

Cross-linking of 36_vRNA_1gC^{ESQ}, 36_vRNA_3fC^{ESQ} with T7 RNA polymerase

Radioactively labelled natural RNA and **36_vRNA_1gC^{ESQ}**, **36_vRNA_3fC^{ESQ}** (with one and three modifications respectively) were prepared by *in vitro* transcription as described above. Natural or modified RNA (1µM) was incubated with 2U µL^{-1} of T7 RNA polymerase at 37 °C. Reaction mixture contained $MgCl_2$ (4.80 mM), dithiothreitol (DTT; 12 mM), Triton X-100 (0.120%), dimethyl sulfoxide (DMSO; 5%) and 1× transcription buffer B (40 mM Tris-HCl (pH 7.9), 6 mM $MgCl_2$, 10 mM DTT, 10 mM NaCl, 2mM betain) and DEPC water (total reaction volume 25 µL). After 30 min 5 µL of the reaction mixture was separated by 5-10% native PAGE (acrylamide/bisacrylamide 37.5:1; 4 °C, 200 V, 0.250 M Tris, 0.192 M glycine,). The rest of the reaction was incubated up to 2 hours at 37 °C, then diluted with 2×VPS loading buffer, denatured 10 min at 95 °C prior to loading and analysed by 5-10% SDS denaturing PAGE (acrylamide/bisacrylamide 37.5:1; 0.250 M Tris, 0.192 M glycine, 0.100% SDS) at room temperature (230 V, 70 min). Visualization was performed by phosphoimaging (**Figure S13**).

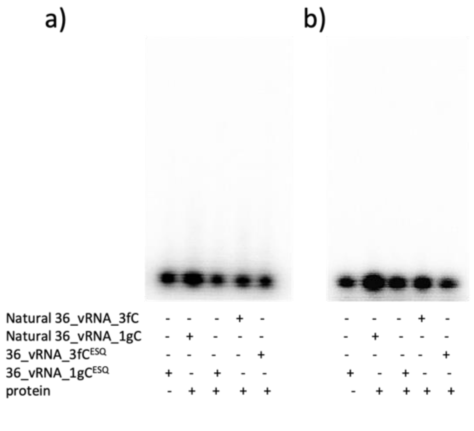

Figure S13. Phosphor image of 10% SDS analysis of control experiment with natural or modified RNA and T7 RNA polymerase. Conditions: RNA (1 µM), 37 °C, 30 min for EMSA (a) and 2h for SDS (b).

Cross-linking of 36_vRNA_1gC^{ESQ} with Bovine Serum Albumin (BSA) and Single Strand Binding Protein (SSB)

Radioactively labelled natural RNA and **36_vRNA_1gC^{ESQ}** were prepared by *in vitro* transcription as described above. Natural or modified RNA (1µM) was incubated with 20 µM protein (BSA resp. SSB) in 10× binding buffer D (100 mM Tris pH. 8.0, 20 mM $MgCl_2$, 100 mM KCl, 12 mM βME; 2µL), 50% glycerol (2 µL) and DEPC water (total reaction volume 20 µL) at 37 °C. After 1 hour 5 µL of the reaction mixture was separated by 5-10% native PAGE (acrylamide/bisacrylamide 37.5:1; 4 °C, 200V, 0.250 M Tris, 0.192 M glycine,). The rest of the reaction was incubated overnight at 37°C, then diluted with 2× VPS loading buffer, denatured 10 min at 95 °C prior to loading and analysed by 5-10% SDS denaturing PAGE (acrylamide/bisacrylamide 37.5:1; 0.250 M Tris, 0.192 M glycine, 0.100% SDS) at room temperature (230 V, 70 min). Visualization was performed by phosphoimaging (**Figure S14**)

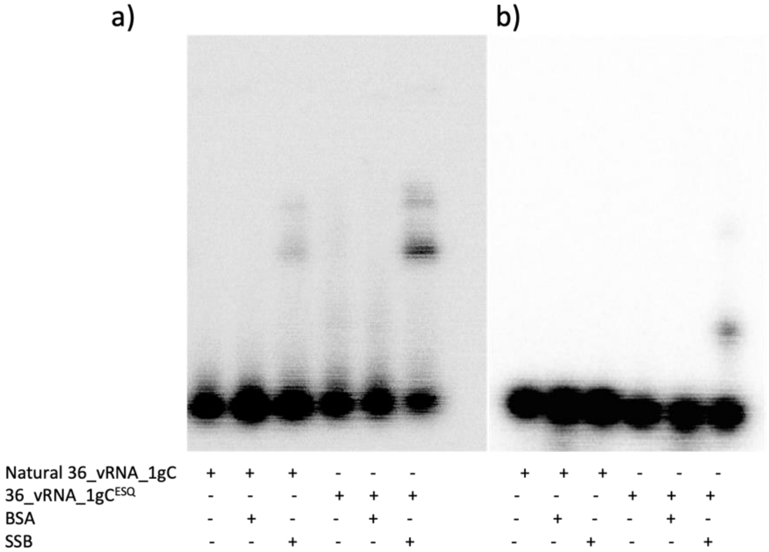

Figure S14. Phosphor image of SDS analysis of control experiment with natural or modified RNA and various RNA non-binding recombinant proteins (20 equiv. of BSA or SSB to RNA): 10% SDS gel. Conditions: 37 °C, 1h for EMSA (a) and 24h for SDS (b).

Cross-linking of 36_vRNA_1gC^{ESQ}, 36_vRNA_3fC^{ESQ} with Nonstructural Protein NS5 of Japanese Encephalitis Virus (JEV) and Yellow Fever Virus (YFV)

Radioactively labelled natural RNA and **36_vRNA_1gC^{ESQ}, 36_vRNA_3fC^{ESQ}** (with one and three modifications respectively) were prepared by *in vitro* transcription as described above. Natural or modified RNA (0.500 µM) was incubated with 2 µM of NS5 protein (JEV resp. YFV) in 10× binding buffer E (50 mM Tris-HCl pH. 7.4, 100 mM DTT, 5% Triton X-100, 10% glycerol; 2µL), 10 mM $MnCl_2$ (2 µL), 10 mM $MgCl_2$ (2 µL), 50% glycerol (2 µL) and DEPC water (total reaction volume 20 µL) at 34 °C. After 1 hour 3 µL of the reaction mixture was separated by 5-7% native PAGE (acrylamide/bisacrylamide 37.5:1; 4 °C, 200 V, 1× Tris-glycine). The rest of the reaction was incubated 48 h at 34 °C, then diluted with 2× VPS loading buffer, denatured 10 min at 95 °C prior to loading and analysed by 5-10% SDS denaturing PAGE (acrylamide/bisacrylamide 37.5:1; 0.250 M Tris, 0.192 M glycine, 0.100% SDS) at room temperature (230 V, 70 min). Visualization was performed by phosphoimaging (**Figure 15**).

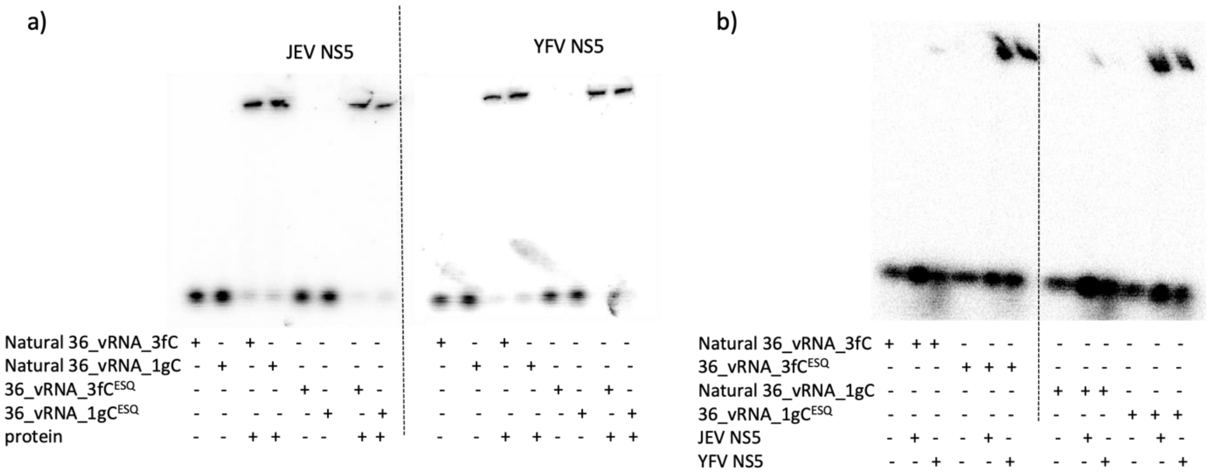

Figure S15. Phosphor image of 7% protein gel from crosslinking reaction between JEV and YFV NS5 RdRp and RNA with 3 or 1 modification. Conditions: 4 equiv. of protein to RNA (0.5 µM), 34 °C, 1h for EMSA (a) and 48h for SDS (b).

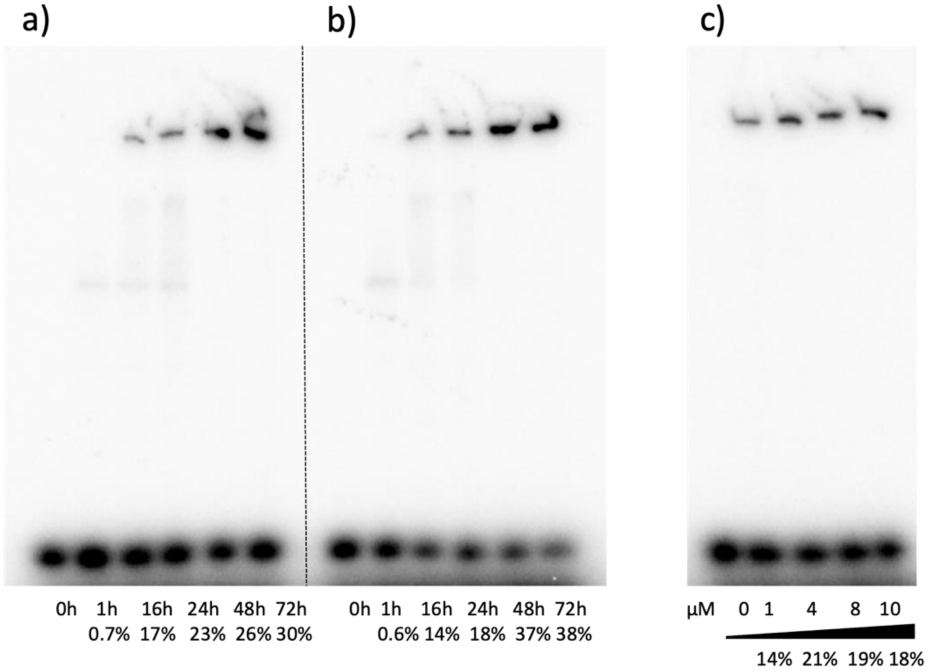

Figure S16. Effect of reaction time on cross-link formation between JEV NS5 RdRp and a) **36_vRNA_1gC^{ESQ}** b) **36_vRNA_3fC^{ESQ}**; phosphor image of 10% SDS gel. Conditions: 4 equiv. of protein to RNA, BB, 34 °C. c) Effect of the RNA/protein ratio on cross-link formation between JEV NS5 RdRp; 10 % SDS gel. Conditions: JEV NS5 RdRp (0, 1, 4, 8, 10 µM), **36_vRNA_1gC^{ESQ}** (1 µM), 34 °C/48h.

Cross-linking of 36_vRNA_1gC^{ESQ}, 36_vRNA_3fC^{ESQ} with SARS-CoV-2 RdRp

Radioactively labelled natural RNA and **36_vRNA_1gC^{ESQ}, 36_vRNA_3fC^{ESQ}** (with one and three modifications respectively) were prepared by *in vitro* transcription as described above. Natural or modified RNA (1 µM) was incubated with 8 µM SARS-CoV-2 RdRp protein (complex of 2 µM SC nsp12 and 6 µM nsp7/8 proteins) in 10× binding buffer D (100 mM Tris-HCl pH. 8, 20 mM $MgCl_2$, 100 mM KCl 12 mM βME; 2µL), 50% glycerol (2 µL) and DEPC water (total reaction volume 20 µL) at 30 °C. After 1 hour 3 µL of the reaction mixture was separated by 10% native PAGE (acrylamide/bisacrylamide 37.5:1; 4 °C, 200 V, 1× Tris-glycine). The rest of the reaction was incubated overnight at 30 °C, then diluted with 2× VPS loading buffer, denatured 10 min at 95 °C prior to loading and analysed by 5-10% SDS denaturing PAGE (acrylamide/bisacrylamide 37.5:1; 0.250 M Tris, 0.192 M glycine, 0.100% SDS) at room temperature (230 V, 70 min). Visualization was performed by phosphoimaging (**Figure S17**).

Cross-linking of 36_vRNA_1gC^{ESQ}, 36_vRNA_3fC^{ESQ} with SARS-CoV-2 nucleoprotein

Radioactively labelled natural RNA and **36_vRNA_1gC^{ESQ}, 36_vRNA_3fC^{ESQ}** (with one and three modifications) were prepared by *in vitro* transcription as described above. Natural or modified RNA (1 µM) was incubated with 8 µM SARS-CoV-2 nucleoprotein in 10× binding buffer D (100 mM Tris-HCl pH. 8, 20 mM $MgCl_2$, 100 mM KCl 12 mM βME; 2µL), 50% glycerol (2 µL) and DEPC water (total reaction volume 20 µL) at 30 °C. After 1 hour 3 µL of the reaction mixture was separated by 10% native PAGE (acrylamide/bisacrylamide 37.5:1; 4 °C, 200 V, 1× Tris-glycine). The rest of the reaction was incubated overnight at 30 °C, then diluted with 2× VPS loading buffer, denatured 10 min at 95 °C prior to loading and analysed by 5-10% SDS denaturing PAGE (acrylamide/bisacrylamide 37.5:1; 0.250 M Tris, 0.192 M glycine, 0.100% SDS) at room temperature (230 V, 70 min). Visualization was performed by phosphoimaging (**Figure S17**).

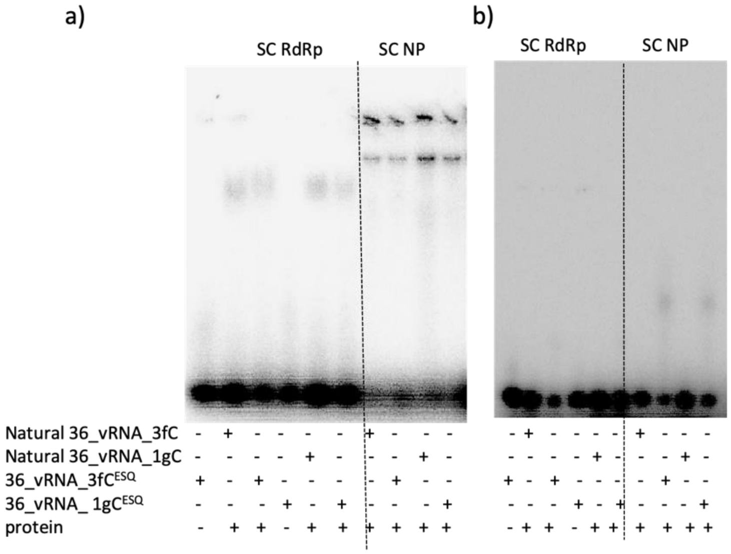

Figure S17. Phosphor image of SDS analysis of control experiment with natural or modified RNA and SARS CoV2 RdRp and SARS CoV2 nukleoprotein (8 equiv. of protein to 1 µM RNA): 10% SDS gel. Conditions: 30 °C, 1h for EMSA (a) and over night for SDS (b).

Cross-linking of 36_vRNA_1gC^{ESQ} with HIV-rt

Radioactively labelled natural RNA and **36_vRNA_1gC^{ESQ}** (with one modification) was prepared by *in vitro* transcription as described above. Natural or modified RNA (0.500 µM) was incubated with 1 µM HIV-rt in buffer B (40 mM Tris-HCl (pH 7.9), 6 mM $MgCl_2$, 10 mM DTT, 10 mM NaCl, 2mM betain), 50% glycerol (2 µL) and DEPC water (total reaction volume 20 µL) at 37 °C. After 1 hour 3 µL of the reaction mixture was separated by 10% native PAGE (acrylamide/bisacrylamide 37.5:1; 4 °C, 200 V, 1× Tris-glycine). The rest of the reaction was incubated overnight at 37 °C, then diluted with 2× VPS loading buffer, denatured 10 min at 95 °C prior to loading and analysed by 5-10% SDS denaturing PAGE (acrylamide/bisacrylamide 37.5:1; 0.250 M Tris, 0.192 M glycine, 0.100% SDS) at room temperature (230 V, 70 min). Visualization was performed by phosphoimaging (**Figure S18**).

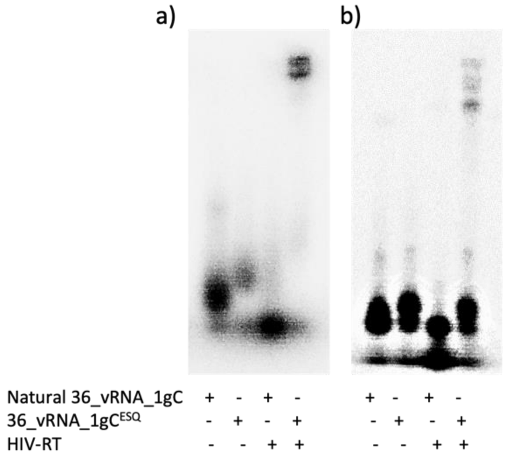

Figure S18. Phosphor image of SDS analysis of control experiment with natural or modified RNA and HIV reverse transcriptase (2 equiv. of protein to 0.5 µM RNA): 10% SDS gel. Conditions: 37 °C, 1h for EMSA (a) and over night for SDS (b).

Table S5. RNA-protein cross-link conversions between **36_RNA_3fC^{ESQ}** and recombinant proteins.

Protein	Molecular weight [kDa]	RNA binding	No. of Lys residues	Conversion %
T7 RNAP	99	no	66	0%
JEV NS5	103.3	yes	58	41%
YFV NS5	103.3	yes	60	42%
SC RdRp	62.2	yes	72	traces
SC NP	45.6	yes	31	traces

Note: Conversions were determined from the gel using ImageJ Quantificator.

1.2.4 Gel-based polymerase assays in vitro

JEV and YFV RdRp mediated RNA extension assay[4]

The polymerase activity of NS5 proteins was determined in a PEX reaction using fluorescently labelled RNA templates (**Table S2**). The reaction mixture (20 µl) contained reaction buffer (5 mM Tris-HCl pH. 7.4, 10 mM DTT, 0.100% Triton X-100, 1% glycerol, 1 mM $MnCl_2$, 1 mM $MgCl_2$), 10 µM NTPs, 200 nM template and 800 nM NS5 proteins. In the positive control, all natural NTPs were used and in the modified version 10 µM **C^{ESQ}TP** instead of CTP was used. The reactions were incubated for 1 h and overnight respectively, at 34 °C. Then the reactions were incubated with Proteinase K (1U) 30 min at room temperature. Reactions were stopped by adding of 2× PAGE stop solution and denatured at 95 °C for 10 min prior to loading. Samples were separated by 12.5% PAGE (acrylamide/bisacrylamide 19:1, 25% urea) under denaturing conditions (TBE 1×, 42 mA, 1 hour). Visualization was performed by fluorescence imaging using Typhoon FLA 9500, GE Healthcare (**Figure S19-a/b**). The same reaction mixture was analysed on the SDS protein gel. The gel was stained with PageBlue^{TM} protein staining solution (**Figure S19-c**).

Immunodetection of 46_RNA_HP^{C4_JEVNS5} conjugate

Natural 46_RNA_HP and **46_RNA_HP^{C4}** were prepared by *in vitro* extension assay as described above. The reaction mixtures were diluted with 2× VPS loading buffer, denatured 10 min at 95 °C prior to loading and electrophoresed on 5-10% SDS denaturing PAGE (acrylamide/bisacrylamide 37.5:1; 0.250 M Tris, 0.192 M glycine, 0.100% SDS) at room temperature (230 V, 70 min). Visualization of FAM labelled cross-linked product was performed by fluorescence imaging using Typhoon FLA 9500, GE Healthcare (**Figure S19-d**). The gel was blotted (dry; 0.250 M Tris, 0.192 M glycine, 10% methanol, 0.100% SDS; 12 V/2 hours at r.t.) to polyvinylidene fluoride Immobilon-P transfer membrane (Millipore). The membrane was washed with TBS (2.50 mM Tris, 15 mM NaCl, 0.200 mM KCl, pH 7.4), blocked with 5% non-fat milk (in T-TBS; 2.50 mM Tris, 15 mM NaCl, 0.200 mM KCl, 0.500% Tween-20, pH 7.4) for 30 min and afterwards washed with T-TBS (20 ml, 10 min). The membrane was incubated with JEV NS5 polyclonal antibody (rabbit/IgG; Invitrogen) at dilution 1:1000 in 5% non-fat milk in T-TBS at 4 °C overnight. The unbound antibodies were washed out with T-TBS (20 ml, 3 × 5 min). Next, the membrane was incubated with anti-rabbit IgG HRP linked secondary antibody (Invitrogen) at 1:1000 dilution in 5% non-fat milk in T-TBS at 25 °C for 1 hour. The access of antibodies was removed by washing with T-TBS (20 ml, 3 × 5 min) and TBS (20 ml, 10 min). The membrane was incubated with SuperSignal West Femto Chemiluminescent Substrate (Thermo Scientific) for 1 min. Chemiluminescence was measured on an ImageQuant LAS 4000 Mini luminescence analyser (GE Healthcare; **Figure S19-e**).

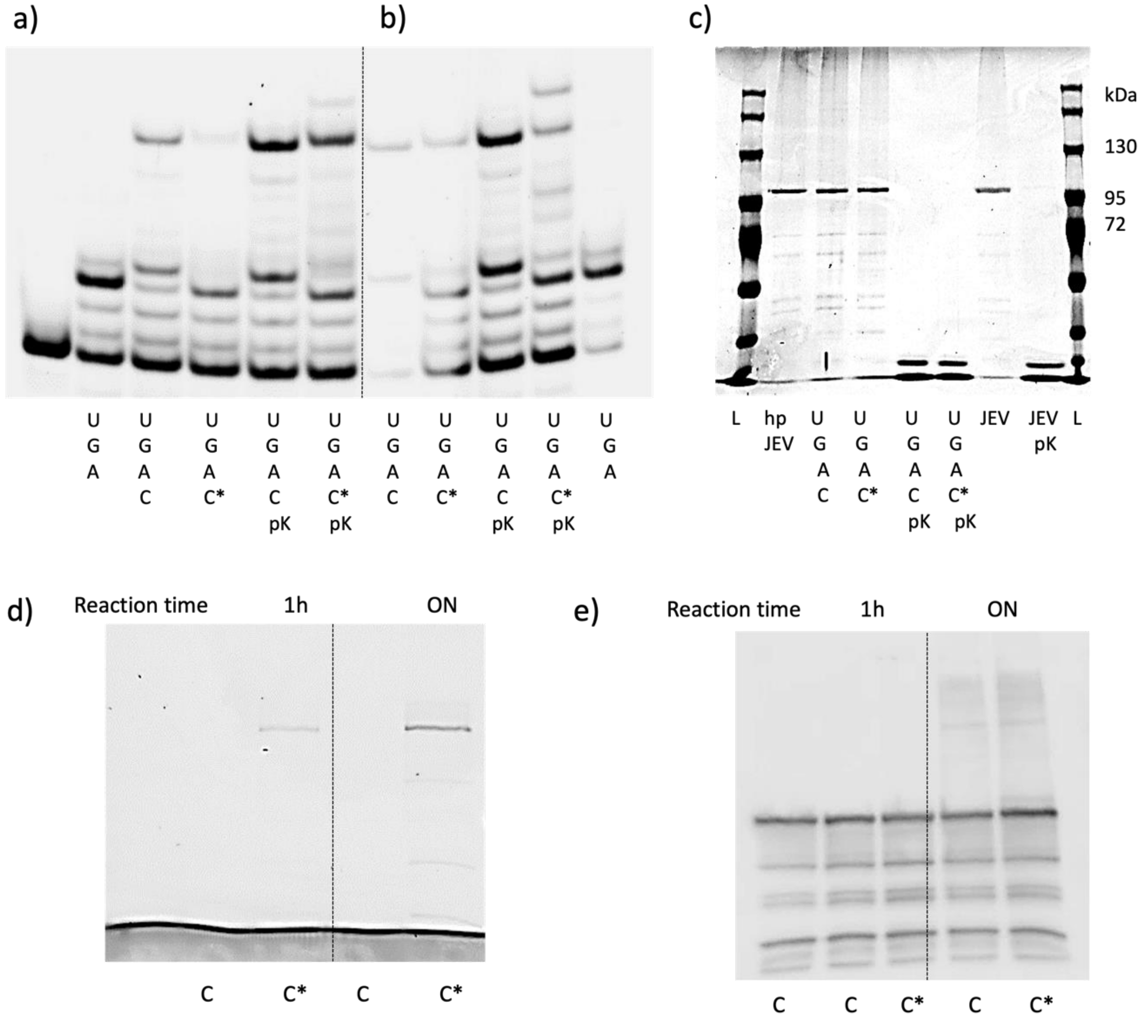

Figure S19. RdRp polymerase assay. a) Reaction time 1 hour b) reaction time overnight. Lane 1: template; C*: modified ($\mathbf{C}^{\mathbf{ESQ}}$); pk: Proteinase K treatment. c) PageBlue^{TM} protein staining of the SDS gel analysis of JEV RdRp assay (hp = template, JEV = protein, pK = Proteinase K, L = ladder). d) 5-10% SDS denaturing PAGE, FAM scan. Lane C: positive control (natural NTPs); lane C*: modified ($\mathbf{C}^{\mathbf{ESQ}}$, GTP, ATP, UTP). e) Western blot of the same gel. Lane C: positive control (natural NTPs); lane C*: modified ($\mathbf{C}^{\mathbf{ESQ}}$, GTP, ATP, UTP).

SARS-CoV-2 RdRp mediated RNA extension assay[5]

The polymerase activity of SARS-CoV-2 RdRp was determined in a PEX reaction using fluorescently labelled RNA templates (**Table S2**). The reaction mixture (20 µl) contained reaction buffer (10 mM Tris-HCl (pH. 8.0), 2 mM $MgCl_2$, 10 mM KCl, 1 mM βME), 10 µM NTPs, 0.500 µM template, 1 µM nsp12 and 3 µM nsp7/8 proteins. In the positive control, all natural NTPs were used and in the modified version 10 µM **C^{ESQ}TP** instead of CTP was used. The reactions were incubated for 1 h at 30 °C. Reactions were stopped by adding of 2× PAGE stop solution and denatured at 95 °C for 10 min prior to loading. Samples were separated by 12.5% PAGE (acrylamide/bisacrylamide 19:1, 25% urea) under denaturing conditions (TBE 1×, 42 mA, 1 hour). Visualization was performed by fluorescence imaging using Typhoon FLA 9500, GE Healthcare (**Figure S20**). For MALDI TOF analysis, the reaction mixture was desalted with Bio-Spin6/Biorad columns (buffer was exchanged for water according to supplied protocol; **Figure S29-30**).

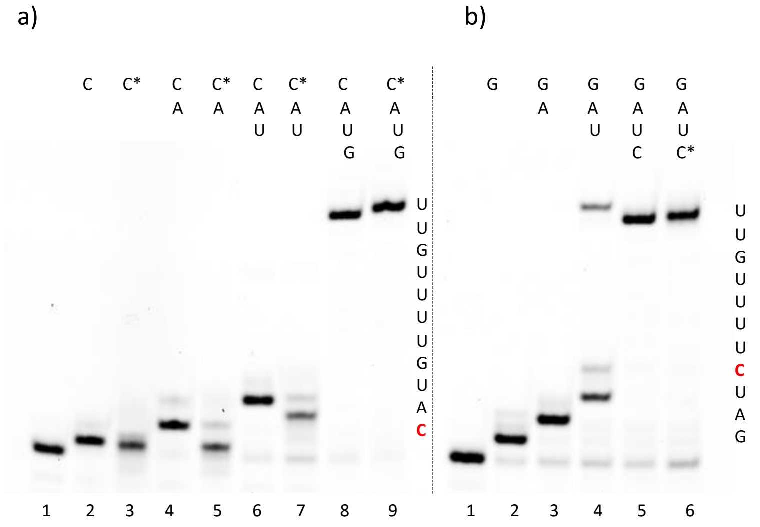

Figure S20. RNA extension in SARS-CoV-2 RdRp polymerase assay a) Template 29_RNA_tSC^{C1} (lane 1). Lanes 2-7: control reactions in the presence of indicated NTPs; lane 8: positive control (natural NTPs); lane 9: modified (**C^{ESQ}**, GTP, ATP, UTP). b) Template 29_RNA_tSC^{C4} (lane 1). Lanes 2-4: control reactions in the presence of indicated NTPs; lane 5: positive control (natural NTPs); lane 6: modified (**C^{ESQ}**, GTP, ATP, UTP).

1.2.5 Characterization of individual ethoxy squarate-modified RNAs and their cross-linking products with peptide and individual proteins by mass spectrometry

MALDI-TOF analysis of ethoxy squarate-modified RNAs

Ethoxy squarate modified RNAs were prepared by *In vitro* transcription reactions described above. After DNAse I treatment and spin-column purification, the products were freeze-dried, then redissolved in the water and analysed by MALDI-TOF mass spectrometry (**Figure S21-29**).

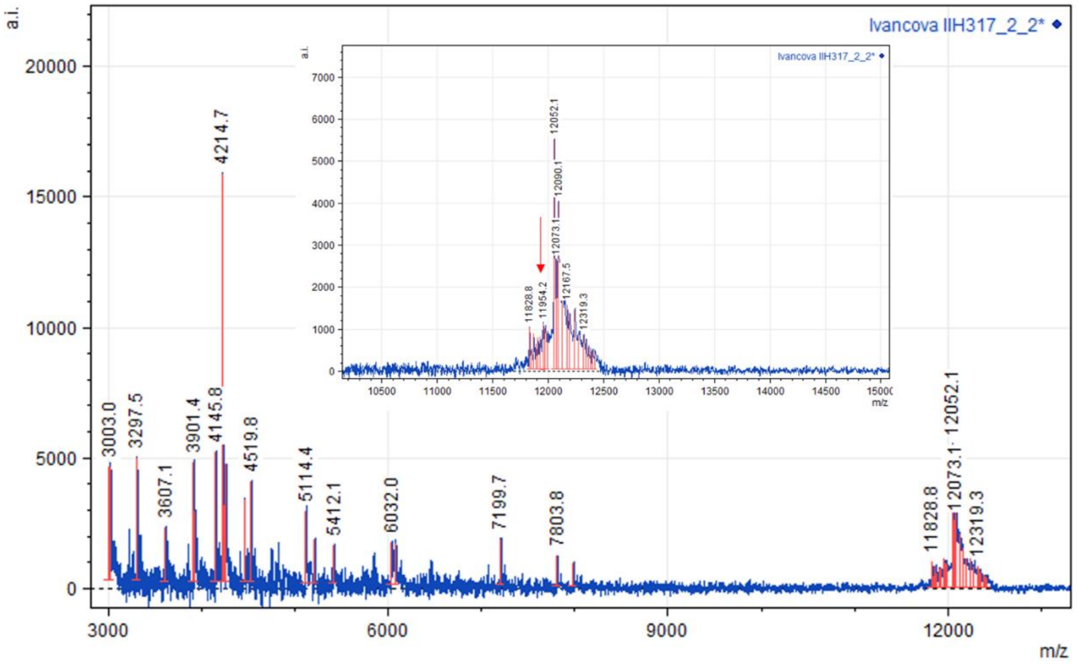

Figure S21. MALDI-TOF MS spectrum of **35_RNA_1C^{ESQ}** after transcription reaction in commercial T7 RNAP reaction buffer. M (calc.) = 11954 Da, M (found) = 11954.2 Da (red dart). The peak at *m/z* = 12052.1 can be assigned to the **35_RNA_1C^{ESQ}** reacted with spermidine (M = 145.25 Da) from reaction buffer.

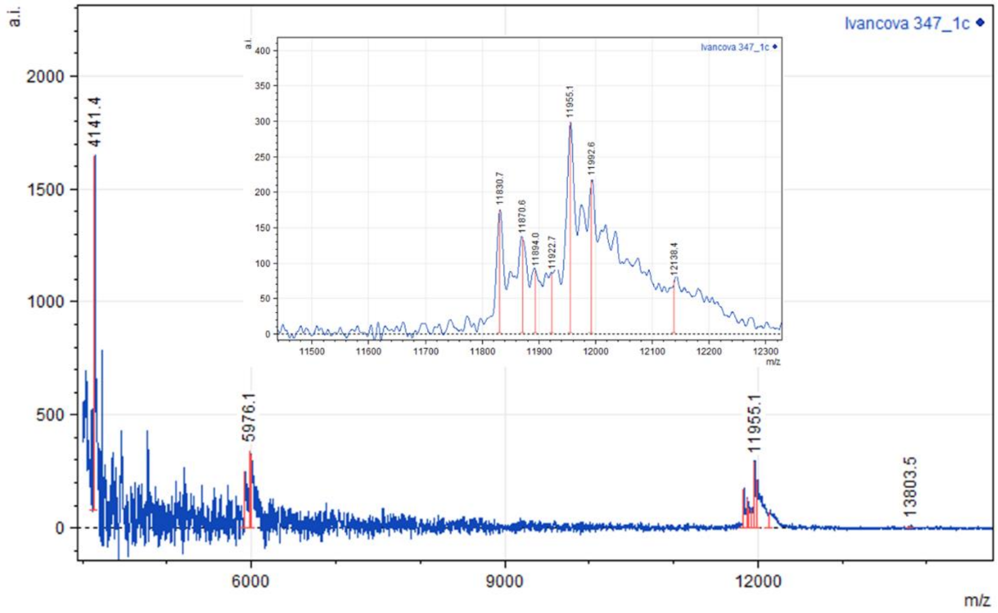

Figure S22. MALDI-TOF MS spectrum of **35_RNA_1C$^{\mathbf{ESQ}}$**. M (calc.) = 11954 Da, M (found) = 11955.1 Da [M+H]+.

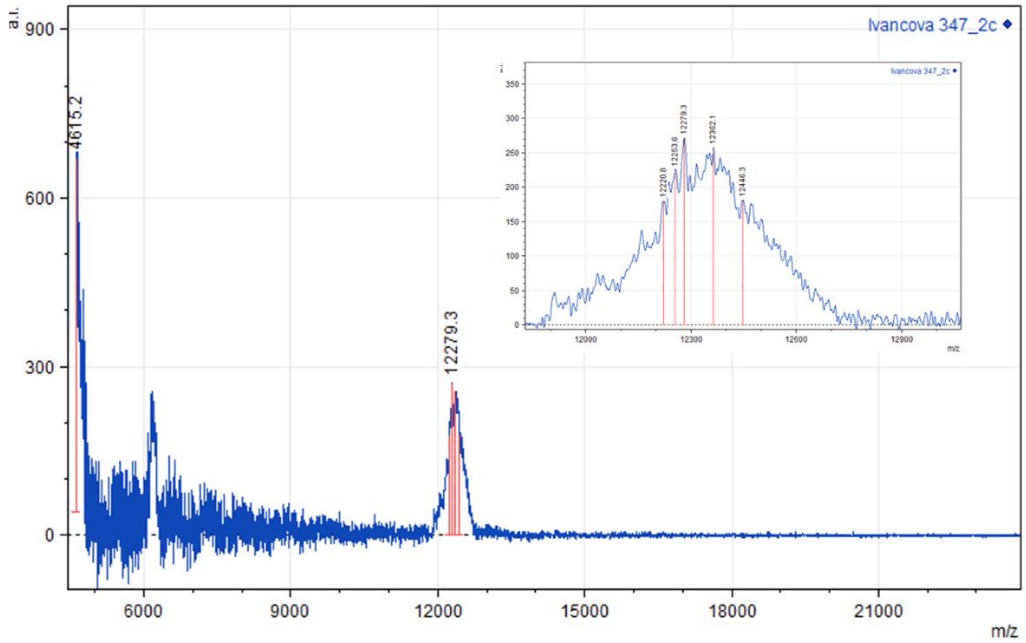

Figure S23. MALDI-TOF MS spectrum of **35_RNA_3C$^{\mathbf{ESQ}}$**. M (calc.) = 12277 Da, M (found) = 12279.3 Da $[M+2H]^+$.

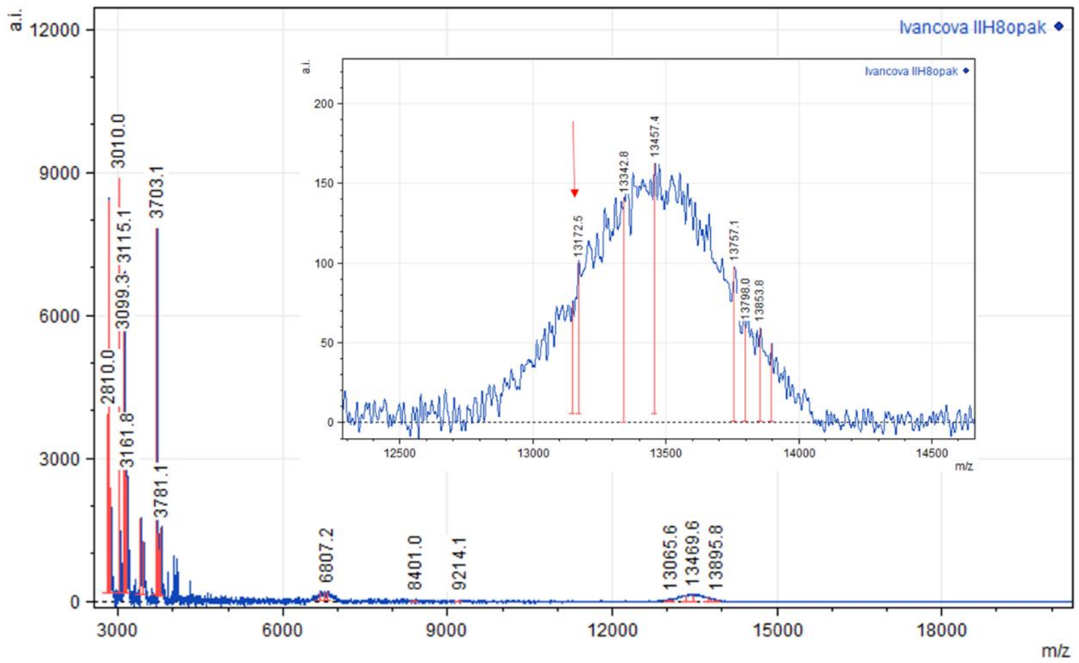

Figure S24. MALDI-TOF MS spectrum of **36_vRNA_8C$^{\mathbf{ESQ}}$**. M (calc.) = 13150.3 Da, M (found) = 13172.5 $[M+Na]^+$. The peak at *m/z* = 13457.4 Da can be assigned to the $[M+UMP]^+$.

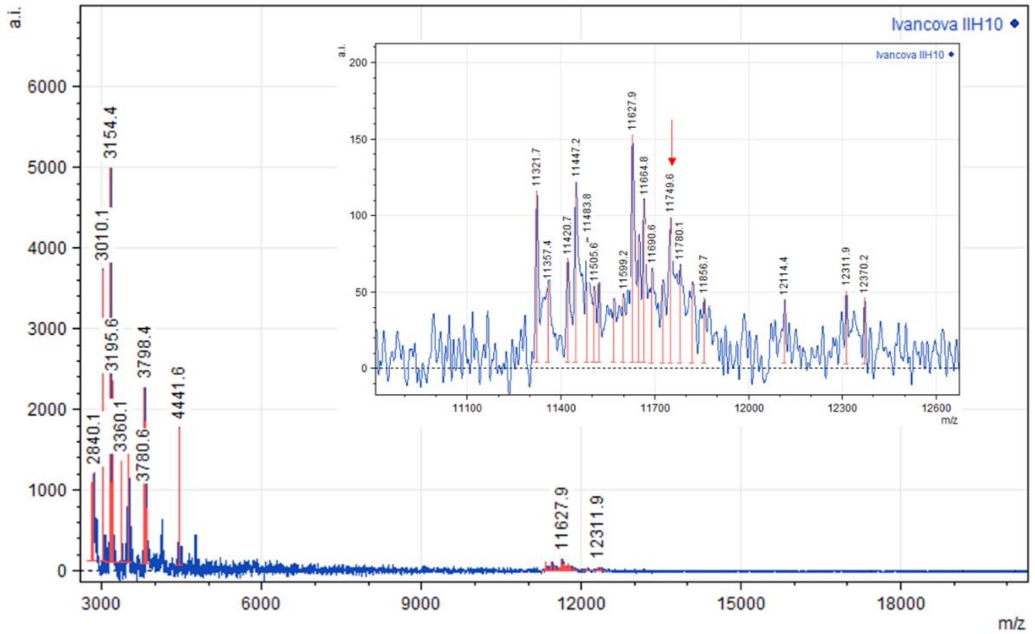

Figure S25. MALDI-TOF MS spectrum of **36_vRNA_1eC$^{\mathbf{ESQ}}$**. M (calc.) = 11748 Da, M (found) = 11749.6 Da $[M+1]^+$.

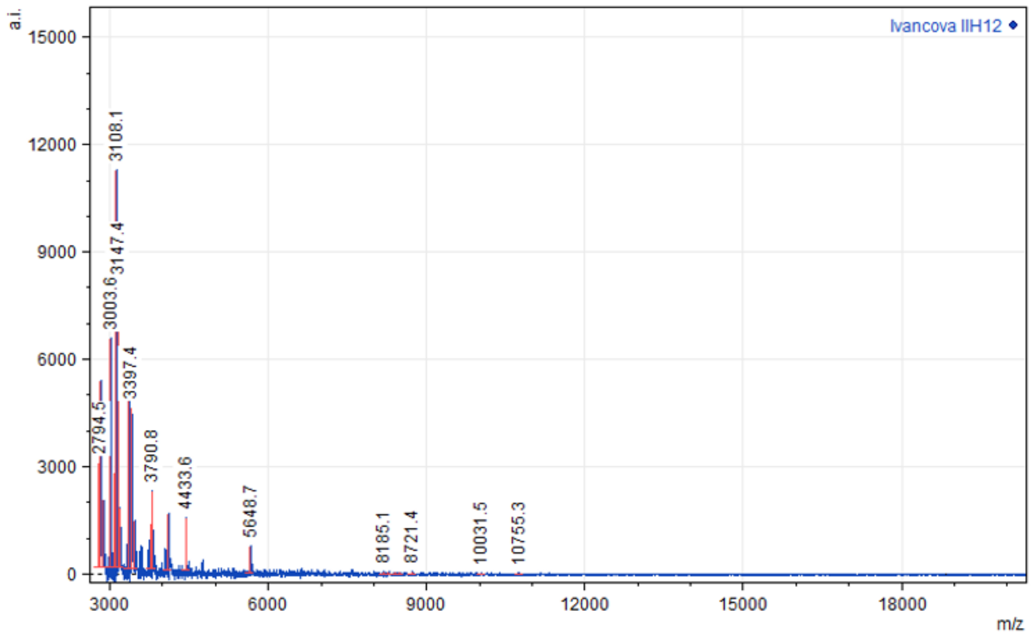

Figure S26. MALDI-TOF MS spectrum of **36_vRNA_3fC^{ESQ}** M (calc.) = 12354.5 Da, M not found.

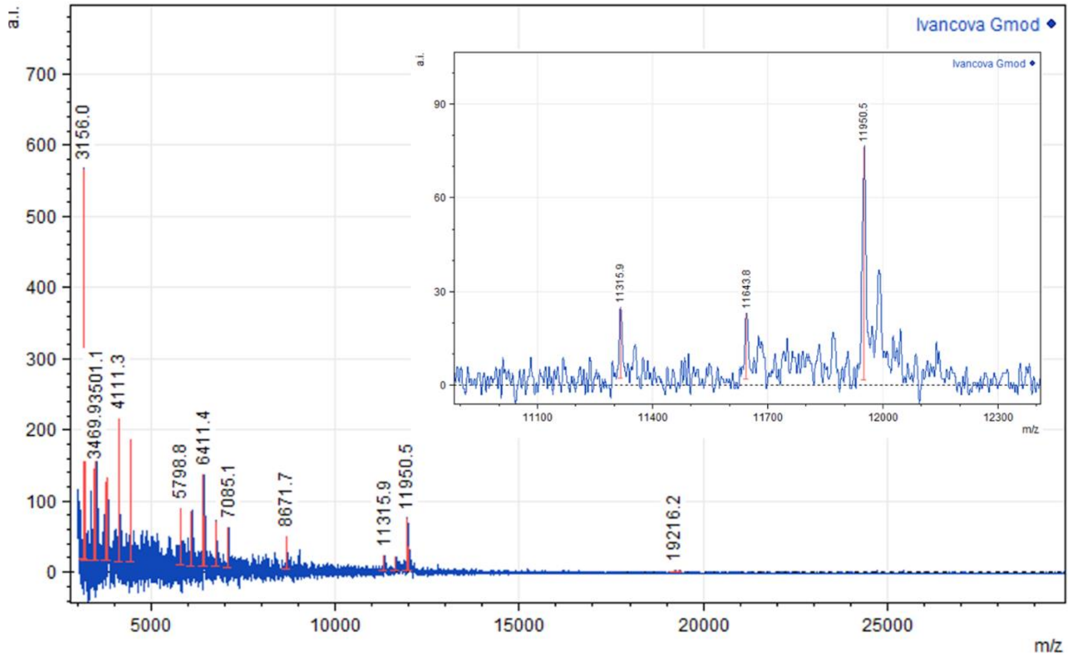

Figure S27. MALDI-TOF MS spectrum of **36_vRNA_1gC^{ESQ}**. M (calc.) = 12080 Da, M (found) = 11950.5 Da $[M-U-H_2O]^+$.[6]

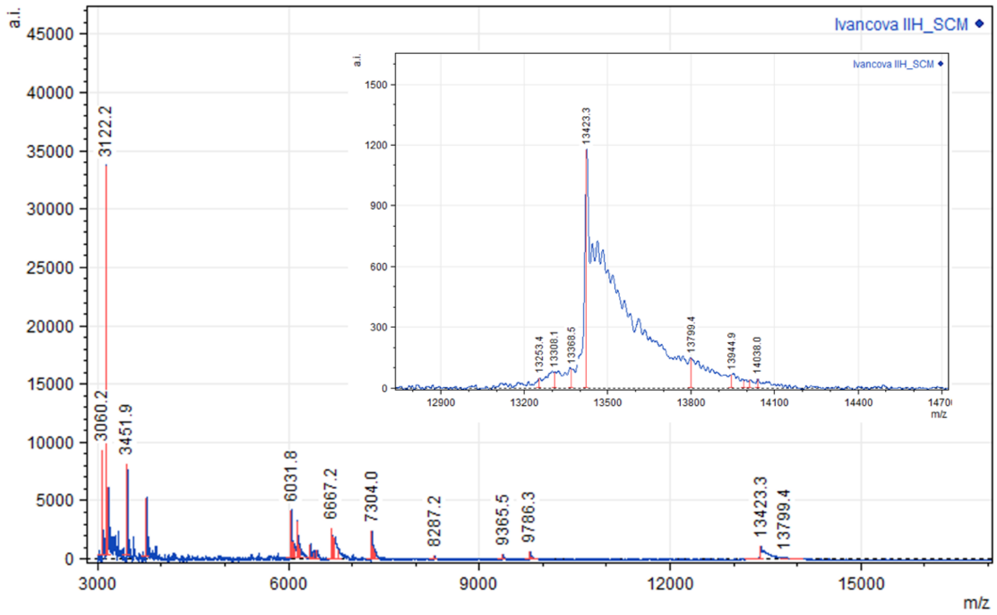

Figure S28. MALDI-TOF MS spectrum of **40_RNA_SC^{C1}**. M (calc.) = 13451.1 Da, M (found) = 13423.3 Da [M- CH_2CH_3 - H]$^+$.

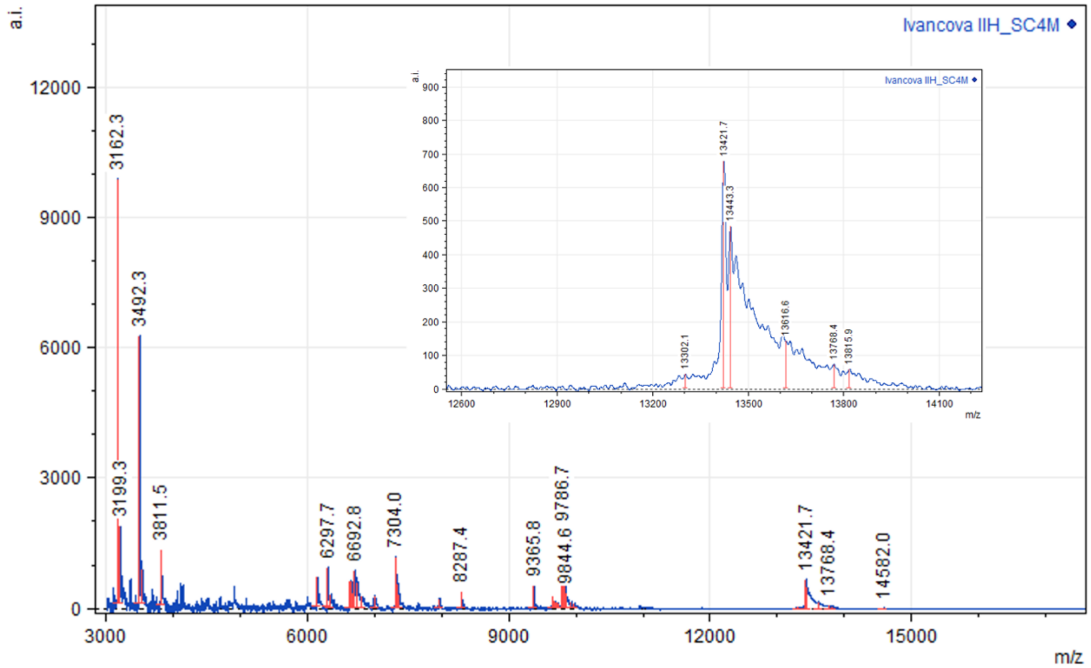

Figure S29. MALDI-TOF MS spectrum of **40_RNA_SC^{C4}**. M (calc.) = 13451.1 Da, M (found) = 13421.7 Da [M- CH_2CH_3]$^+$.

LC-MS characterization of ESQ-modified RNAs and their corresponding conjugates with peptide and GSH

Mass spectra of oligonucleotides and their conjugates with peptide or GSH were measured on Agilent 1290 Infinity II Bio system with DAD detector and mass spectrometer MSD XT. LC-MS analyses were carried out using bioZen 1.7 µm Oligo, 2.1x150 mm column (Kinetex) using mobile phase A (15 mM Et_3N, 300 mM HFIP in H_2O) and mobile phase B (15 mM Et_3N, 300 mM HFIP in MeOH) by 30 min gradient from 5% B to 50% B. Deconvolutions of LC-MS spectra were carried out using UniDec program[7] (**Figure S31-S43**).

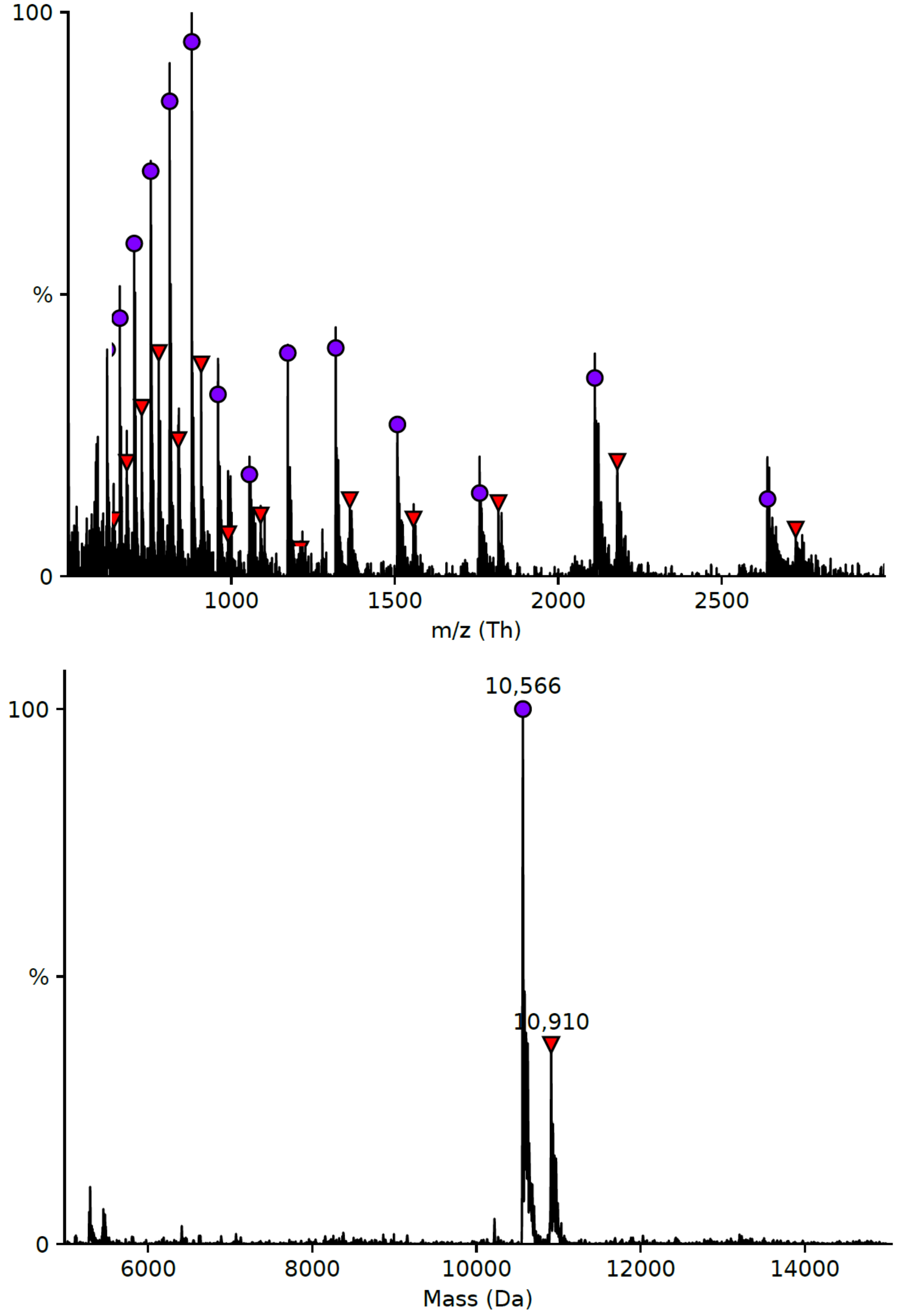

Figure S30. Mass spectra of **FAM_31_RNA**. a) raw spectrum; b) deconvoluted spectrum; calculated mass: 10566 Da, found mass: 10566 Da (**FAM_31_RNA**), found mass: 10910 Da (**FAM_31_RNA** + non-templated G).

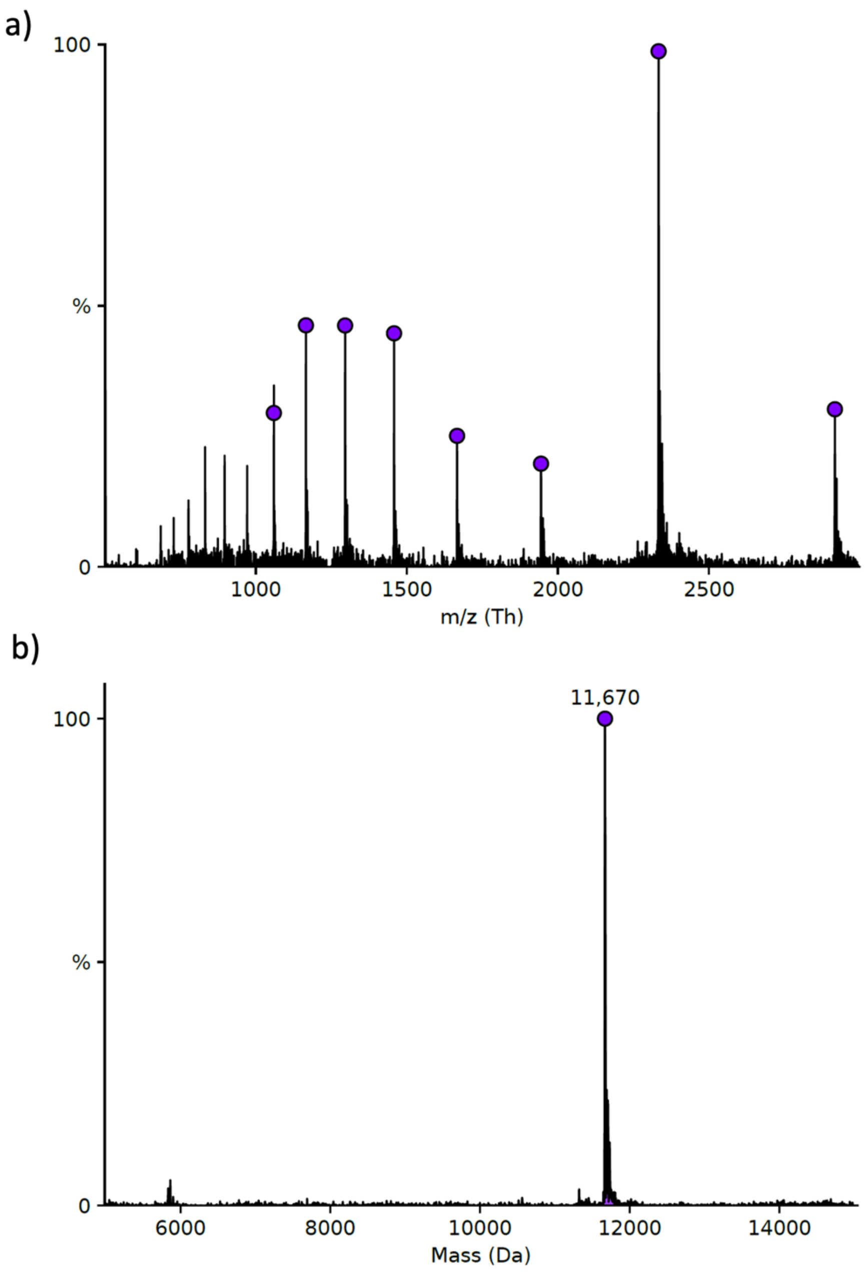

Figure S31. Mass spectra of **FAM_31_RNA_4C$^{\mathbf{ESQ}}$**. a) raw spectrum; b) deconvoluted spectrum; calculated mass: 11274 Da, found mass: 11670 Da (**FAM_31_RNA_4C$^{\mathbf{ESQ}}$** + non-templated G + $2Na^{+}$).

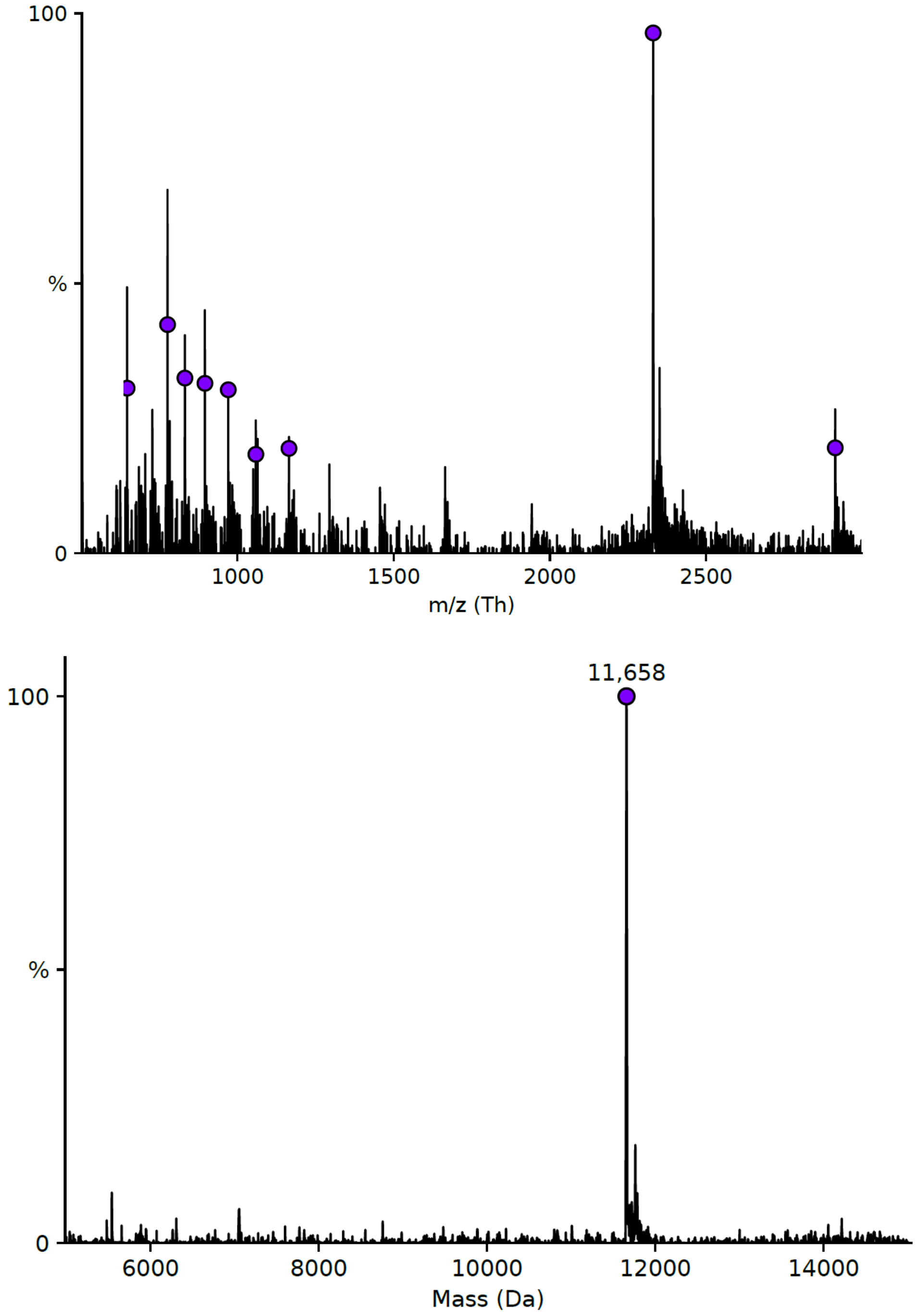

Figure S32. Mass spectra of **FAM_31_RNA_4A^{ESQ}**. a) raw spectrum; b) deconvoluted spectrum; calculated mass: 11274 Da, found mass: 11658 Da (**FAM_31_RNA_4A^{ESQ}** + non-templated G + K^+).

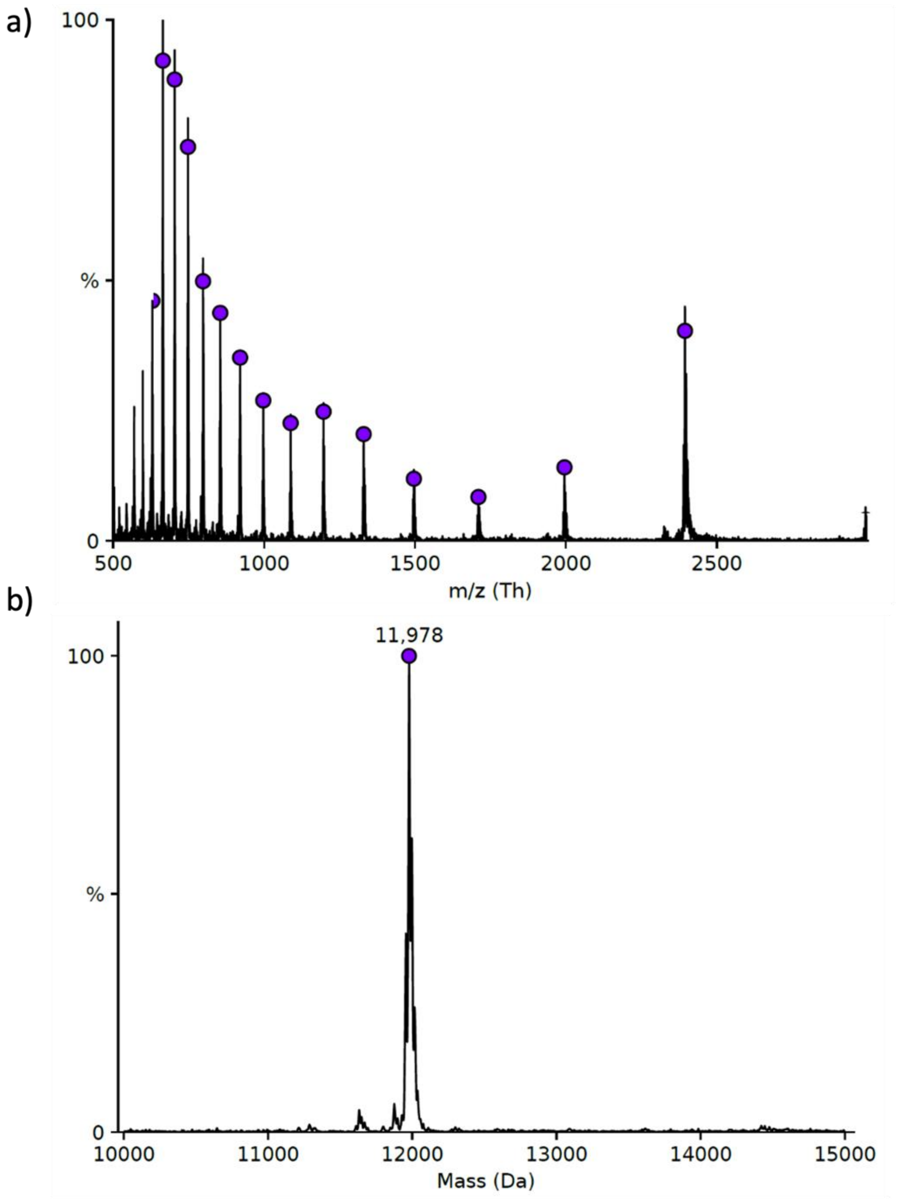

Figure S33. Mass spectra of **35_RNA_1C$^{\mathbf{ESQ}}$**. a) raw spectrum; b) deconvoluted spectrum; calculated mass: 11954 Da, found mass: 11978 Da (**35_RNA_1C$^{\mathbf{ESQ}}$** + Na^{+}).

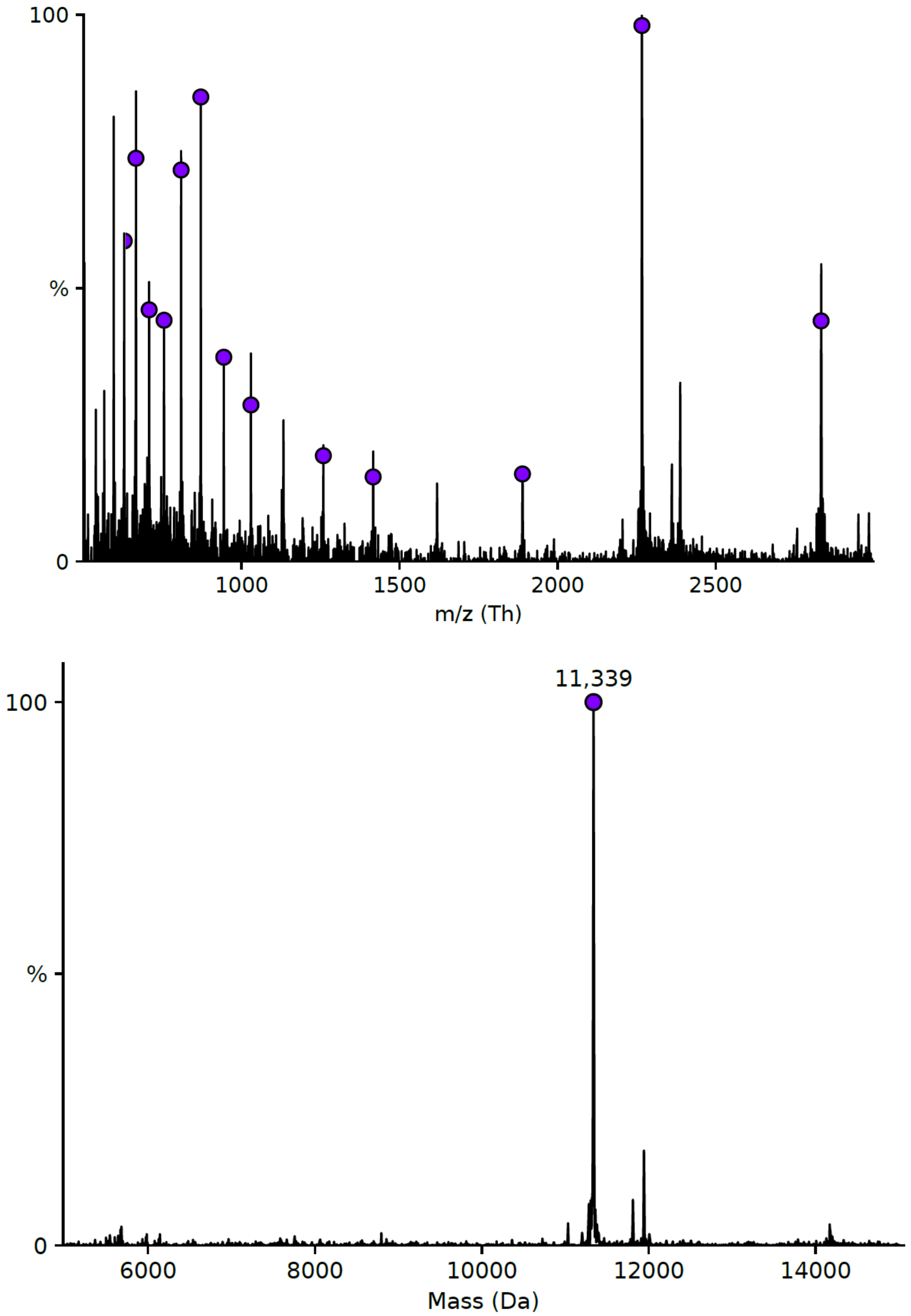

Figure S34. Mass spectra of **35_RNA_1A^{ESQ}**. a) raw spectrum; b) deconvoluted spectrum; calculated mass: 11313 Da, found mass: 11339 Da (**35_RNA_1A^{ESQ}** + Na^{+}).

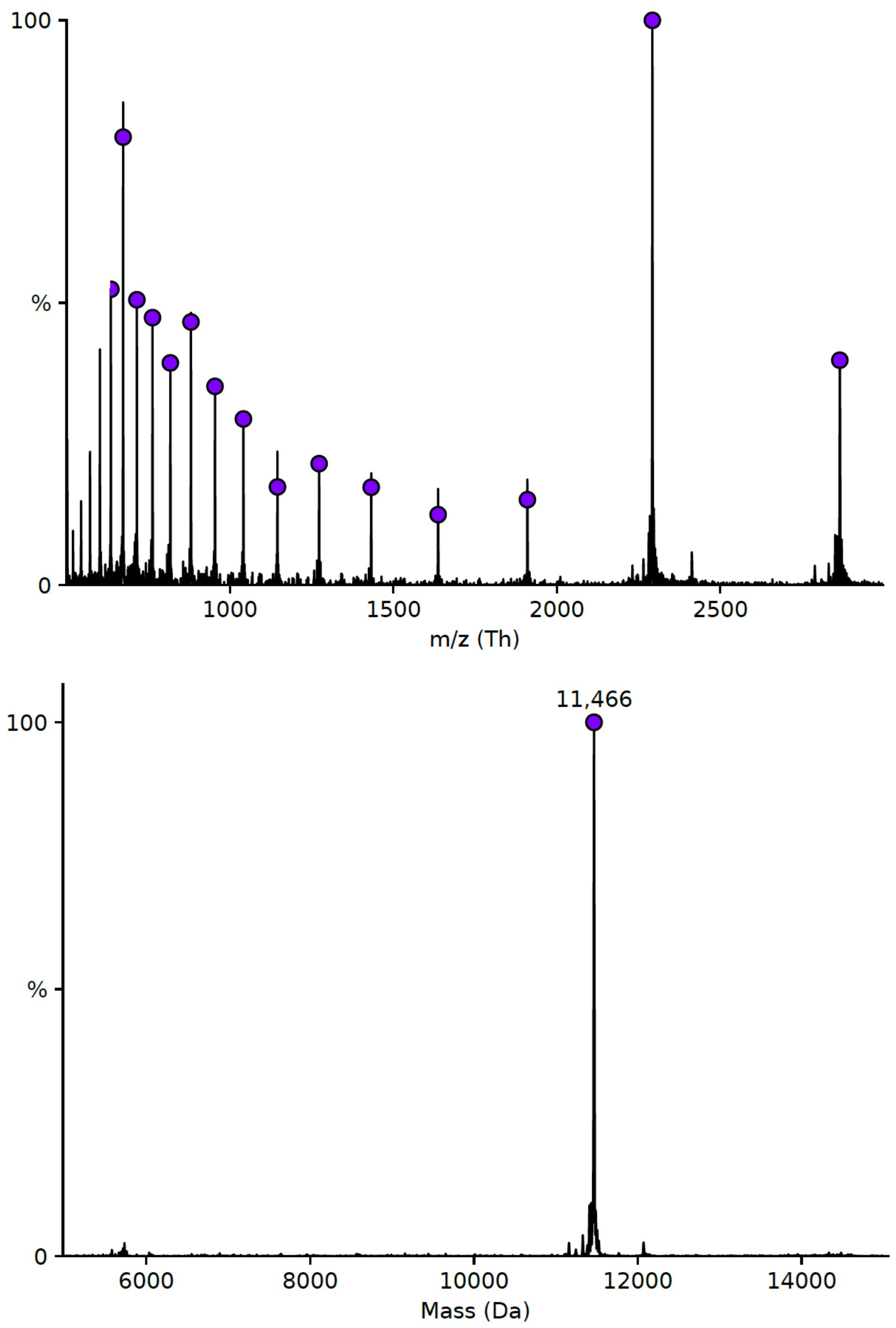

Figure S35. Mass spectra of **35_RNA_1A**$^{\mathbf{ESQ\_spermidine}}$. a) raw spectrum; b) deconvoluted spectrum; calculated mass: 11461 Da, found mass: 11466 Da (**35_RNA_1A**$^{\mathbf{ESQ\_spermidine}}$).

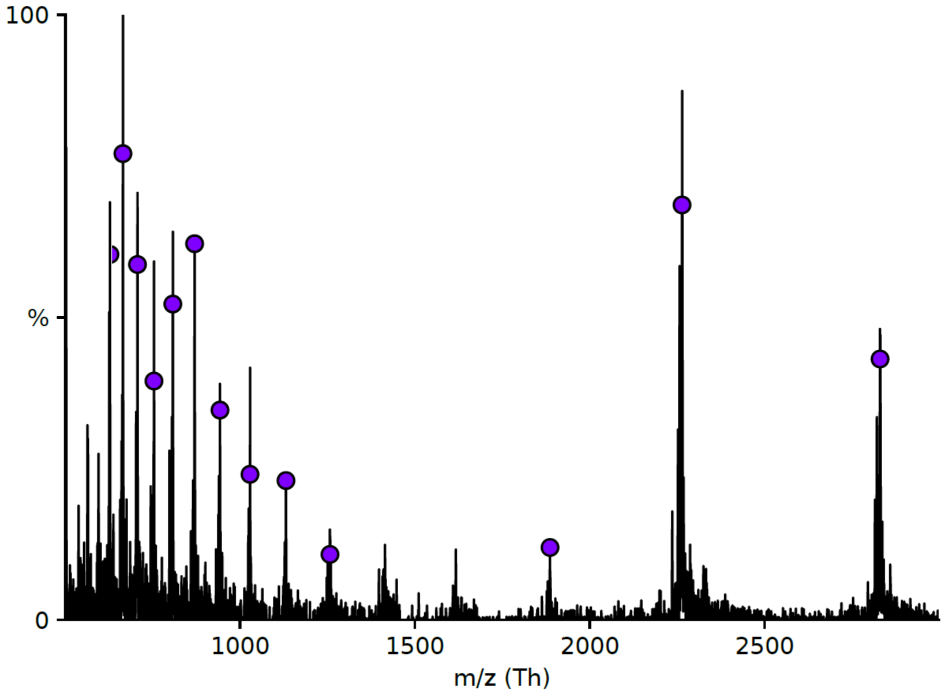

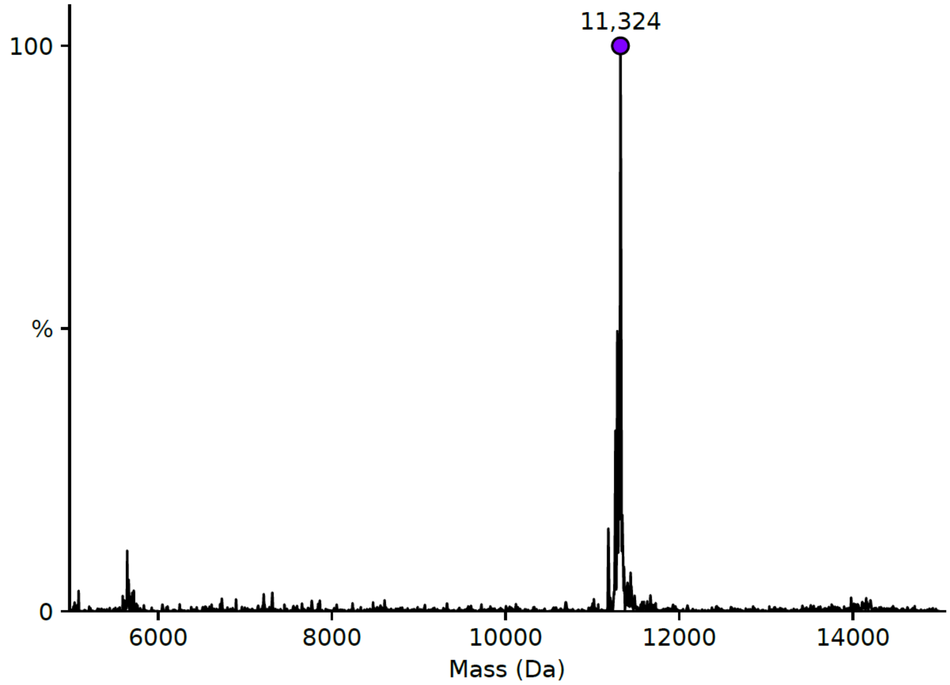

Figure S36. Mass spectra of **35_RNA_1A^{CA}**. a) raw spectrum; b) deconvoluted spectrum; calculated mass: 11268 Da, found mass: 11324 Da (**35_RNA_1A^{CA}** + K^+ + H_2O).

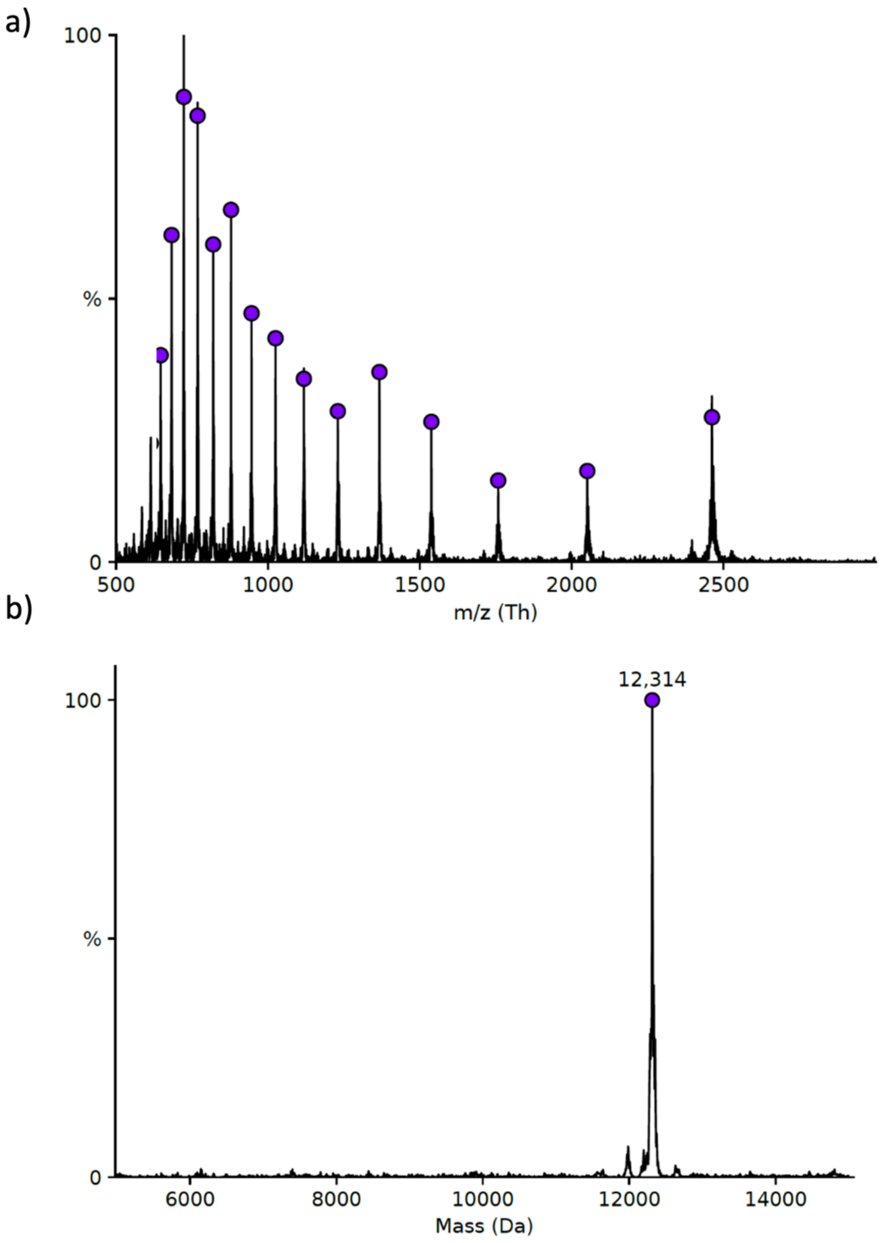

Figure S37. Mass spectra of **35_RNA_3C^{ESQ}**. a) raw spectrum; b) deconvoluted spectrum; calculated mass: 12275 Da, found mass: 12314 Da (**35_RNA_3C^{ESQ}** + K^{+}).

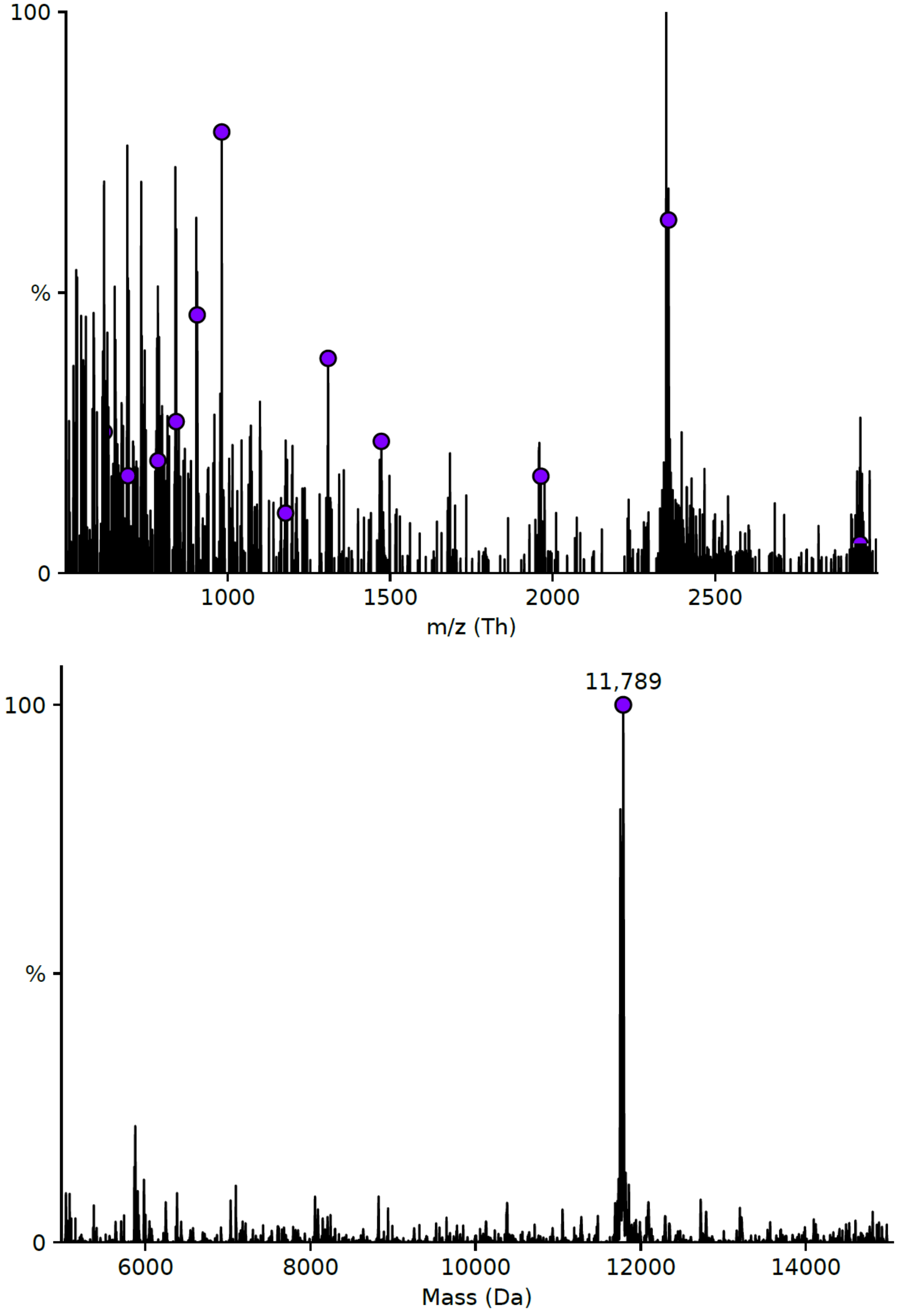

Figure S38. Mass spectra of **35_RNA_3A$^{\mathbf{ESQ}}$**. a) raw spectrum; b) deconvoluted spectrum; calculated mass: 11793 Da, found mass: 11789 Da (**35_RNA_3A$^{\mathbf{ESQ}}$**).

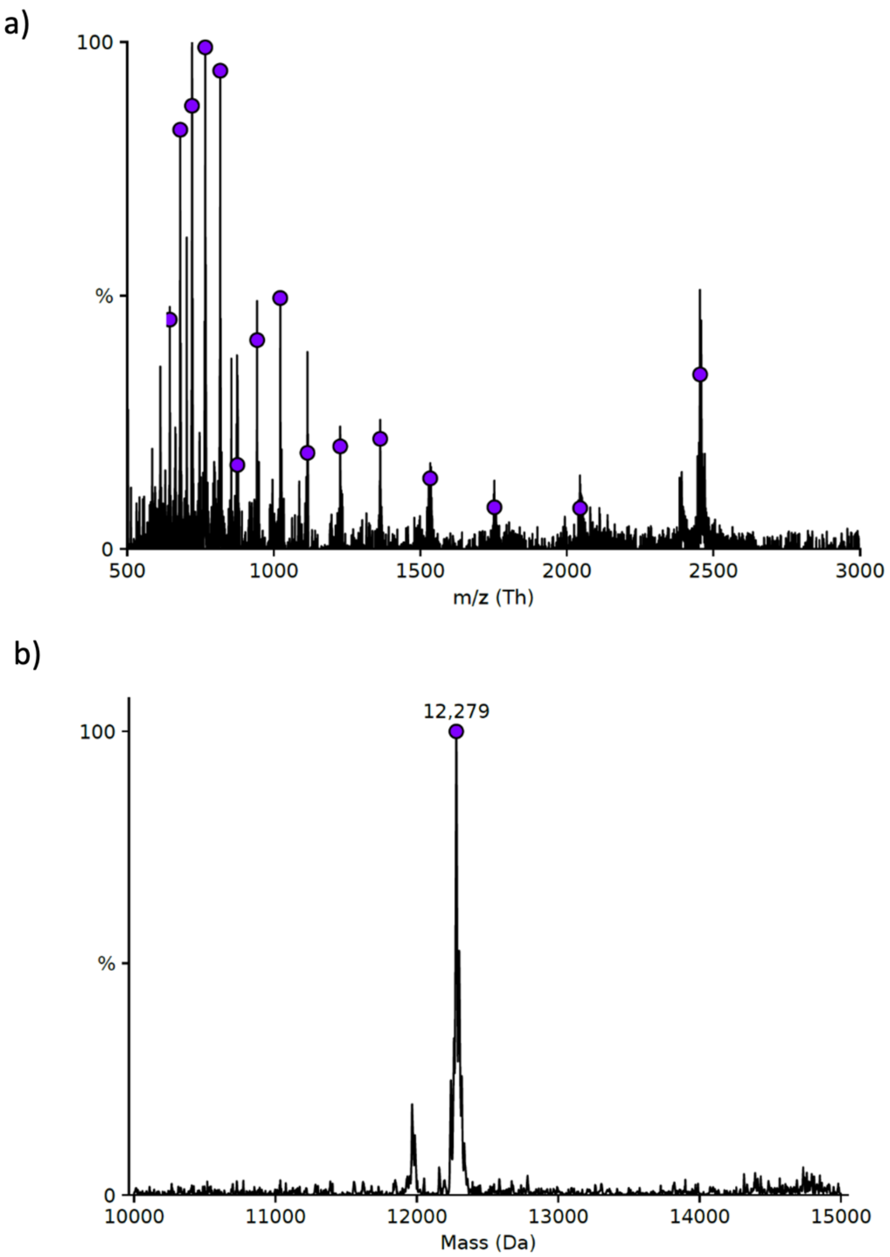

Figure S39. Mass spectra of **35_RNA_1C$^{\mathbf{ESQ\text{-}P}}$**. a) raw spectrum; b) deconvoluted spectrum; calculated mass: 12238 Da, found mass: 12279 Da (**35_RNA_1C$^{\mathbf{ESQ\text{-}P}}$** + K^+).

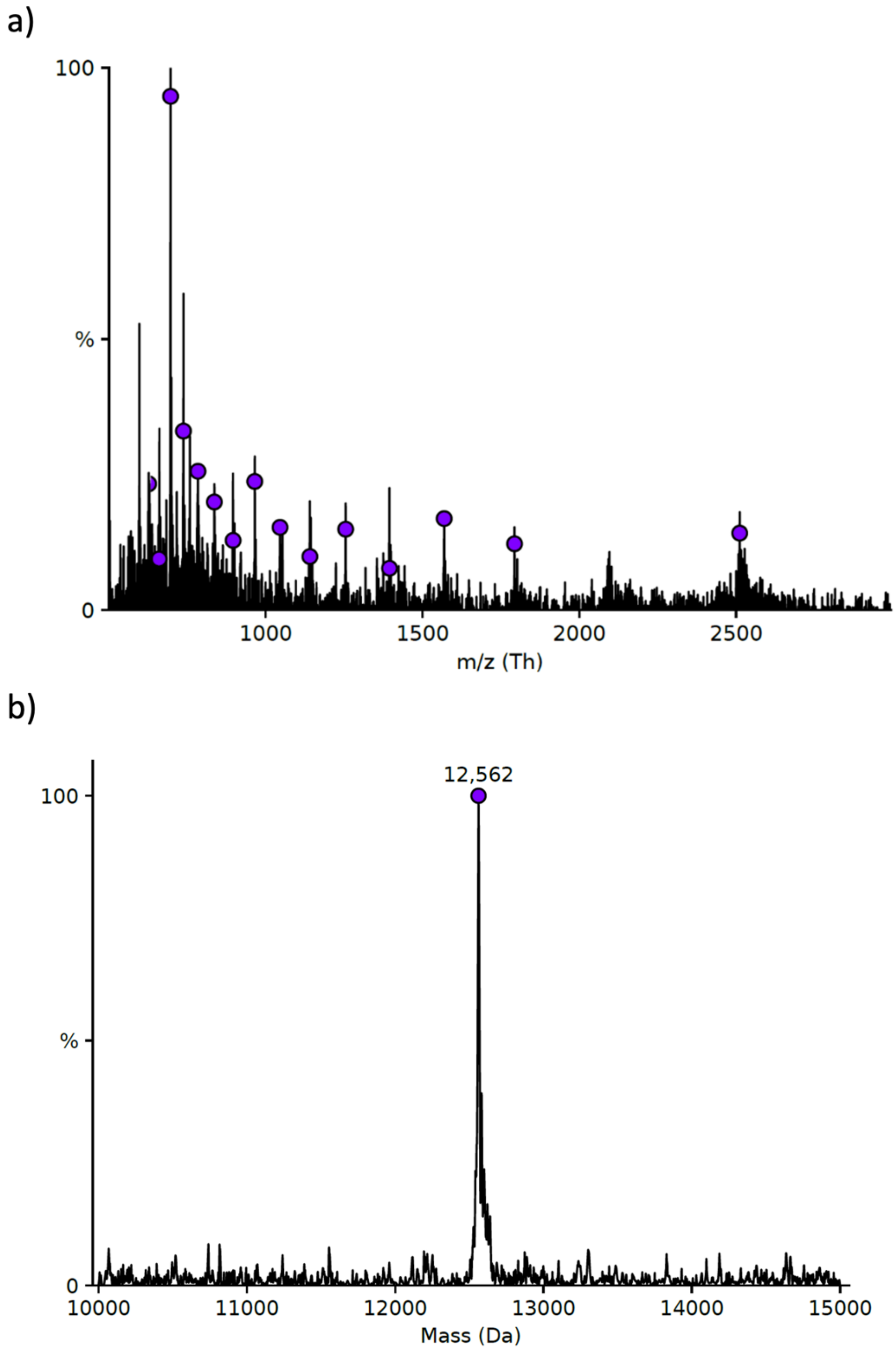

Figure S40. Mass spectra of **35_RNA_3C$^{\mathbf{ESQ\text{-}P}}$**. a) raw spectrum; b) deconvoluted spectrum; calculated mass: 13127 Da, found mass: 12562 Da (**35_RNA_3C$^{\mathbf{ESQ}}$** conjugated with one peptide molecule).

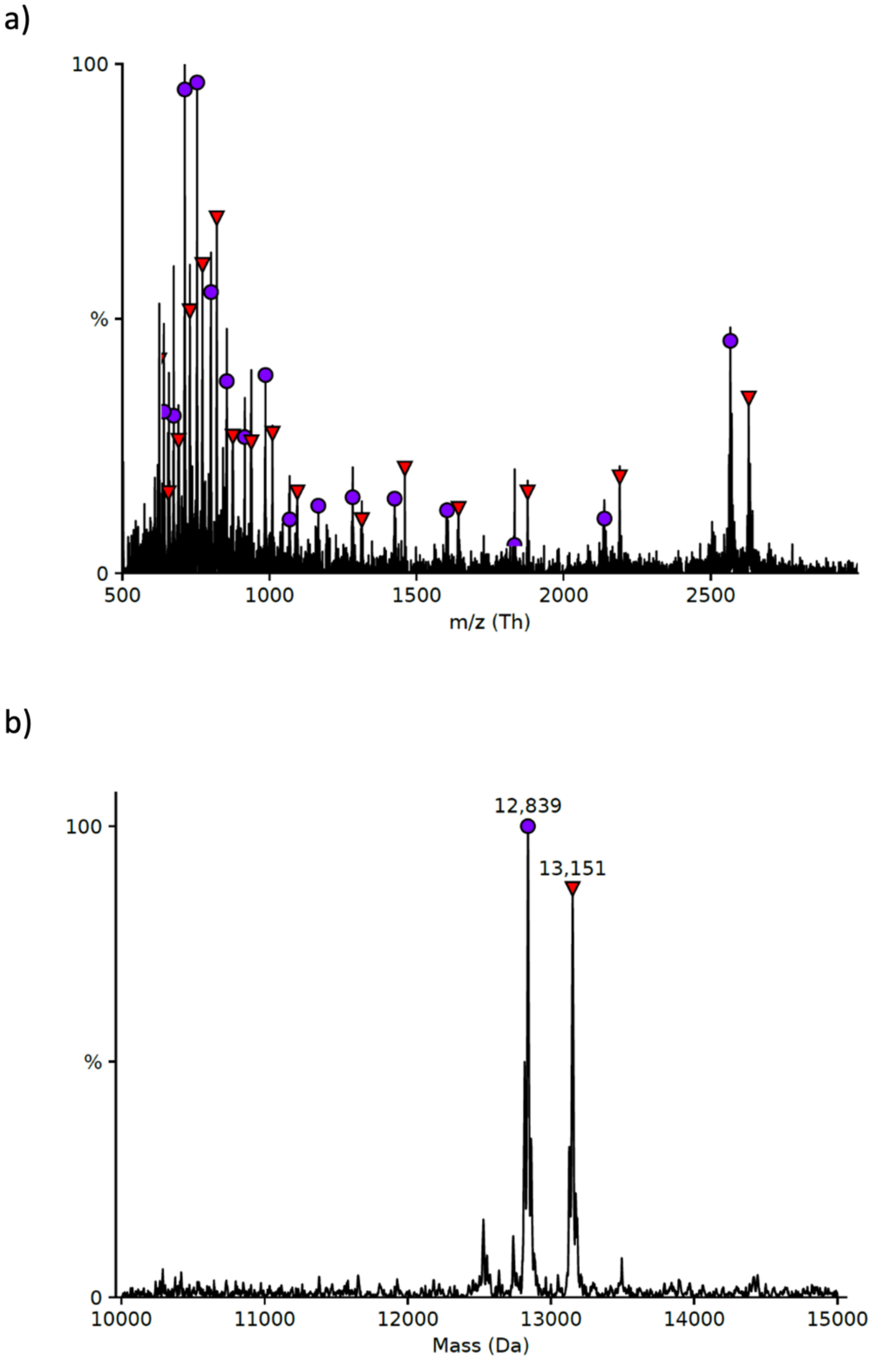

Figure S41. Mass spectra of **35_RNA_3C$^{\mathbf{ESQ\text{-}P}}$**. a) raw spectrum; b) deconvoluted spectrum; calculated mass: 13127 Da, found mass: 12839 Da (**35_RNA_3C$^{\mathbf{ESQ}}$** conjugated with two peptide molecules); found mass: 13151 Da (**35_RNA_3C$^{\mathbf{ESQ}}$** conjugated with three peptide molecules + Na^+).

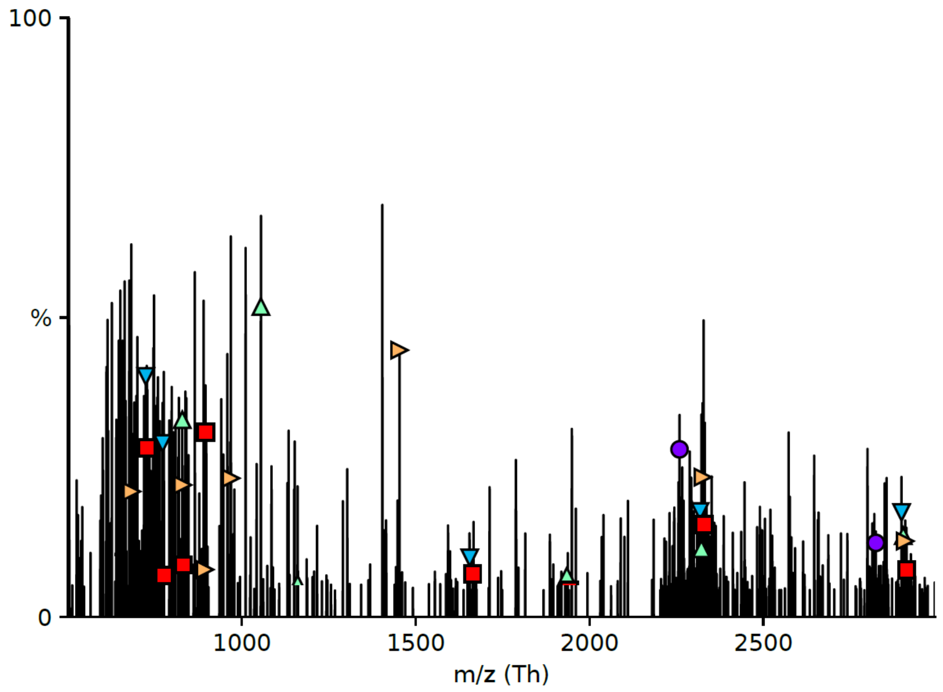

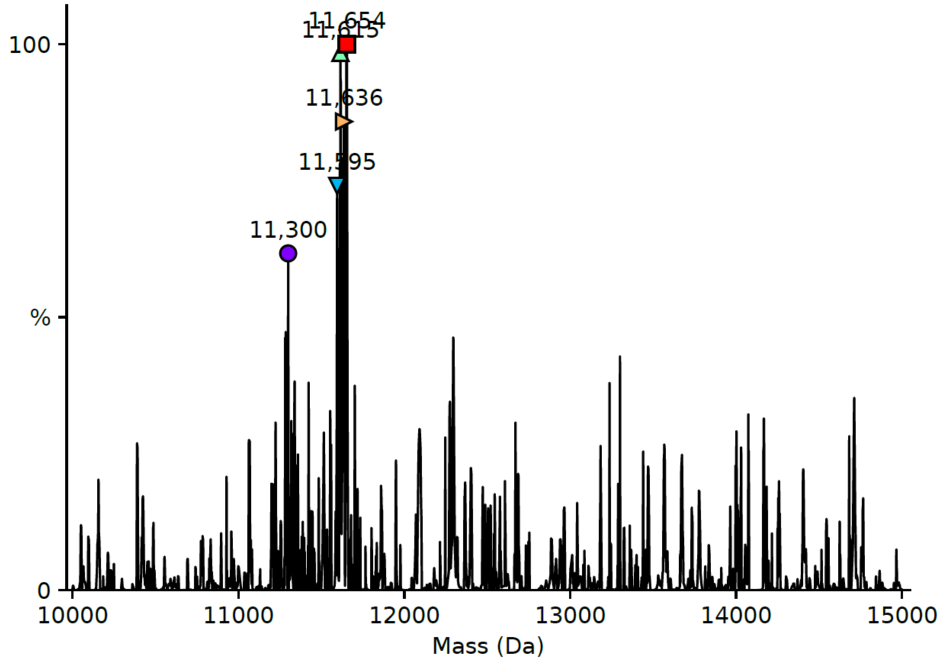

Figure S42. Mass spectra of **35_RNA_1A$^{\text{ESQ-P}}$**. a) raw spectrum; b) deconvoluted spectrum; calculated mass: 11597 Da, found mass: 11595 Da (**35_RNA_1A$^{\text{ESQ-P}}$**).

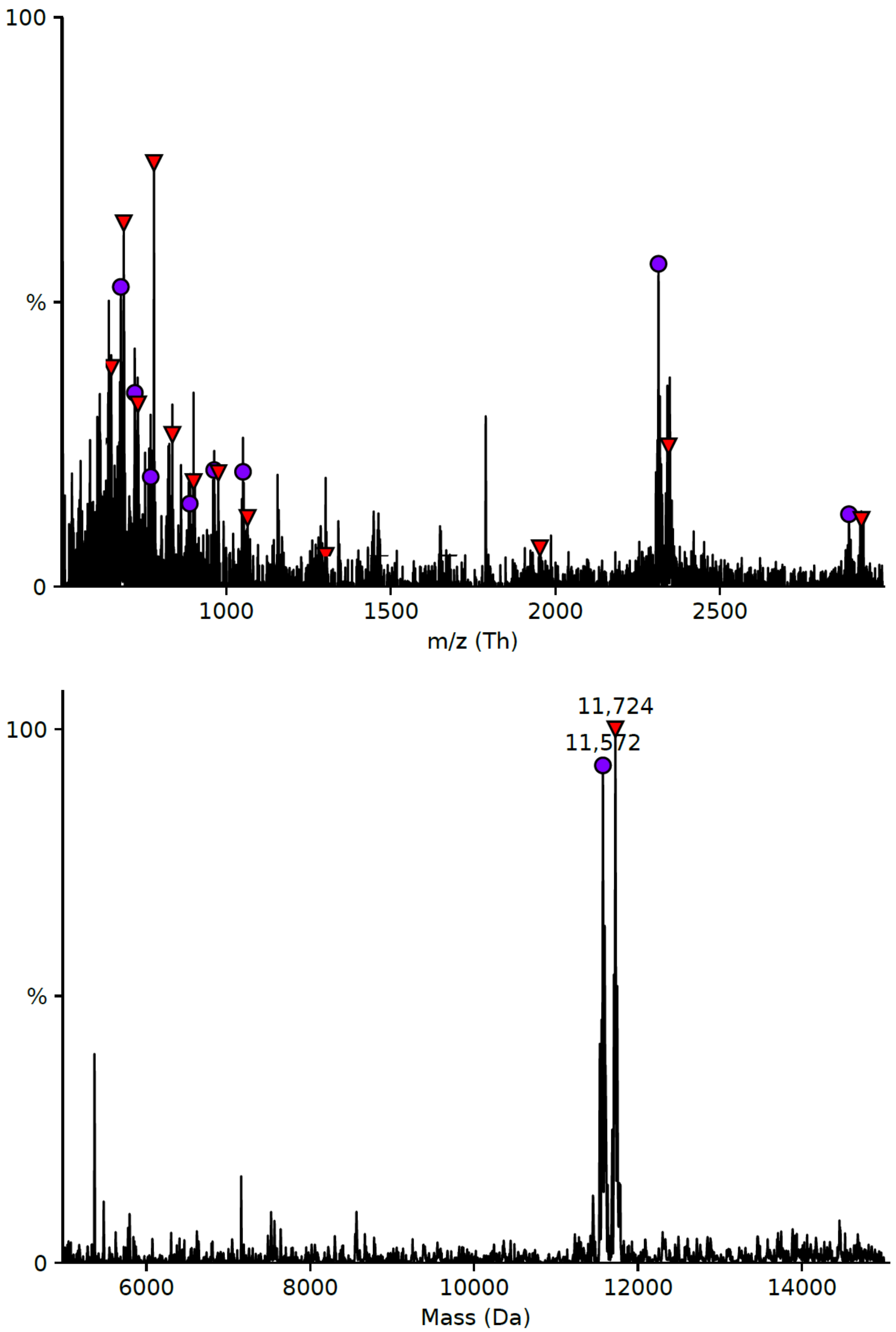

Figure S43. Mass spectra of **35_RNA_1A$^{CA\text{-}GSH}$**. a) raw spectrum; b) deconvoluted spectrum; calculated mass: 11539 Da, found mass: 11572 Da (**35_RNA_1A$^{CA\text{-}GSH}$** + K^+).

Intact mass measurement of 36_vRNA_1gC^{ESQ_SSB}

36_vRNA_1gC^{ESQ} containing one modification (**Table S1**) was cross-linked to SSB protein as described above.

The volume of 18 µl of the sample was injected onto a MassPREP Micro Desalting column (20 µm, 5-mm by 2.1-mm ID, Waters) and desalted and eluted by fast gradient (4 min). Mobile phase A (10 mM ammonium acetate in H_2O) and B (acetonitrile) were used for elution. The separation was carried out by AQUITY UPLC I-Class system (Waters) and was on-line coupled to Mass Spectrometer Synapt G2 (Waters) to acquire mass spectra using electrospray ionization in positive mode. The TOF mass range was set from *m/z* 500 to 4000. The raw spectrum was subtracted and deconvoluted (MaxEnt 1, Waters) to produce the final spectrum (**Figure S44**).

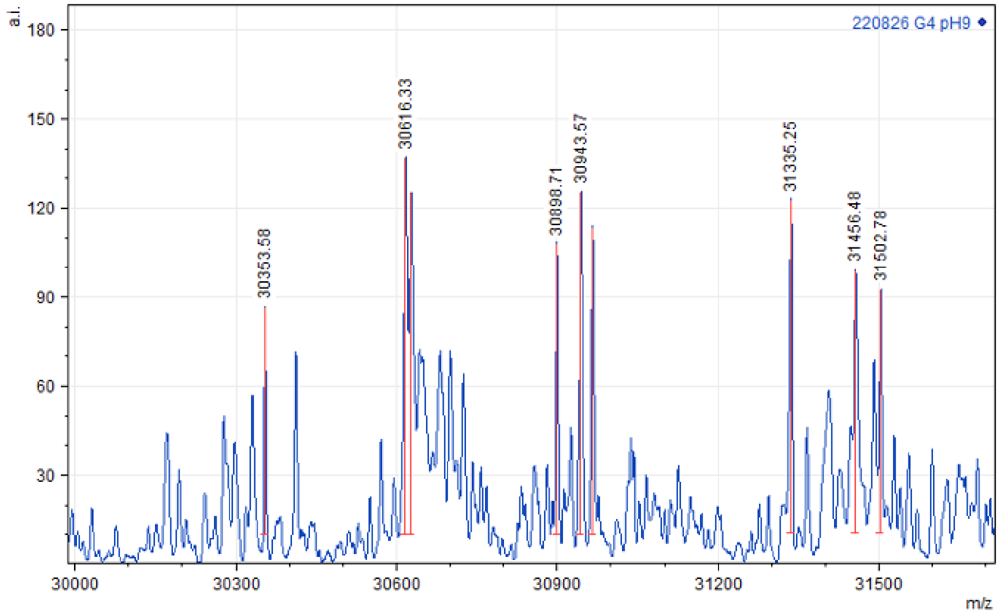

Figure S44. Spectrum of intact mass measurement of **36_vRNA_1gC^{ESQ_SSB}**. M (calc.) = 30920.52 Da, M (found) = 30943.57 [M + Na]$^+$. Peak at 30616.33 Da is [M - UMP]$^+$.

LC-MS analysis of digested 46_RNA_HP^{C4_JEVNS5} samples after polymerase assay

The JEV NS5 mediated RNA extension assay was performed as described above (20 × 20 µl reaction mixture), except the reaction was incubated overnight. The reaction was followed by trypsine digestion of the protein to peptides and RNA digestion by RNase A/T1 mix.

Samples were dissolved in 15 µl of 0.1 TFA in H_2O and 3 µl of the sample was injected on an UltiMate 3000 RSLCnano system (Thermo Fisher Scientific) coupled to a Mass Spectrometer Orbitrap Fusion Lumos Tribrid (Thermo Fisher Scientific). The peptides were trapped on a PepMap100 column (5 µm, 5 mm by 300-µm internal diameter (ID); Thermo Fisher Scientific) and desalted with 2% acetonitrile in 0.100% formic acid at a flow rate of 5 µL min^{-1}. Eluted peptides were separated using an EASY-Spray PepMap100 C18 analytical column (2 µm, 50-cm by 75-µm ID; Thermo Fisher Scientific). The 30-min elution gradient at a constant flow rate of 300 nL/min was set to start at 5% phase B (0.100% formic acid in 99.9% acetonitrile) and 95% phase A (0.100% formic acid). Then, the content of acetonitrile was increased gradually up to 50% of phase B. The orbitrap mass range was set from *m/z* 350 to 2000 in the MS mode, and for ions with a charge state 2-6 the fragmentation spectra were acquired. A Proteome Discoverer 2.5 (Thermo Fisher Scientific) was used for identification of peptide and protein using Sequest HS and MS Amanda as search engines and databases of protein sequence and common contaminants (**Table S6; Figure S45-49**).

Table S6. NanoLC-ESI$^+$-MS/MS data of tryptic peptides sequences bearing the lysine residue targeted by modification.

Peptides sequence	$[M + H]^{3+}$ (found) [Da]	$[M + H]^+$ (found) [Da]	$[M + H]^+$ (calc.) [Da]
NH2-AVG**K***GEVHSNQEK-COOH	586.26	1756.79	1756.70
NH2-E**K***KPGEFGK-COOH	465.22	1393.64	1393.84
NH2-EK**K***PGEFGK-COOH	465.22	1393.64	1393.84

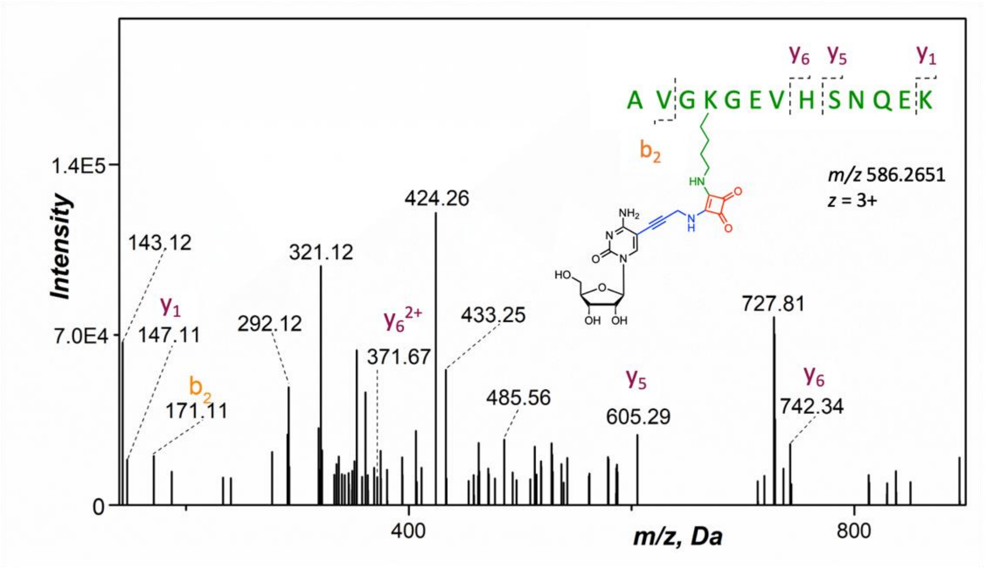

Figure S45. NanoLC-ESI$^+$-MS/MS spectrum of **46_RNA_HP^{C4_JEVNS5}** identification of K269 (m/z acquired: 586.26 Da).

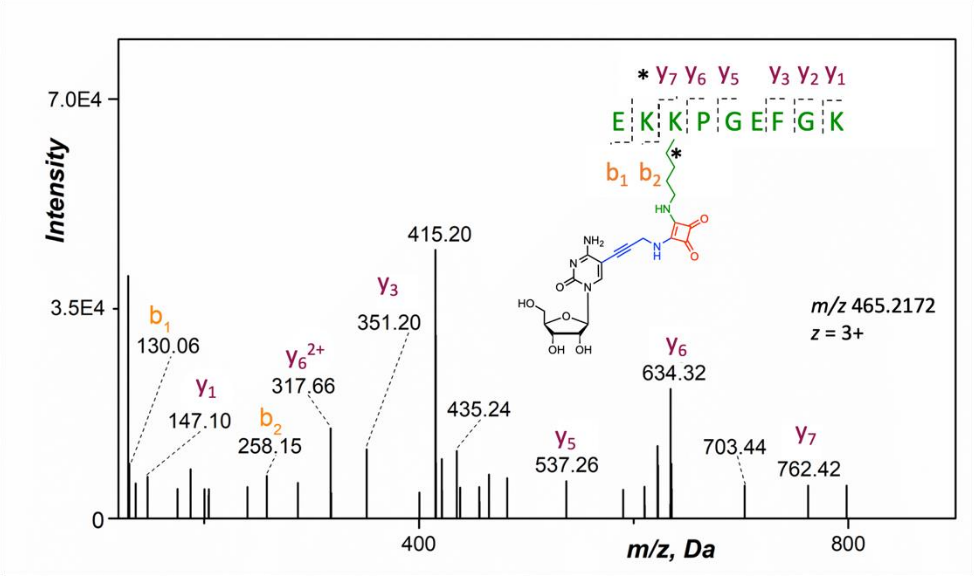

Figure S46. NanoLC-ESI$^+$-MS/MS spectrum of **46_RNA_HP^{C4_JEVNS5}** identification of K462/463 (m/z acquired: 465.22 Da).

JEV NS5 protein sequence

```
        10         20         30         40         50         60
GRPGGRTLGE QWKEKLNAMS REEFFKYRRE AIIEVDRTEA RRARRENNIV GGHPVSRGSA

        70         80         90        100        110        120
KLRWLVEKGF VSPIGKVIDL GCGRGGWSYY AATLKKVQEV RGYTKGGAGH EEPMLMQSYG

       130        140        150        160        170        180
RNLVSLKSGV DVFYKPSEPS DTLFCDIGES SPSPEVEEQR TLRVLEMTSD WLHRGPREFC

       190        200        210        220        230        240
IKVLCPYMPK VIEKMEVLQR RFGGGLVRLP LSRNSNHEMY WVSGAAGNVV HAVNMTSQVL

       250        260        269          280         290         300
LGRMDRTVWR GPKYEEDVNL GSGTRAVGK*G EVHSNQEKIK KRIQKLKEEF ATTWHKDPEH

       310        320        330        340        350        360
PYRTWTYHGS YEVKATGSAS SLVNGVVKLM SKPWDAIANV TTMAMTDTTP FGQQRVFKEK

       370        380        390        400        410        420
VDTKAPEPPA GAKEVLNETT NWLWAHLSRE KRPRLCTKEE FIKKVNSNAA LGAVFAEQNQ

       430        440        450        460        470        480
WSTAREAVDD PRFWEMVDEE RENHLRGECH TCIYNMMGKR EKKPGEFGKA KGSRAIWFMW

       490        500        510        520        530        540
LGARYLEFEA LGFLNEDHWL SRENSGGGVE GSGVQKLGYI LRDIAGKQGG KMYADDTAGW

       550        560        570        580        590        600
DTRITRTDLE NEAKVLELLD GEHRMLARAI IELTYRHKVV KVMRPAAEGK TVMDVISRED

       610        620        630        640        650        660
QRGSGQVVTY ALNTFTNIAV QLVRLMEAEG VIGPQHLEQL PRKTKIAVRT WLFENGEERV

       670        680        690        700        710        720
TRMAISGDDC VVKPLDDRFA TALHFLNAMS KVRKDIQEWK PSHGWHDWQQ VPFCSNHFQE

       730        740        750        760        770        780
IVMKDGRSIV VPCRGQDELI GRARISPGAG WNVKDTACLA KAYAQMWLLL YFHRRDLRLM

       790        800        810        820        830        840
ANAICSAVPV DWVPTGRTSW SIHSKGEWMT TEDMLQVWNR VWIEENEWMM DKTPITSWTD

       850        860        870        880        890        900
VPYVGKREDI WCGSLIGTRS RATWAENIYA AINQVRAVIG KENYVDYMTS LRRYEDVLIQ

EDRVI
```

Figure S47. Sequence coverage (91%) of the JEV NS5 proteomic analysis from sample 1. The identified parts of protein are highlighted in green, peptide with RNA modification is underlined and in red. K* = $\mathbf{C}^{\mathbf{ESQ}}$ modified lysine K269.

JEV NS5 protein sequence

```
        10         20         30         40         50         60
GRPGGRTLGE QWKEKLNAMS REEFFKYRRE AIIEVDRTEA RRARRENNIV GGHPVSRGSA

        70         80         90        100        110        120
KLRWLVEKGF VSPIGKVIDL GCGRGGWSYY AATLKKVQEV RGYTKGGAGH EEPMLMQSYG

       130        140        150        160        170        180
RNLVSLKSGV DVFYKPSEPS DTLFCDIGES SPSPEVEEQR TLRVLEMTSD WLHRGPREFC

       190        200        210        220        230        240
IKVLCPYMPK VIEKMEVLQR RFGGGLVRLP LSRNSNHEMY WVSGAAGNVV HAVNMTSQVL

       250        260        270        280        290        300
LGRMDRTVWR GPKYEEDVNL GSGTRAVGKG EVHSNQEKIK KRIQKLKEEF ATTWHKDPEH

       310        320        330        340        350        360
PYRTWTYHGS YEVKATGSAS SLVNGVVKLM SKPWDAIANV TTMAMTDTTP FGQQRVFKEK

       370        380        390        400        410        420
VDTKAPEPPA GAKEVLNETT NWLWAHLSRE KRPRLCTKEE FIKKVNSNAA LGAVFAEQNQ

       430        440        450        459 462/3                480
WSTAREAVDD PRFWEMVDEE RENHLRGECH TCIYNMMGKR EK*K*PGEFGKA KGSRAIWFMW

       490        500        510        520        530        540
LGARYLEFEA LGFLNEDHWL SRENSGGGVE GSGVQKLGYI LRDIAGKQGG KMYADDTAGW

       550        560        570        580        590        600
DTRITRTDLE NEAKVLELLD GEHRMLARAI IELTYRHKVV KVMRPAAEGK TVMDVISRED

       610        620        630        640        650        660
QRGSGQVVTY ALNTFTNIAV QLVRLMEAEG VIGPQHLEQL PRKTKIAVRT WLFENGEERV

       670        680        690        700        710        720
TRMAISGDDC VVKPLDDRFA TALHFLNAMS KVRKDIQEWK PSHGWHDWQQ VPFCSNHFQE

       730        740        750        760        770        780
IVMKDGRSIV VPCRGQDELI GRARISPGAG WNVKDTACLA KAYAQMWLLL YFHRRDLRLM

       790        800        810        820        830        840
ANAICSAVPV DWVPTGRTSW SIHSKGEWMT TEDMLQVWNR VWIEENEWMM DKTPITSWTD

       850        860        870        880        890        900
VPYVGKREDI WCGSLIGTRS RATWAENIYA AINQVRAVIG KENYVDYMTS LRRYEDVLIQ

EDRVI
```

Figure S48. Sequence coverage (88%) of the JEV NS5 proteomic analysis from sample 2. The identified parts of protein are highlighted in green, peptide with RNA modification is underlined and in red. K* = **C**[ESQ] modified lysine K462/463.

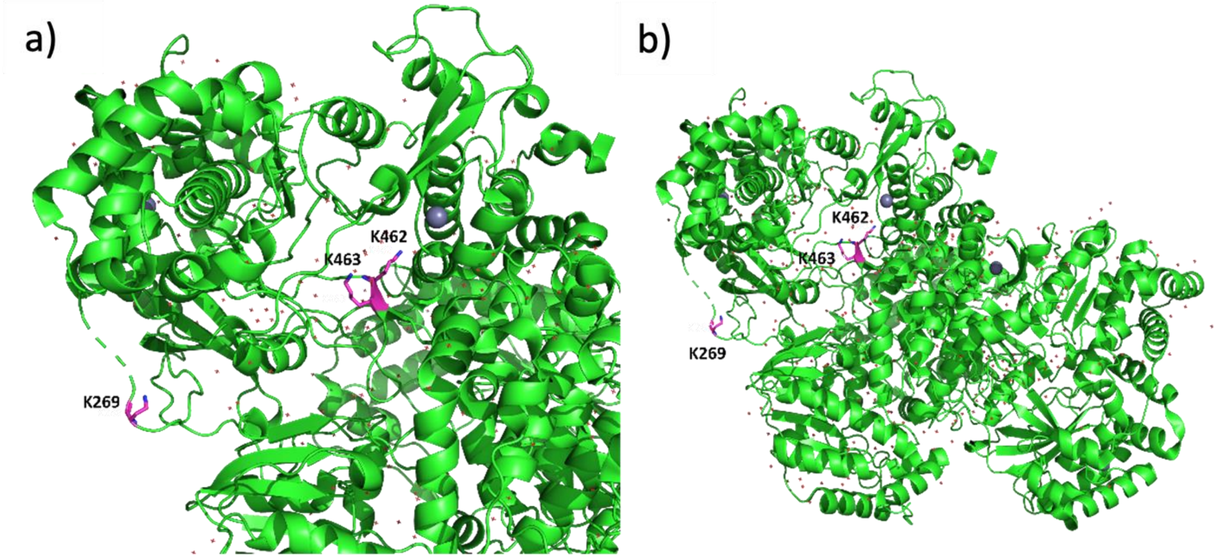

Figure S49. Crystal structure of JEV NS5 RdRp (PDB structure 4K6M) showing position of lysine residues participating in RNA-protein cross-link formation. a) Detail view b) crystal structure of full length JEV NS5. Identified cross-linked lysines are in magenta.

2. Supplementary References

[1] Hashmi, A. S. K. *et al.* Gold catalysis: Non-spirocyclic intermediates in the conversion of furanynes by the formal insertion of an alkyne into an aryl-alkyl C-C single bond. *Chem. Eur. J.* **18**, 10480–10486 (2012).

[2] Garg, N. K., Woodroofe, C. C., Lacenere, C. J., Quake, S. R. & Stoltz, B. M. A ligand-free solid-supported system for Sonogashira couplings: Applications in nucleoside chemistry. *Chem. Commun.* **36**, 4551–4553 (2005).

[3] Asare-Okai, P. N., Agustin, E., Fabris, D. & Royzen, M. Site-specific fluorescence labelling of RNA using bio-orthogonal reaction of trans-cyclooctene and tetrazine. *Chem. Commun.* **50**, 7844–7847 (2014).

[4] Konkolova, E. *et al.* Remdesivir triphosphate can efficiently inhibit the RNA-dependent RNA polymerase from various flaviviruses. *Antiviral Res.* **182**, 104899 (2020).

[5] Dejmek, M. *et al.* Non-nucleotide RNA-dependent RNA polymerase inhibitor that blocks SARS-CoV-2 replication. *Viruses* **13**, 1585 (2021).

[6] Andersen, T. E., Kirpekar, F. & Haselmann, K. F. RNA fragmentation in MALDI mass spectrometry studied by H/D-exchange: mechanisms of general applicability to nucleic acids. *J. Am. Soc. Mass. Spectrom.* **10** ,1353-1368 (2006).

[7] Marty, M. T. et al. Bayesian deconvolution of mass and ion mobility spectra: From binary interactions to polydisperse ensembles. *Anal. Chem.* **87**, 4370–4376 (2015).
