# Supplementary material for: Enzymatic synthesis of reactive RNA probes containing squaramate-linked cytidine or adenosine for bioconjugations and cross-linking with lysine-containing peptides and proteins
Source: Commun Chem. 2025 Jan 2;8:1. doi: 10.1038/s42004-024-01399-6 (PMC11696893; doi:10.1038/s42004-024-01399-6)
Supplement: Supplementary file 3 — Description of Additional Supplementary Files [file 42004_2024_1399_MOESM3_ESM.pdf]

Description of Additional Supplementary Files

File name: Supplementary Data 1

Description: NMR data

File name: Supplementary Data 2

Description: Uncropped Gels from Figs. 1 - 5
