# Supplementary material for: Enzymatic synthesis of reactive RNA probes containing squaramate-linked cytidine or adenosine for bioconjugations and cross-linking with lysine-containing peptides and proteins
Source: Commun Chem. 2025 Jan 2;8:1. doi: 10.1038/s42004-024-01399-6 (PMC11696893; doi:10.1038/s42004-024-01399-6)
Supplement: Supplementary file 4 — Supplementary Data 1 [file 42004_2024_1399_MOESM4_ESM.pdf]

^{1}H, ^{13}C, ^{19}F NMR spectra of C^{PACF3}

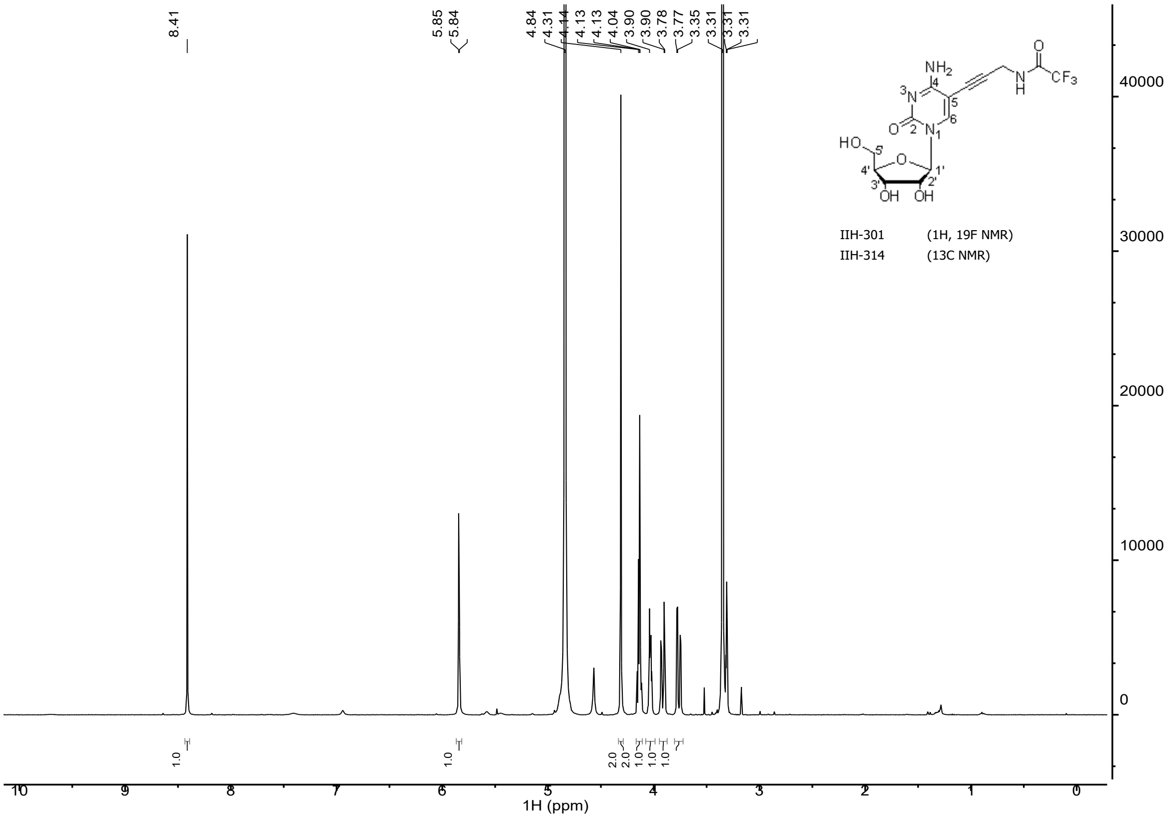

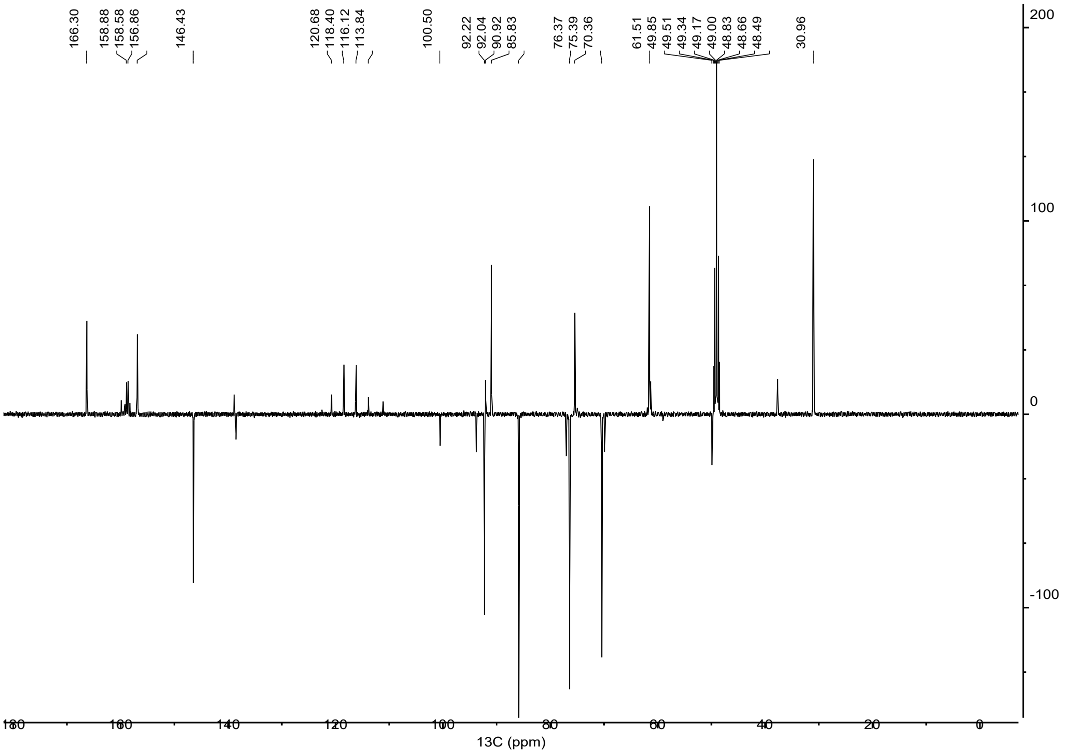

166.30
158.88
158.58
156.86
146.43
120.68
118.40
116.12
113.84
100.50
92.22
92.04
90.92
85.83
76.37
75.39
70.36
61.51
49.85
49.51
49.34
49.17
49.00
48.83
48.66
48.49
30.96
13C (ppm)

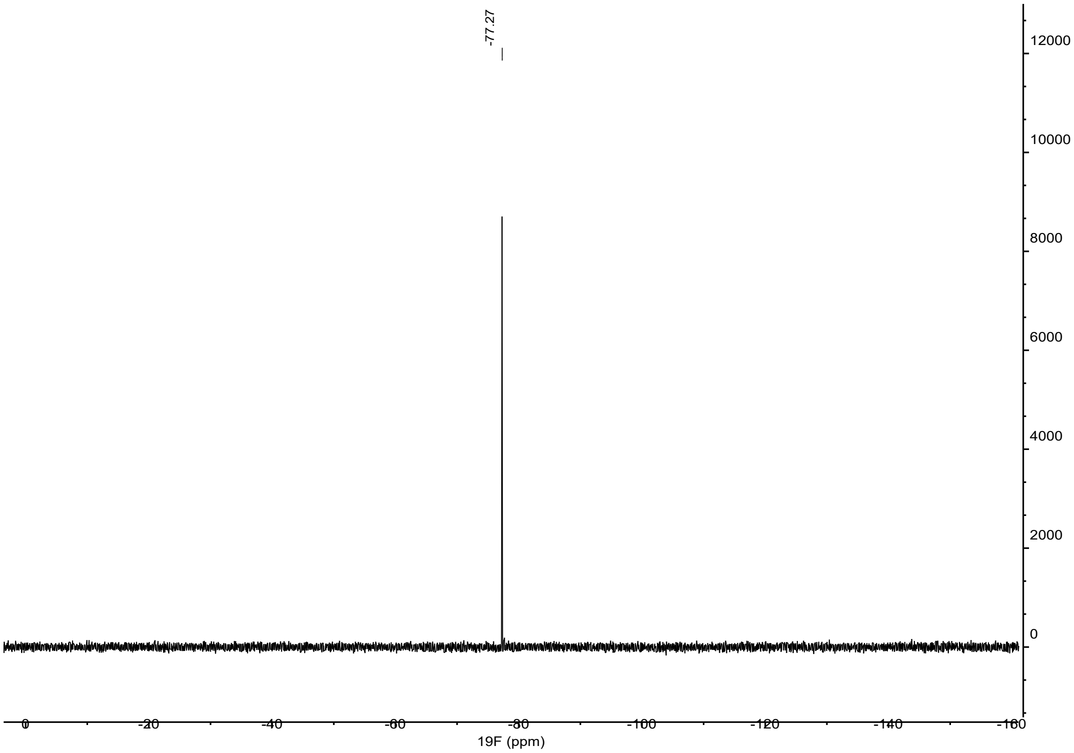

-77.27
19F (ppm)

^{1}H NMR spectra of C^{PA}

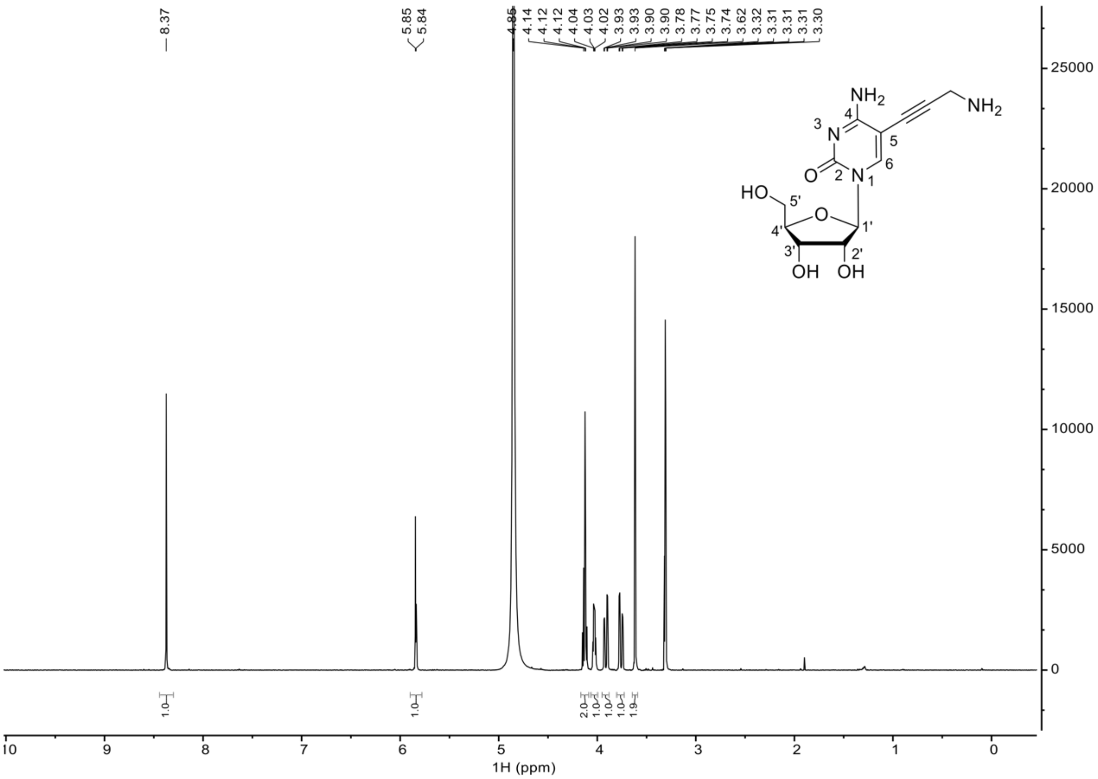

^{1}H, ^{13}C NMR spectra of C^{ESQ}

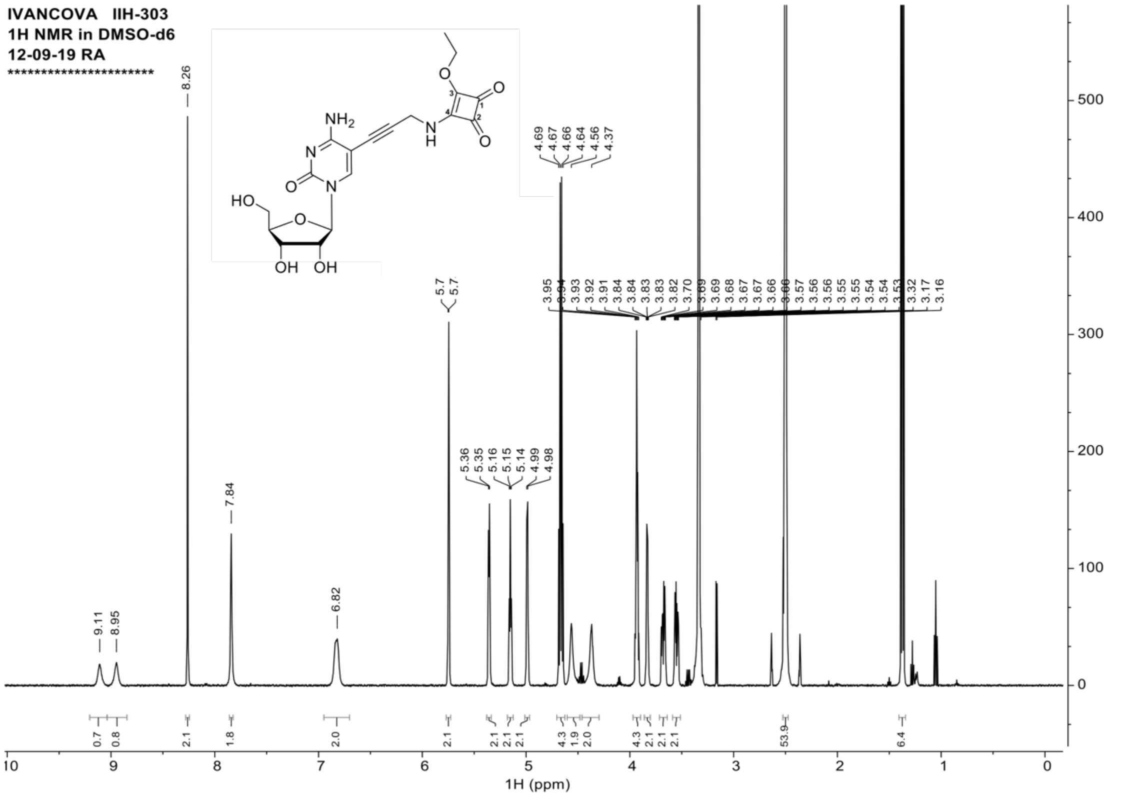

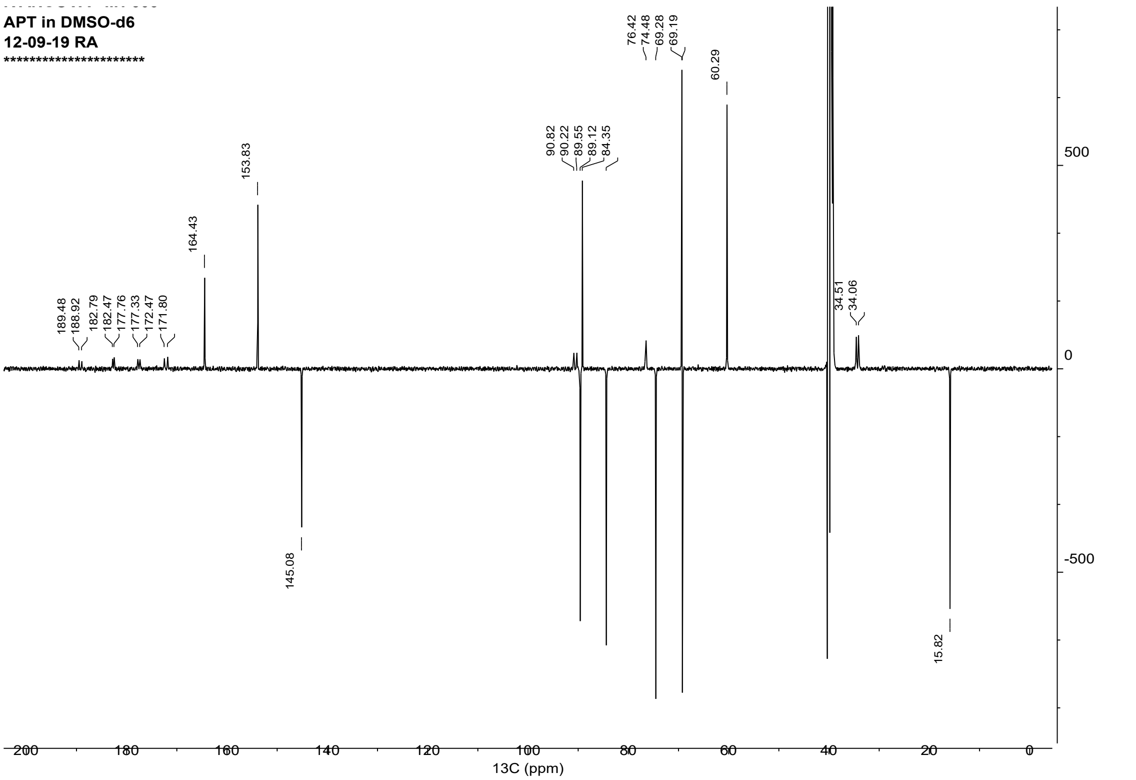

^{1}H, ^{13}C, ^{31}P NMR spectra of C^{ESQ}TP

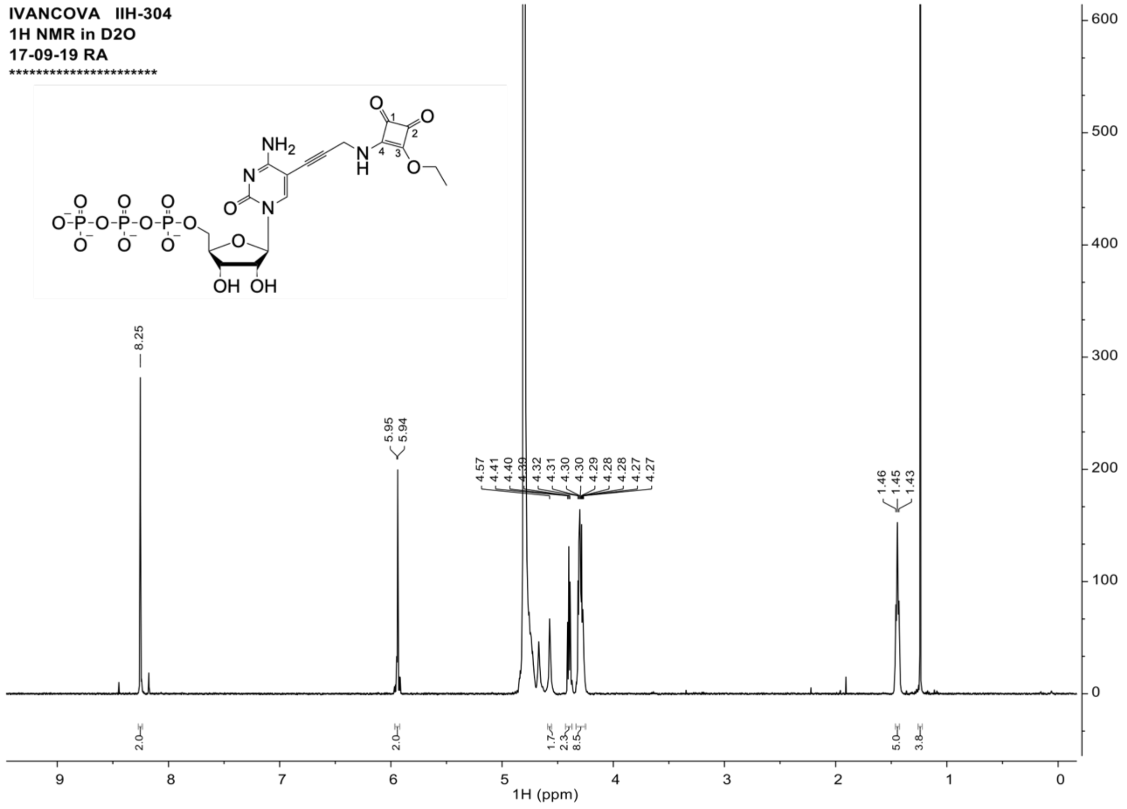

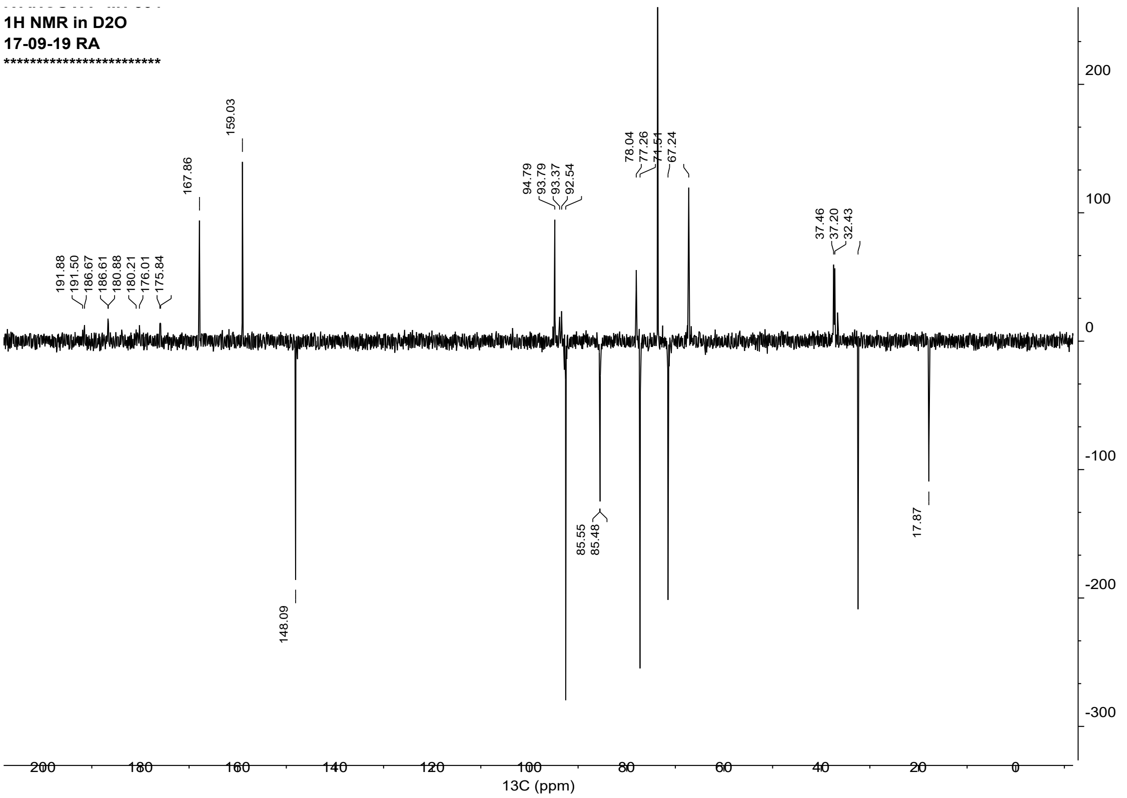

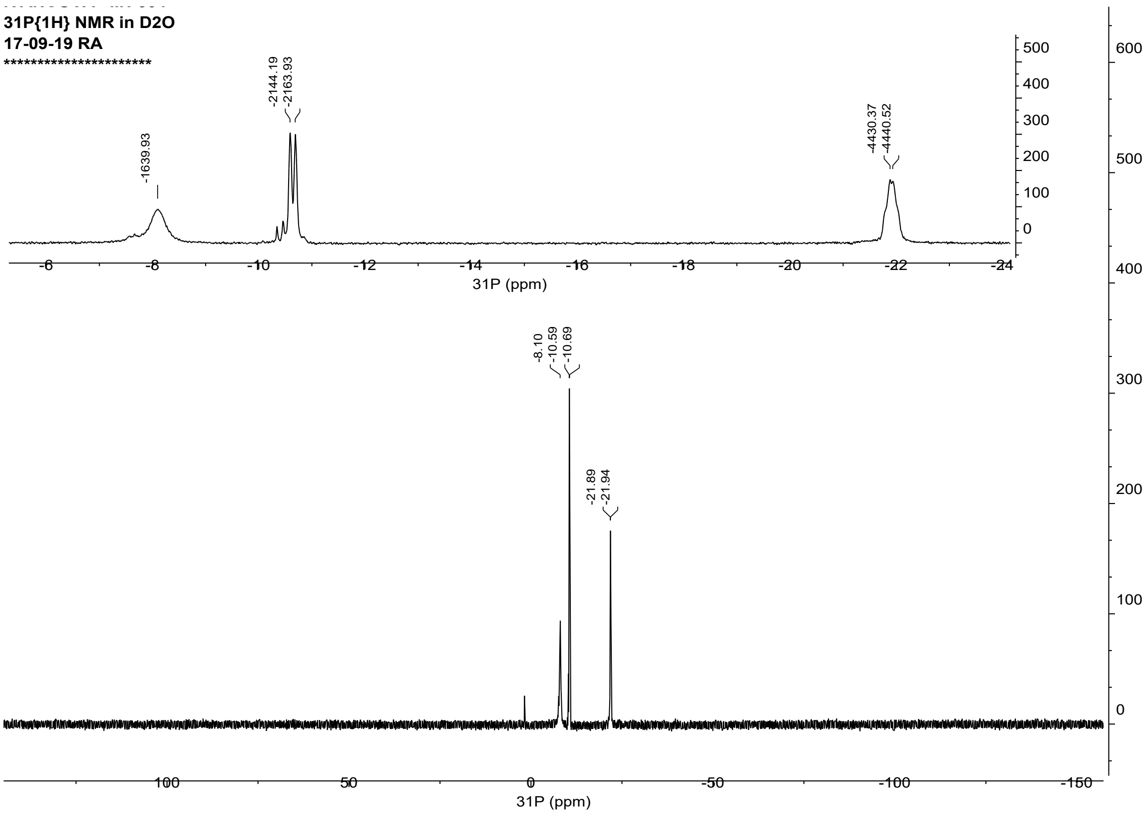

31P{1H} NMR in D2O
17-09-19 RA
****
-1639.93
-2144.19
-2163.93
-4430.37
-4440.52
31P (ppm)
-8.10
-10.59
-10.69
-21.89
-21.94
31P (ppm)

^{1}H, ^{13}C NMR spectra of PAS

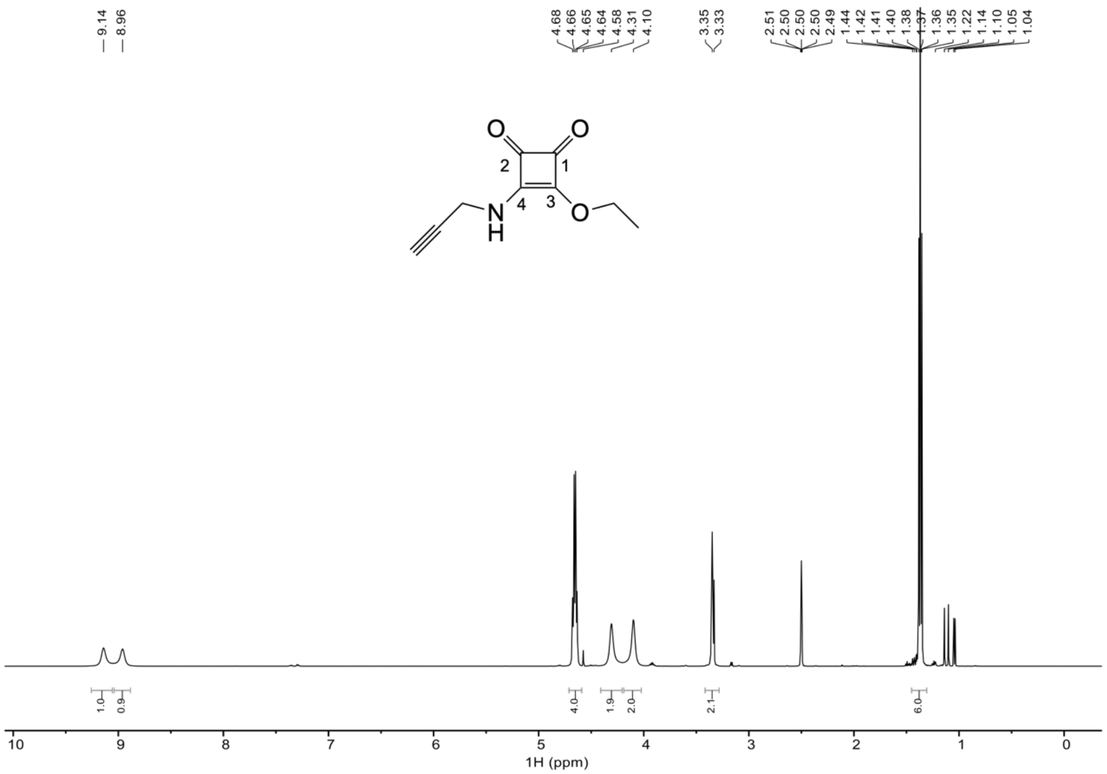

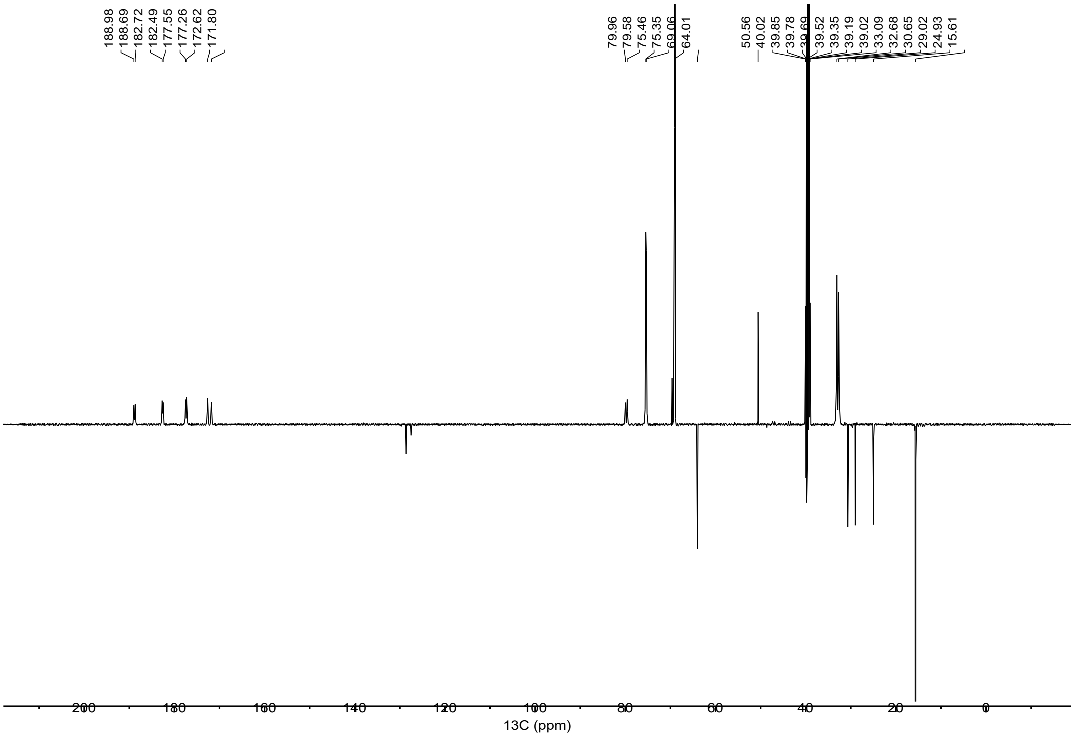

^{1}H, ^{13}C NMR spectra of A^{ESQ}

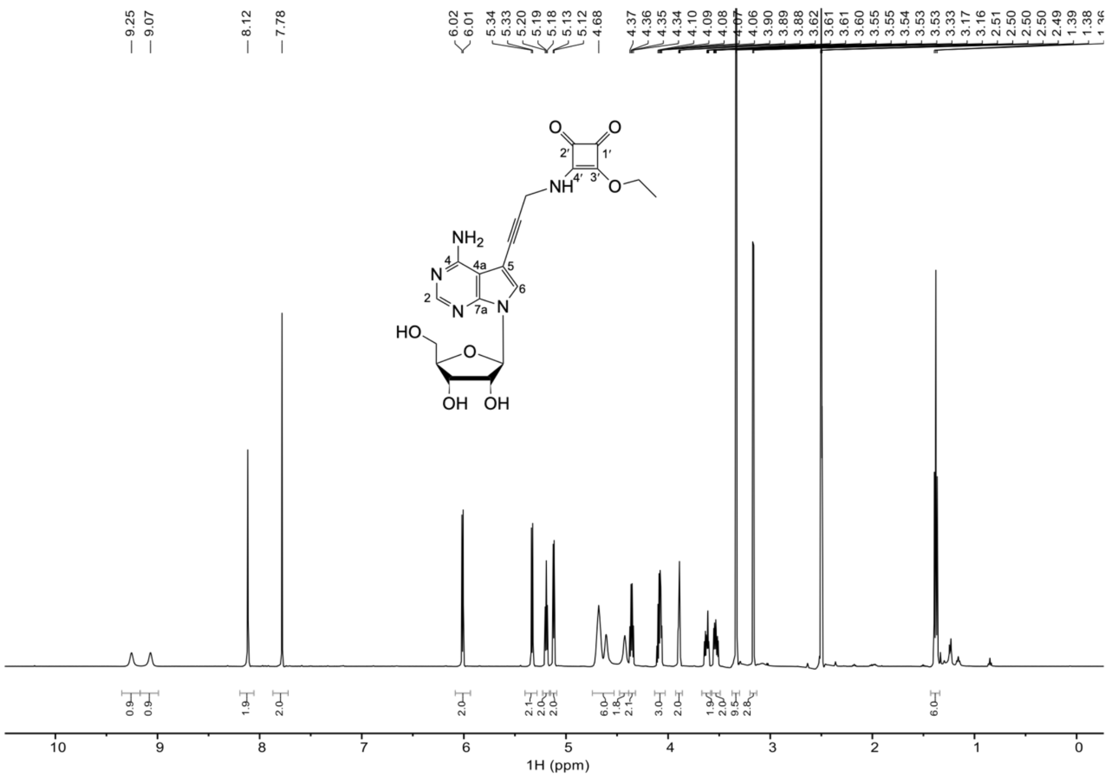

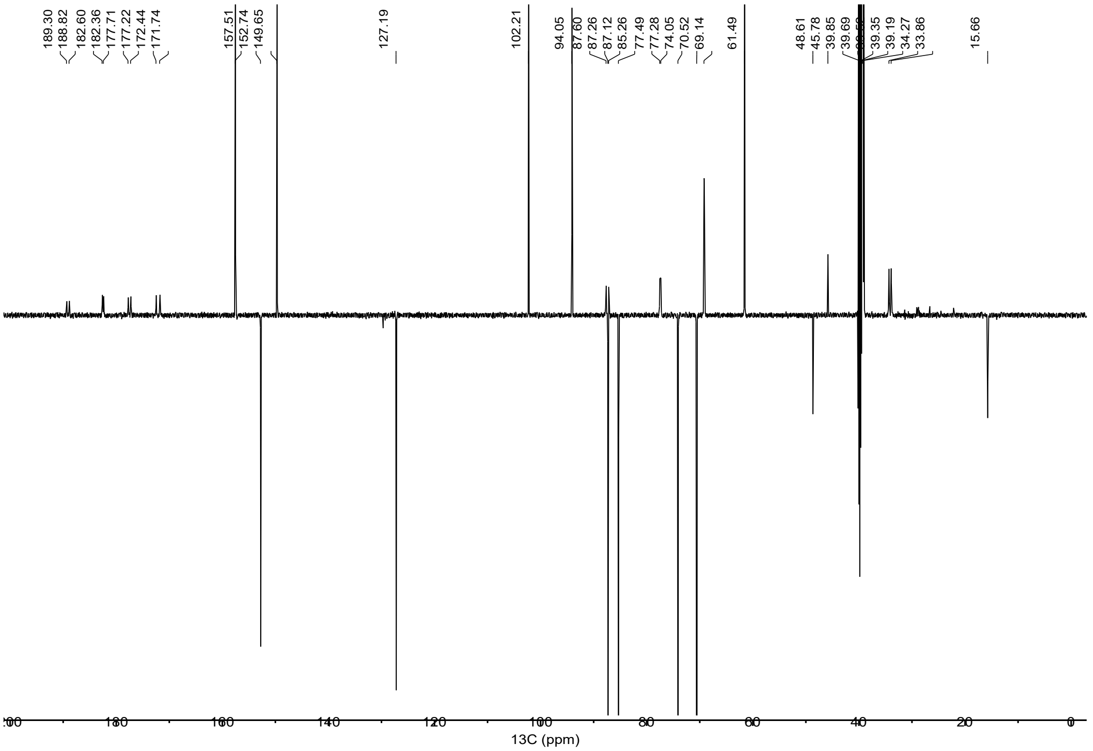

^{1}H, ^{13}C, ^{31}P NMR spectra of A^{ESQ}TP

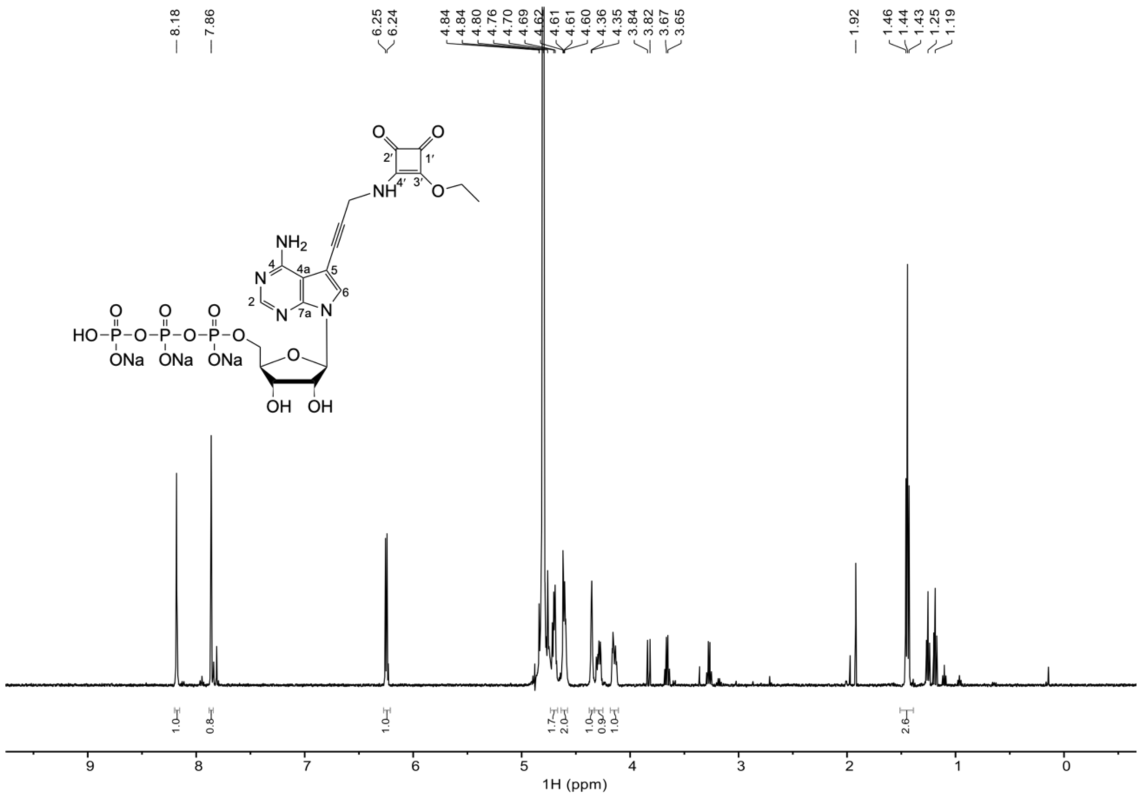

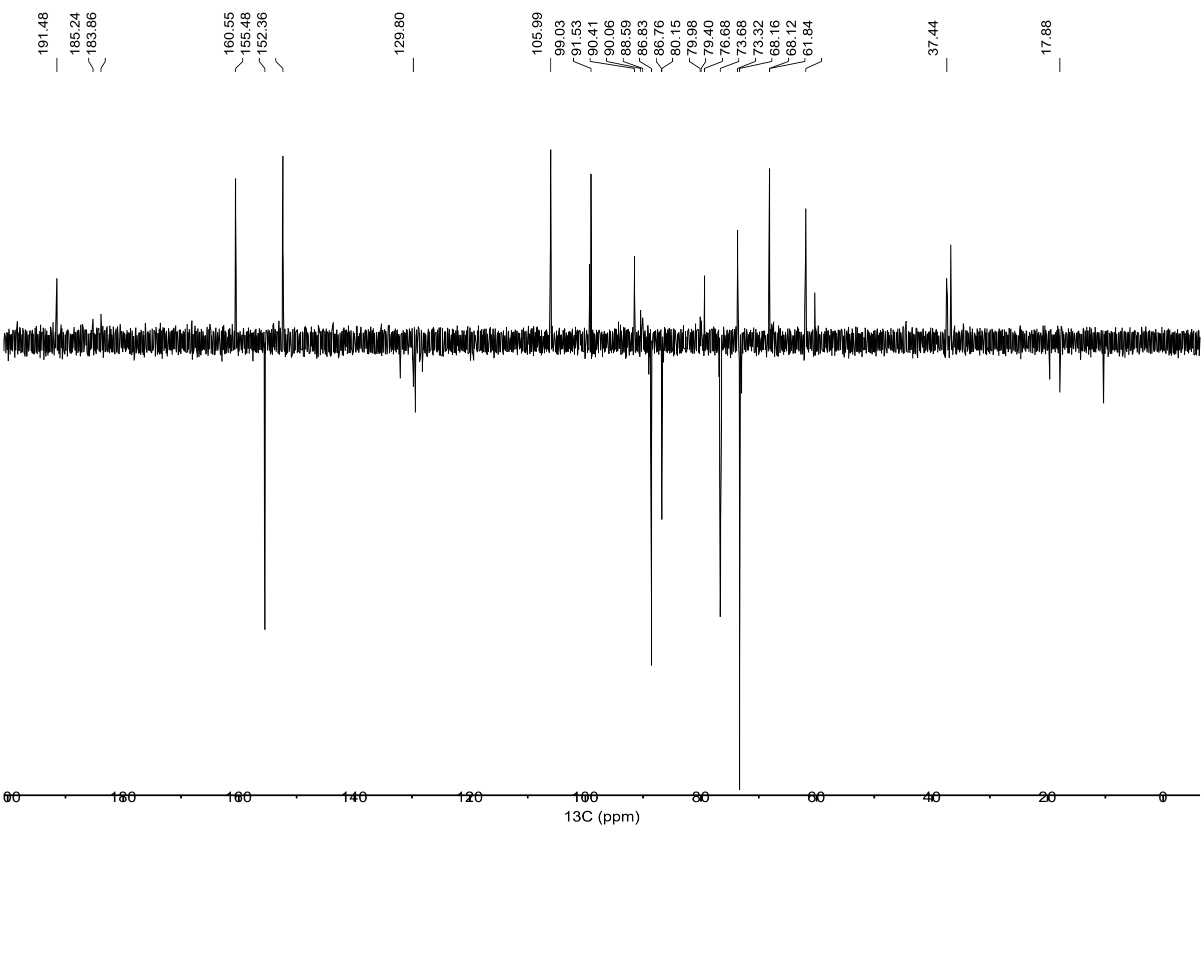

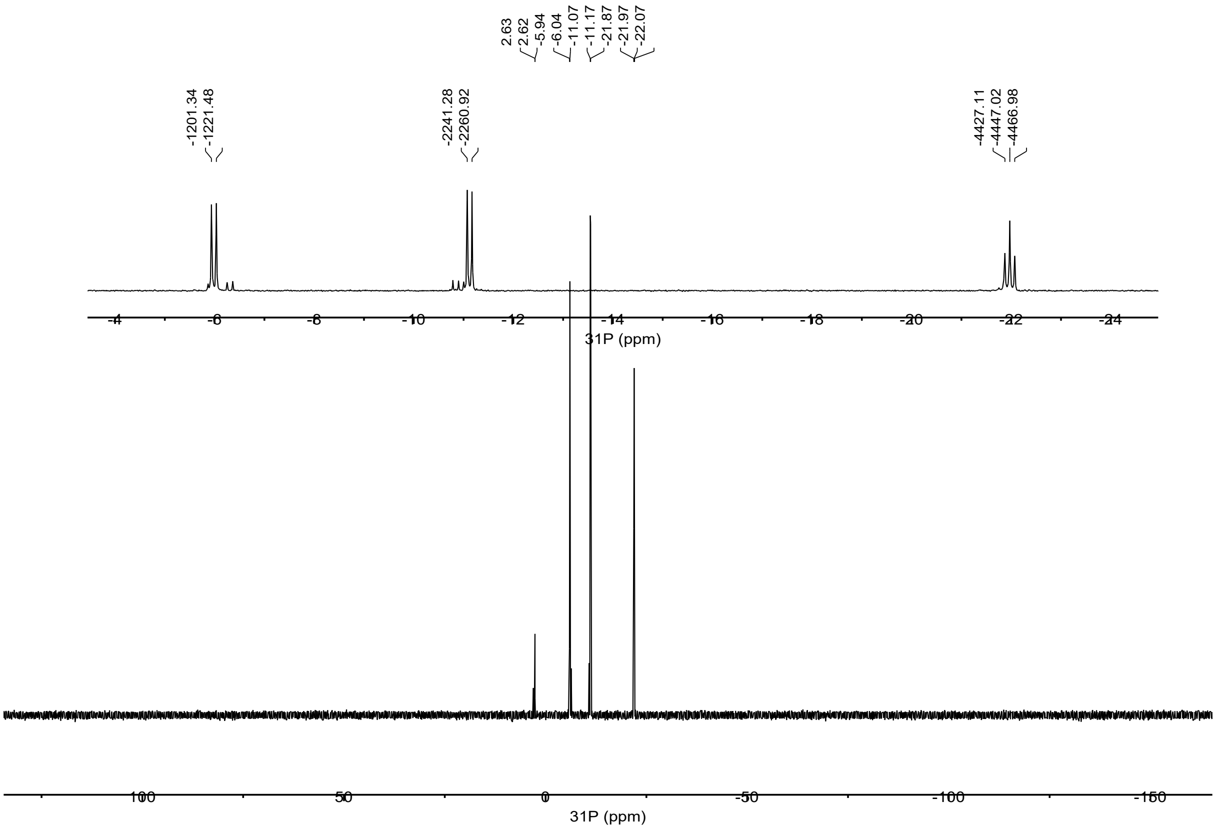
2.63
2.62
-5.94
-6.04
-11.07
-11.17
-21.87
-21.97
-22.07
-1201.34
-1221.48
-2241.28
-2260.92
-4427.11
-4447.02
-4466.98
-4
-6
-8
-10
-12
-14
-16
-18
-20
-22
-24
31P (ppm)
100
50
0
-50
-100
-150
31P (ppm)
