# Supplementary material for: Enzymatic synthesis of reactive RNA probes containing squaramate-linked cytidine or adenosine for bioconjugations and cross-linking with lysine-containing peptides and proteins
Source: Commun Chem. 2025 Jan 2;8:1. doi: 10.1038/s42004-024-01399-6 (PMC11696893; doi:10.1038/s42004-024-01399-6)
Supplement: Supplementary file 5 — Supplementary data 2 [file 42004_2024_1399_MOESM5_ESM.pdf]

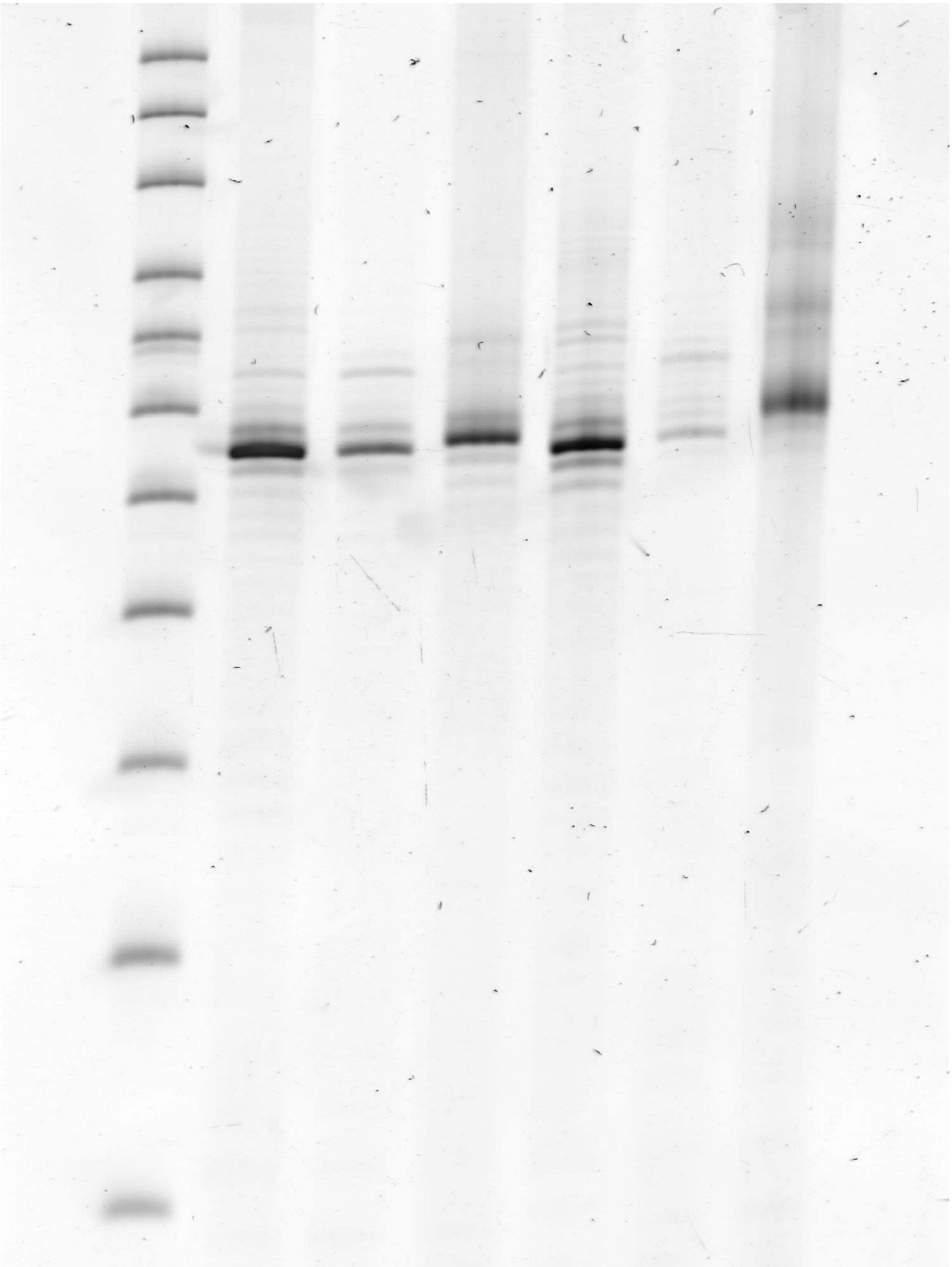

Uncropped gel from Figure 2C, upper gel.

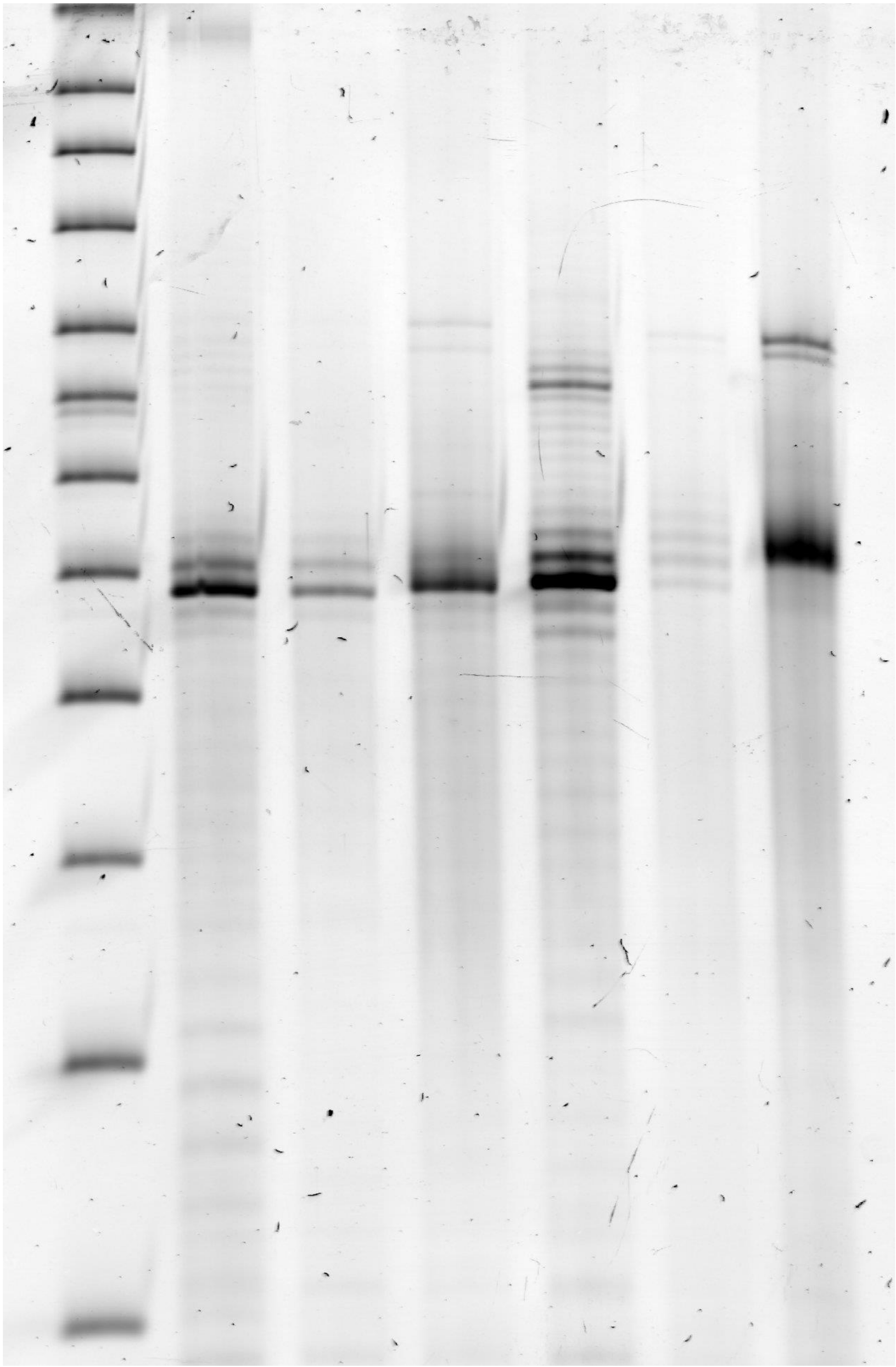

Uncropped gel from Figure 2C, lower gel.

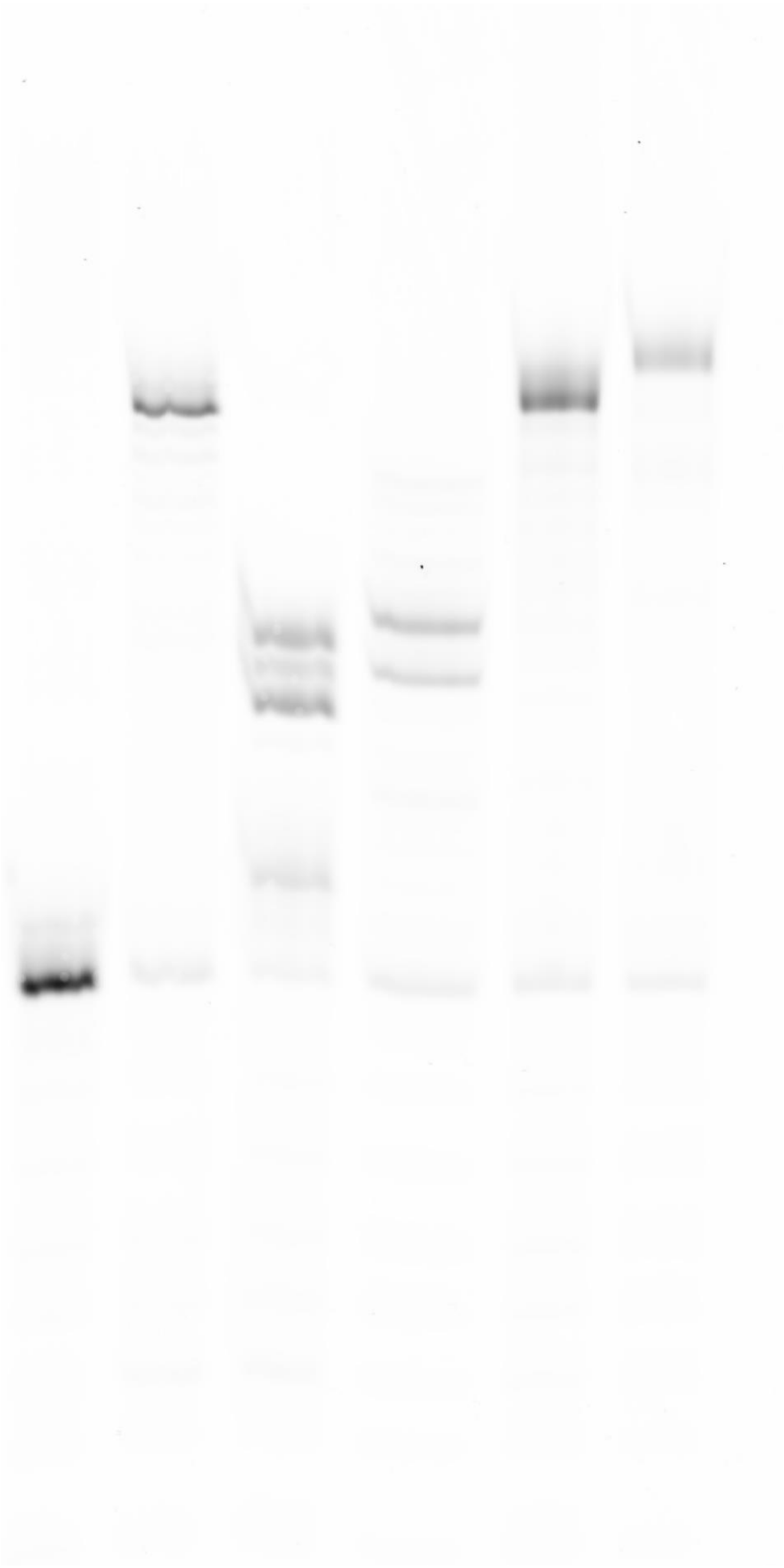

Uncropped gel from Figure 2E.

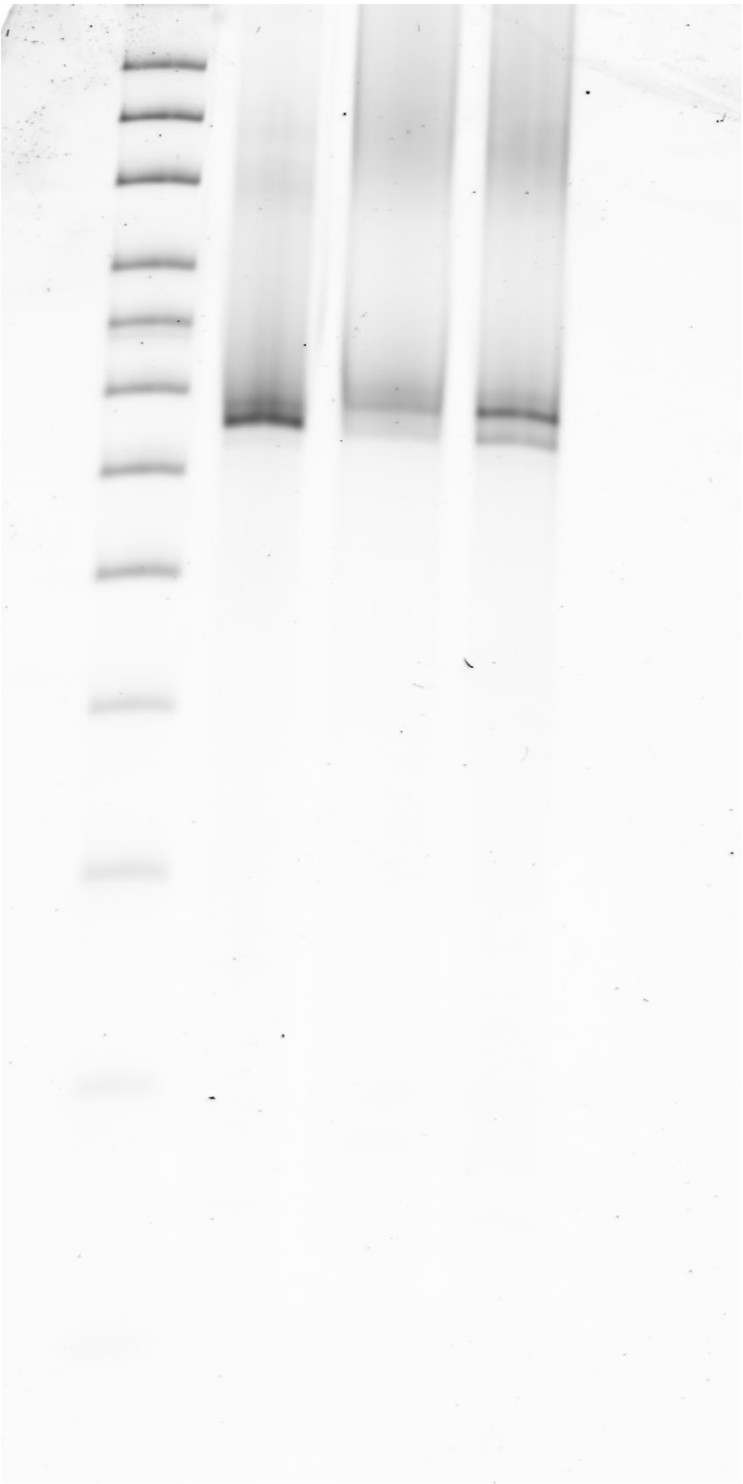

Uncropped gel from Figure 3D.

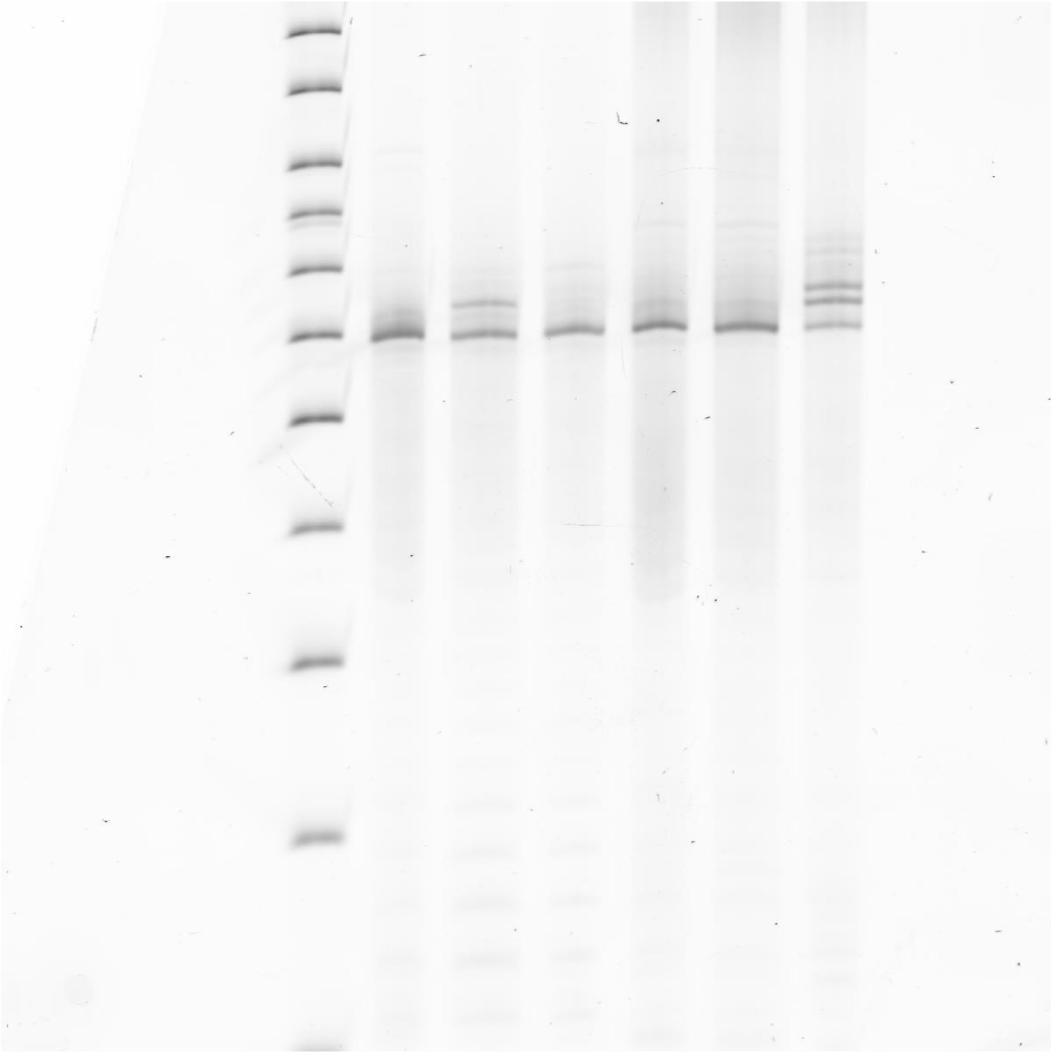

Uncropped gel from Figure 3F.

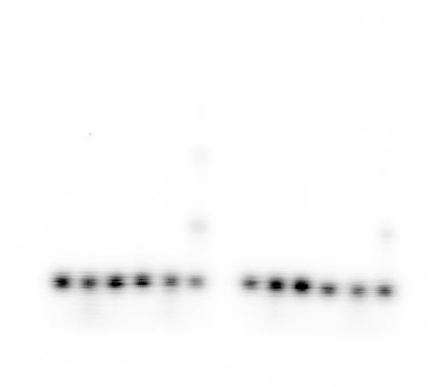

Uncropped gel from Figure 4B - 1st from left.

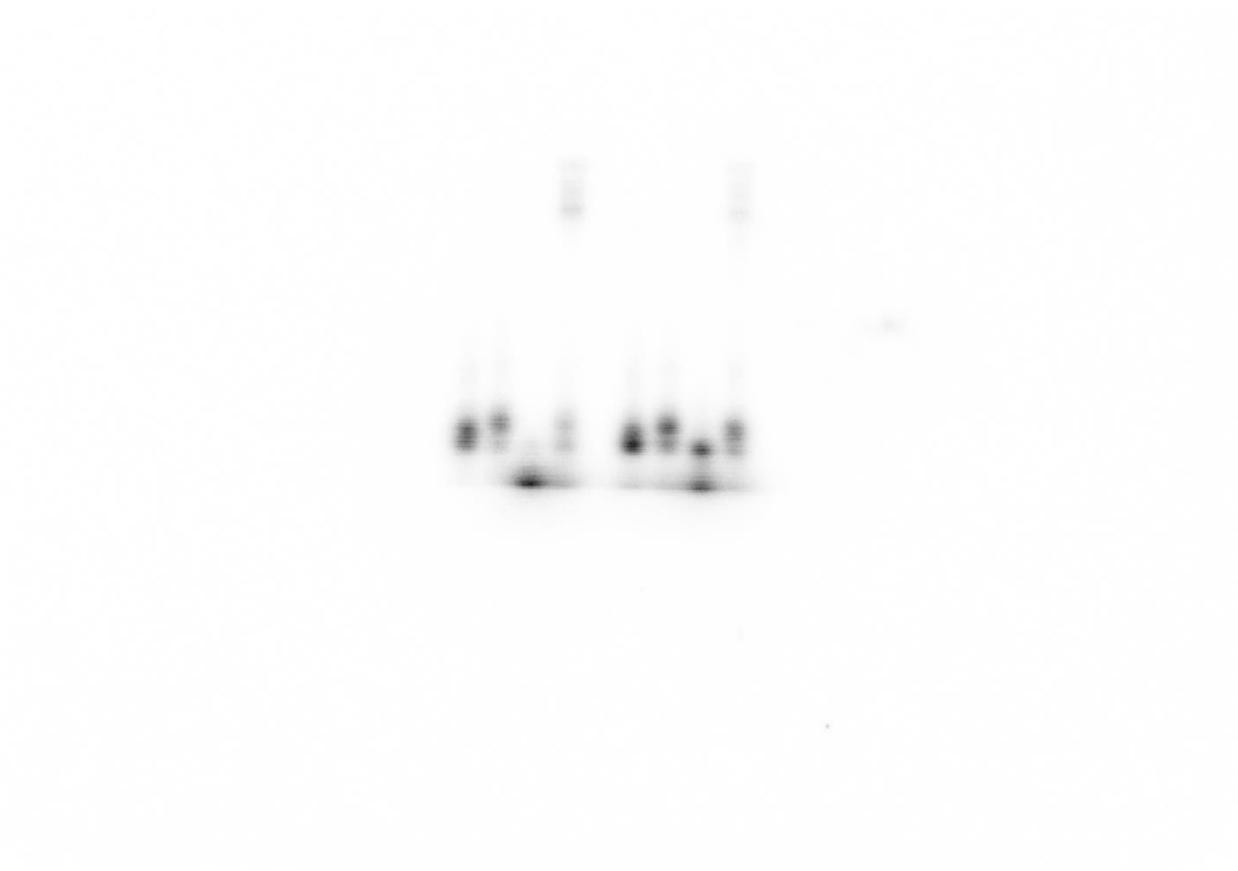

Uncropped gel from Figure 4B - 2nd from left.

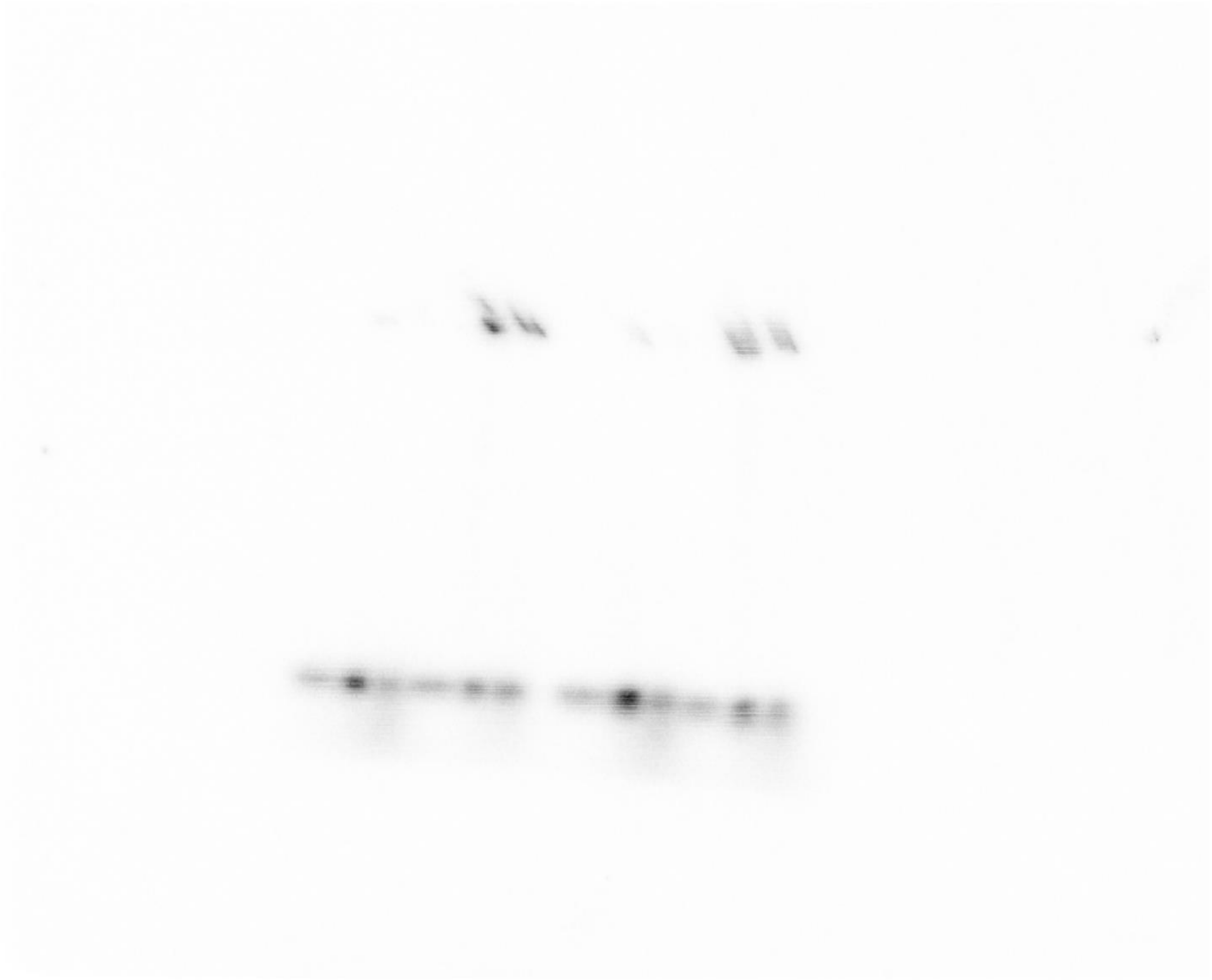

Uncropped gel from Figure 4B - 3rd from left.

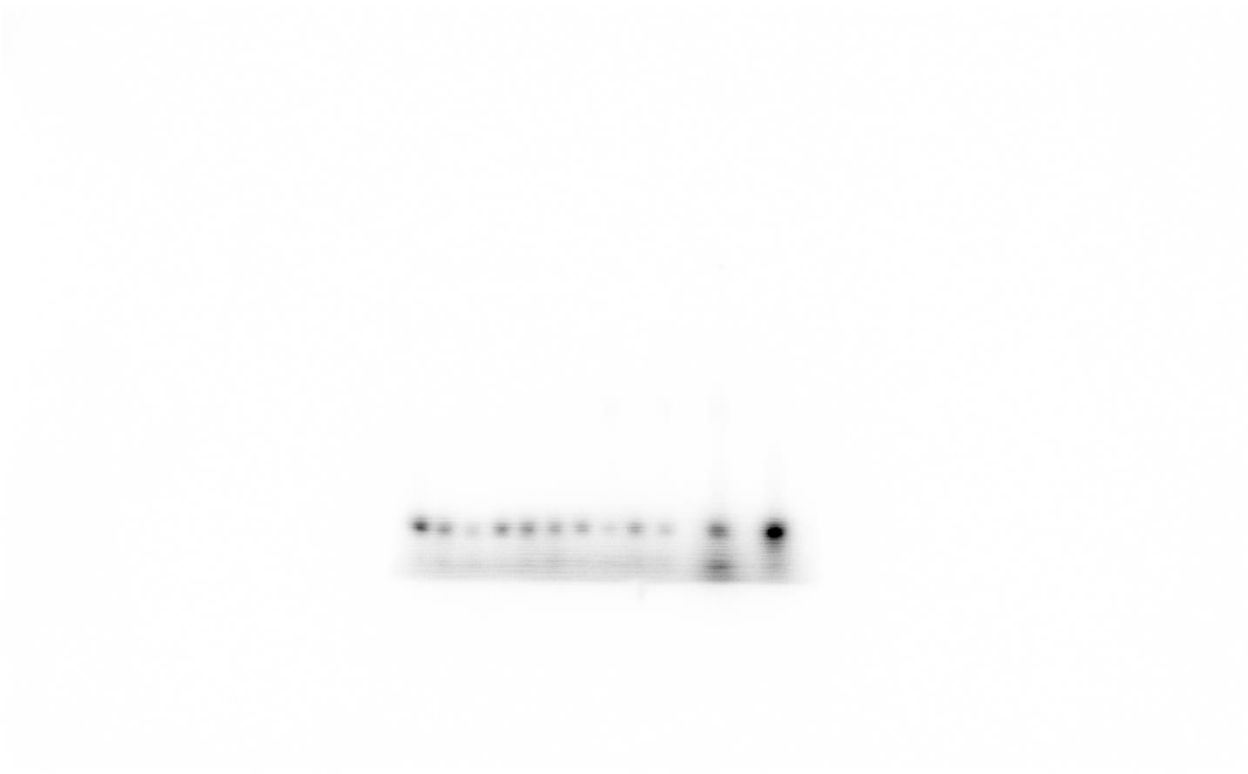

Uncropped gel from Figure 4B - 2nd from right.

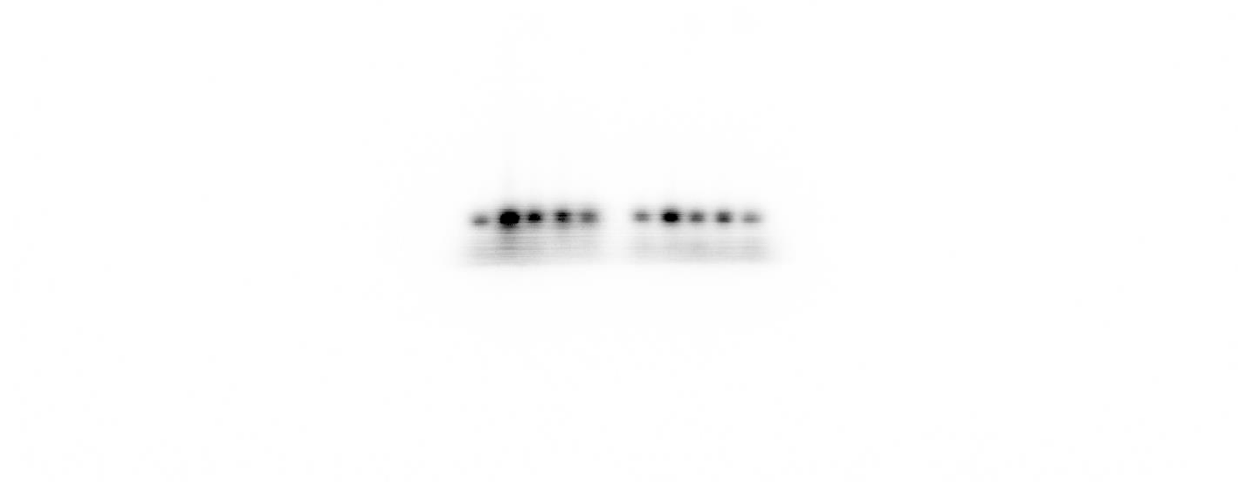

Uncropped gel from Figure 4B - 1st from right.

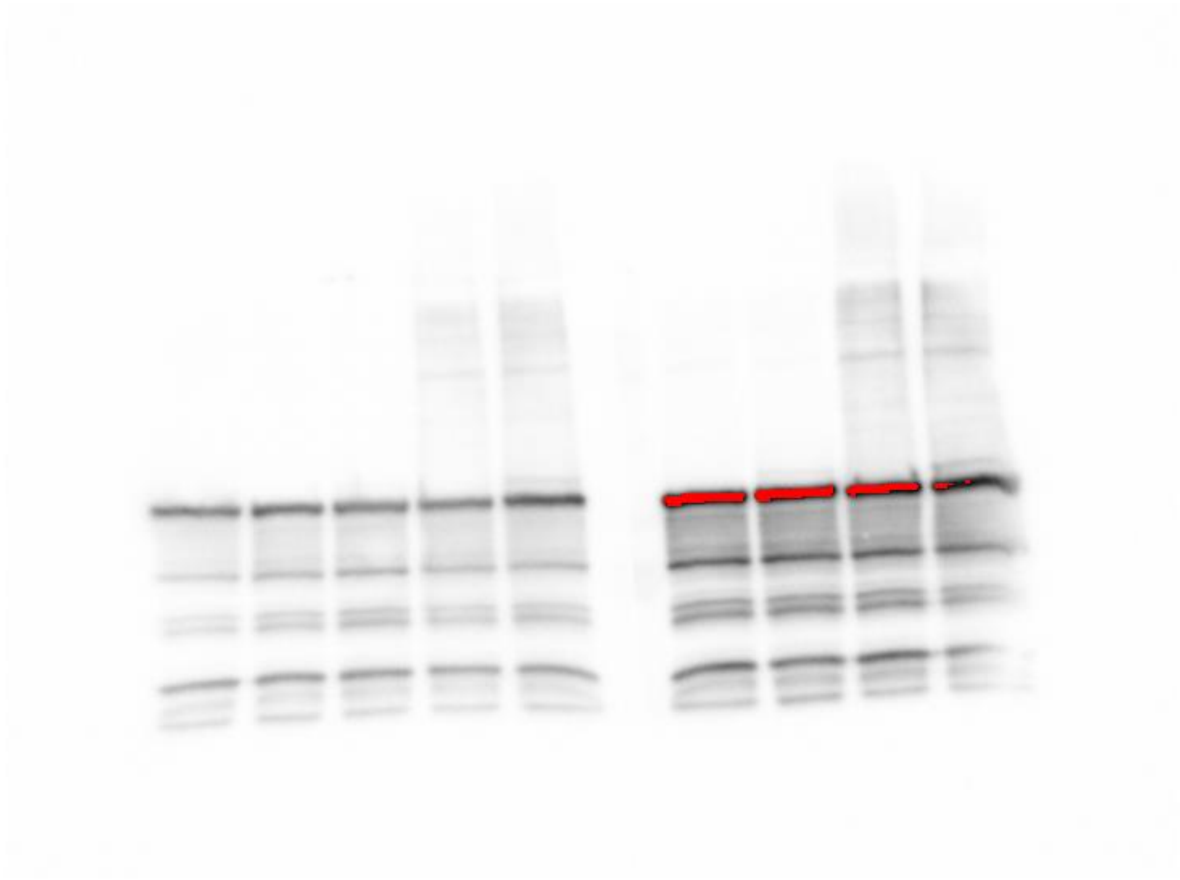

Uncropped gel from Figure 5B.

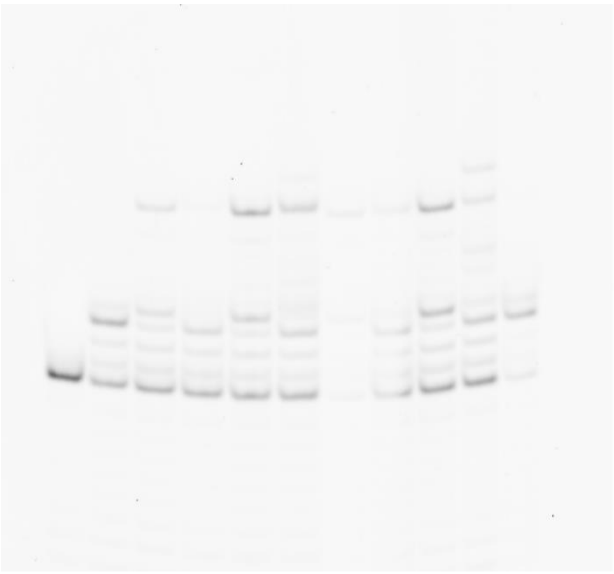

Uncropped gel from Figure 5C.

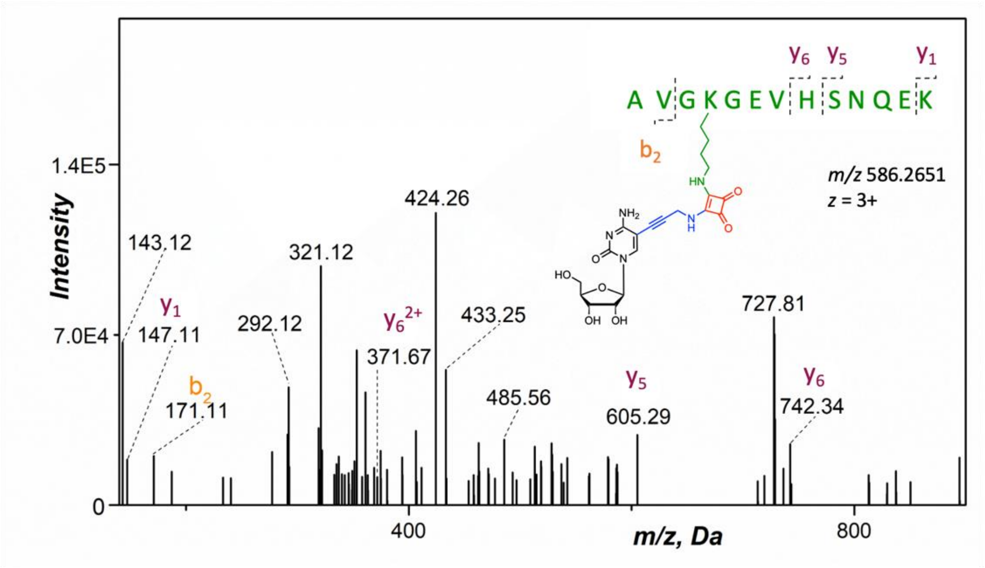

Uncropped gel from Figure 5D.
