# Supplementary material for: Enzymatic synthesis of reactive RNA probes containing squaramate-linked cytidine or adenosine for bioconjugations and cross-linking with lysine-containing peptides and proteins
Source: Commun Chem. 2025 Jan 2;8:1. doi: 10.1038/s42004-024-01399-6 (PMC11696893; doi:10.1038/s42004-024-01399-6)
Supplement: Supplementary file 1 — Transparent Peer Review file [file 42004_2024_1399_MOESM1_ESM.pdf]

This file contains all reviewer reports in order by version, followed by all author rebuttals in order by version.

nature portfolio

Peer Review File

Enzymatic Synthesis of Reactive RNA Probes Containing Squaramate-Linked Cytidine or Adenosine for Bioconjugations and Cross-Linking with Lysine-Containing Peptides and Proteins

Corresponding Author: Professor Michal Hocek

Version 0:

Reviewer comments:

Reviewer #1

(Remarks to the Author)
Michal Hocek and coauthors report the design, synthesis and enzymatic incorporation of a cytidine nucleotide bearing a reactive squaramate group that is compatible for cross linking with proteins and amine containing small molecule probes. They used T7 RNA polymerase and primer extension using an engineered TGK DNA polymerase that extends RNA primers. Hocek group recently used TGK DNA polymerase to elegantly incorporate modifications into RNA (Nature Communications 2024). The synthesized squaramate-modified RNA probes were cross-linked with RNA-binding proteins including viral RNA polymerases, HIV reverse-transcriptase, which proceeded with moderate to good conversions. Although the method described here has a couple of draw backs (kinetics is slow, requires high amounts of the reactive counterpart and squaramate can react with any primary amine present in the biomolecule), incorporating squaramate into RNAs is novel and its potential application in bioconjugation (RNA-protein cross linking) in vitro is useful and merits publication in this journal. The experiments have been designed and characterized well. I have some important corrections and suggestions.

Specific comments:
1. The authors state that “We used two 5'-terminal 2'-MeO ribonucleotides of antisense strand of DNA templates to minimize non-templated nucleotide addition.” In Figure S2, one could observe some amounts of non-template addition.

2. There seems to be differences in-between cropped image (Figure 2C) and uncropped gel image Figure S2. Further, in the - negative control in the absence of CTP, there is full length product formation, why? Please see Figure S2: 1C and 1eCESQ. Is there a misincorporation?
Labeling strategy used in these figures are different and hence confusing. What do you mean by “IVT with PEX reactions”.

3. Figure S3b. Reactions with spermidine, betaine and without any amines. The band intensity for the full-length product in (-) negative and (*) with modified nucleotide looks more or less same under all conditions. Why the authors claim that with spermidine there was reaction with the squaramate group? This section should be explained better. Further in the negative control also, the product is observed. Is there a misincorporation?

4. Line 194 and 204. Figure number should be changed.

5. Experimental design and results associated with Figure 5B could be explained better for the readers.

6. Why direct Sonogashira reaction with propargyl amine was not used for preparing 5-(3-aminopropyn-1-yl)-cytidine 3. Is there a reactivity problem?

Reviewer #2

(Remarks to the Author)
The authors have developed a cytidine 5′-O-triphosphate and a reactive squaramate group containing molecule (CESQTP) and incorporated inside the RNA using T7 RNA polymerase or primer extension using engineered TGK DNA polymerase. They used these probes to attach with RNA binding proteins covalently. These are also used to inhibit the activity of different RNA binding RNA polymerase from the Japanese Encephalitis virus. The authors have developed the probes and tried to prove their hypothesis using sufficient experimental evidence. However, similar kinds of works were also previously done by

the author, and hence, this reviewer found a lack of sufficient novelty in the present work. This reviewer recommends accepting the work only after providing sufficient justification and explanation for the novelty of the work compared to previously published DNA and RNA-based probes by the same group. Otherwise, this work can be rejected from the Communications Chemistry.
Comments to the authors:
1) Please give a justification about the novelty of the work as compared to the previously published work, e.g., Angew. Chem. Int. Ed. 62, e202213764 (2023), Angew. Chem. Int. Ed. 2019, 58, 13345 –13348.
2) Please provide a graphical representation or a cartoon image summarizing the chemistries, and the types of the probes discussed in the introduction.
3) Line 60, …. Binding proteins. Please provide proper references in support of the comment.
4) Line 98, eith, correct the spelling.
5) Line 115, …. Was less efficient (Fig.2C) why???? Please elaborate.
6) Line 163, mixture of RNA-peptide conjugates….What is the reason for observing multiple bands? Is it because the reaction is not that efficient that within the stipulated time, it is not completed?
7) Line 192, .. Fig. 2b. Correct the numbering of the figure.
8) What is the driving force for the reaction of the probes and the lysine residues of the proteins? Why are some RNA binding proteins with less Lys residue showing a higher conversion rate? Please comment on the product yield in Table 1.
9)Use a high resolution image for mass spec in 5d.
10) What are those unintegrated peaks in 1H, 13C NMR spectra of CPA, Page S10. Please pick the 13C peaks on page S10.
11) In the 1H NMR of CESQ, Page S11, the peaks on 8.95 and 9.11 are mentioned by the author as 2 protons but integrated as 1 proton. Please check the spectra.
12) Please use a high resolution image for the mass spec in the supporting materials as well. (Figure S3).

Reviewer #3

(Remarks to the Author)
The authors have developed a bioconjugation system for RNA labelling and RNA-protein crosslinking using squaramate-linked cytidine. This is a useful approach for RNA labelling and RNA-protein crosslinking though the basic methods of this study have been previously reported by the same group. (Angew. Chem. Int. Ed. 62, e202213764 (2023) and Angew. Chem. Int. Ed. 58, 13345–13348 (2019).). The work is well-conducted and described, however this reviewer has concerns regarding the similarity to the previous study. In 2023 Angew. Chem. Int. Ed., the authors used A analog for RNA conjugations and cross-linking using chloroacetamide-modified nucleotide. In present study, C analog was used like DNA case in 2019 Angew. Chem. Int. Ed..
Major
1: As a follow-up to similar studies, it would be better to describe the base generality. At least, this reviewer would like to know whether this squaramate-linked chemistry is applicable to A analog or not.
2: Why did the authors select cytidine analog for squaramate-linkage? It would be better to describe some more molecular design and strategy.
3: If possible, it would be interesting to see differences in RNA-protein cross-linking between squaramate- and chloroacetamide-modified RNAs.
4: In conclusion, it would be better to briefly compare the present results with previous studies and discuss the advantages of this study. Probably, amine reactivity will be advantage compared to chloroacetamide-modified nucleotide.
Minor
4: Figure 1 (right chemical structure): I think N should be NH.
5: P. 5 line 98: …guided the incorporation of one to eith of CESQ nucleotide into RNA transcript.
I think eith should be eight
6: P. 7 line 139: Model cross-linking reaction…
I think conjugation (or labelling) reaction is better.
7: P. 9 line 178: Single → single
8: P. 11 Second Figure 2 should be Figure 4.
9: P. 12 Please check Fig numbers.

Version 1:

Reviewer comments:

Reviewer #1

(Remarks to the Author)
The authors have performed additional experiments and revised the manuscript according to the comments and suggestions. I am satisfied with the revised version of the manuscript and I recommend publication in this journal.

Reviewer #2

(Remarks to the Author)
The authors have responded all the queries and comments raised by this reviewer. This reviewer is satisfied with the responses, additions of the figures and rectification.

The manuscript can be accepted in its present form.

Reviewer #3

(Remarks to the Author)
The authors have addressed satisfactorily all my comments, and I consider the paper ready for publication.

Open Access This Peer Review File is licensed under a Creative Commons Attribution 4.0 International License, which permits use, sharing, adaptation, distribution and reproduction in any medium or format, as long as you give appropriate credit to the original author(s) and the source, provide a link to the Creative Commons license, and indicate if changes were made.
In cases where reviewers are anonymous, credit should be given to 'Anonymous Referee' and the source.
The images or other third party material in this Peer Review File are included in the article's Creative Commons license, unless indicated otherwise in a credit line to the material. If material is not included in the article's Creative Commons license and your intended use is not permitted by statutory regulation or exceeds the permitted use, you will need to obtain permission directly from the copyright holder.
To view a copy of this license, visit https://creativecommons.org/licenses/by/4.0/

Responses to the reviewers and list of changes

General remarks to the reviewers:

Please note that we have added the synthesis of **A^{ESQ}TP** and we used it for IVT, PEX and cross-linking reactions. Also, we added direct comparison of the specificity of the reactions with the previously reported chloroacetamide RNA. Since the first author has already left my group for postdoc, all these additional experiments were performed by another student (Tania Sanchez, who has now shared first authorship). The new IVT and cross-linking experiments were not performed using radioactive labelling but using post-staining with Sybr-Gold – so the new gels look somewhat different than the original radioactive gels (some of which we still keep in the SI) but the results are consistent and reproducible.

Reviewer #1 (Remarks to the Author):

1. The authors state that "We used two 5'-terminal 2'-MeO ribonucleotides of antisense strand of DNA templates to minimize non-templated nucleotide addition." In Figure S2, one could observe some amounts of non-template addition.

Response: We agree that in the Figure S2 (now S3), there are still some amounts of the extended products despite the use of the two 5'-terminal 2'-MeO ribonucleotides in the antisense strand of DNA templates were reported to reduce the non-templated additions.
Revision: we have re-made the IVT under optimized conditions with **C^{ESQ}TP** and also with new **A^{ESQ}TP** and used staining with Sybr-Gold and we observed much less non-templated extended products (see Figure 2C). We have also exchanged reference 47 for a more relevant paper.

2. There seems to be differences in-between cropped image (Figure 2C) and uncropped gel image Figure S2.

Response: Thank you for your observation.
Revision: We have completely remade the Figure 2 with newly made gels.

Further, in the - negative control in the absence of CTP, there is full length product formation, why? Please see Figure S2: 1C and 1eCESQ. Is there a misincorporation?

Response: Negative controls for 1 incorporation very often provide similar results as positive controls due to misincorporation. We have proved by MALDI-TOF that under the conditions of negative controls, UTP misincorporates. See e.g.: Pomerantz, R. T. et al Mechanism of Nucleotide Misincorporation during Transcription due to Template-Strand Misalignment. *Molecular Cell* **24**, 245-255, (2006). Please note that we have proved the identity of the modified RNAs using MALDI or LC-MS and also the presence of the modification was proved by the follow-up reactions.
Revision: We have revised and extended the discussion of this:
The IVT synthesis of RNA containing one or three **C^{ESQ}** or **A^{ESQ}** modifications (i.e. **35_RNA_1C^{ESQ}** or **35_RNA_3C^{ESQ}**) proceeded smoothly with good conversions (**Fig. 2C**), whereas the formation of RNA with higher number of reactive modifications (i.e. **35_RNA_7C^{ESQ}**) was less efficient (**Supplementary Fig. S3,4**). In some cases, we have observed formation of minor amounts of n+1 products of non-templated extension of the RNA products (**Fig. 2C** and **Supplementary Fig. S3-6**). In case of IVT with templates encoding for incorporation of just one modified nucleotide, the negative control experiments in the absence of the corresponding non-modified NTP also showed some formation of full-lengh product due to misincorporation of uridine, which is quite common in such cases.[49] In all cases, the identity of the full-length modified RNA products were also confirmed by MALDI-TOF (**Supplementary Fig. S22-27**) or LC-MS (**Supplementary Fig. S33-34, S37-38**).

Labeling strategy used in these figures are different and hence confusing.

Response: Thank you for your comment.
Revision: We have revised the Figures and made the labels clear and consistent.

What do you mean by "IVT with PEX reactions".

Response: thank you for spotting this mistake
Revision: it was corrected.

3. Figure S3b. Reactions with spermidine, betaine and without any amines. The band intensity for the full-length product in (-) negative and (*) with modified nucleotide looks more or less same under all conditions. Why the authors claim that with spermidine there was reaction with the squaramate group? This section should be explained better.

Response: We can clearly see a band with slower electrophoretic mobility in the * lane of condition A (commercial buffer with spermidine). Spermidine is a primary amine and thus easily reacts with ESQ moiety. We have proved this situation repeatedly within revisions – Figure S7 and confirmed the spermidine coupled to the ESQ-modified RNA by LC-MS. Under conditions with either quarternary amines (betaine) or with no amine, there is no additional band and only the modified RNA is formed.

Revision: we have revised and clarified the paragraph:
Since commercial transcription buffer supplied with the T7 RNAP contains spermidine and the squaramate group is known to readily react with primary amines,[48] we had to optimize the IVT conditions and composition of the buffers to prevent an undesired reaction of the ESQ-linked nucleotide or RNA with spermidine. Hence the spermidine was either replaced with quaternary amine betaine or we used a buffer with no amines (**Supplementary Fig. S4**, **S7**, **S21**, and **S35**).

Further in the negative control also, the product is observed. Is there a misincorporation?

Response: Here we refer to our previous answer and confirm the misincorporation of UTP.

4. Line 194 and 204. Figure number should be changed.

Revision: we corrected this

5. Experimental design and results associated with Figure 5B could be explained better for the readers.

Response: we agree that the design was suboptimal and somewhat confusing.
Revision: We have redesigned and clarified the figure .

6. Why direct Sonogashira reaction with propargyl amine was not used for preparing 5-(3-aminopropyn-1-yl)-cytidine 3. Is there a reactivity problem?
Response: We followed a previously established procedure for the synthesis of 5-(3-aminopropyn-1-yl)-cytidine (**3**) also cited in the Supplementary Information (SI). The direct Sonogashira reaction with propargyl amine could be problematic due to the free amine group, which can coordinate with the palladium catalyst or undergo side reactions, reducing the reaction's efficiency. Since it is a known synthesis of a known compound, we did not want to discuss this issue.

Reviewer #2 (Remarks to the Author):

1) Please give a justification about the novelty of the work as compared to the previously published work, e.g., Angew. Chem. Int. Ed. 62, e202213764 (2023), Angew. Chem. Int. Ed. 2019, 58, 13345 – 13348.

Response: we gave clear justification of the work in this sentence:

Recently, we reported chloroacetamide (CA)-linked ATP that can be incorporated into RNA by *in vitro* transcription (IVT) using T7 RNA polymerase (T7 RNAP) and cross-links to Cys- or His-containing peptides and proteins,[34] but there is an urgent need for reactive RNA probes specific for Lys as one of the most abundant amino acid in nucleic acids binding proteins.[35-37]

Previously we have reported the squaramate modification for synthesis of DNA probes (Angew. Chem. Int. Ed. 2019, 58, 13345 –13348) and in another paper we used chloroacetamide modification for RNA (Angew. Chem. Int. Ed. 62, e202213764 (2023)). The previously reported RNA modification is specific for Cys and partially also for His which are very rare in RNA-binding proteins. There is a high need for an RNA modification specific for Lys which is the most abundant amino acid in NA-binding proteins.

Revision: we have added references for the statement that Lys is the most abundant amino acid in nucleic acids-binding proteins.

Also based on the second request of the Reviewer 2, we have extended Figure 1 with the related previous results.

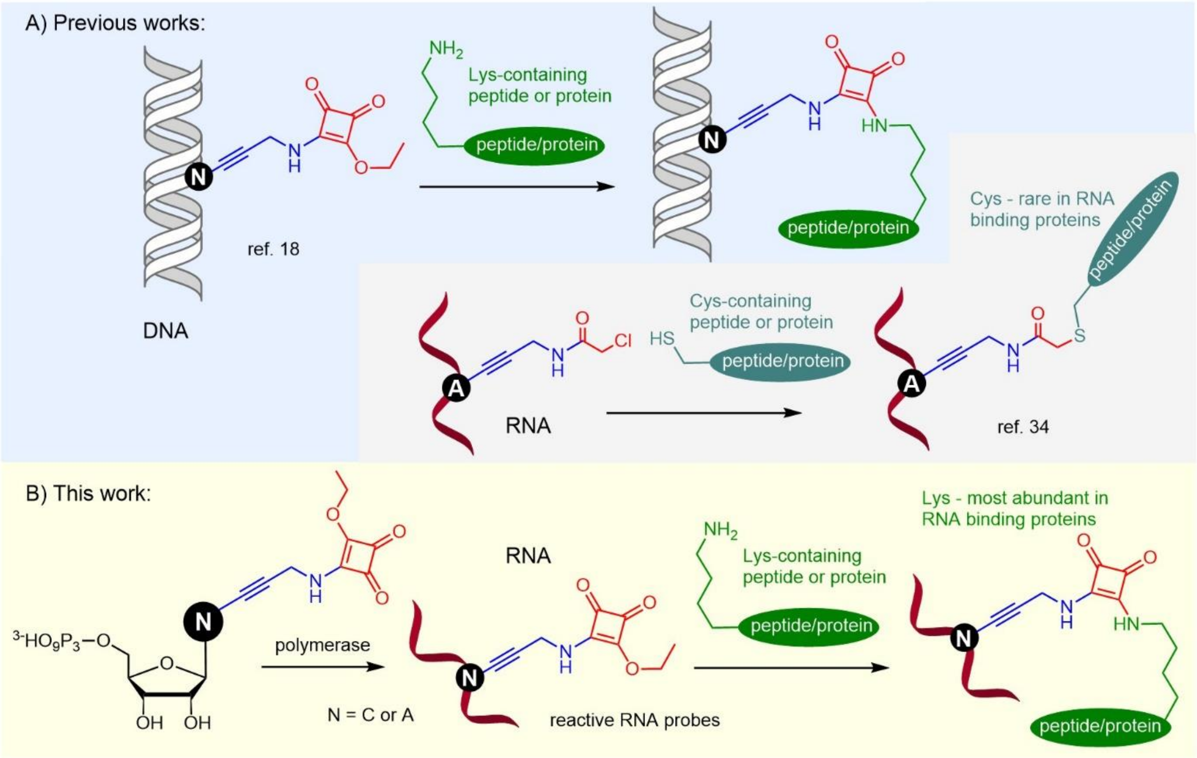

2) Please provide a graphical representation or a cartoon image summarizing the chemistries, and the types of the probes discussed in the introduction.

Revision: extended Figure 1 added – see above.

3) Line 60, …. Binding proteins. Please provide proper references in support of the comment.

Response: We thank you for your comment.

Revision: We have attached three new references.

Bartas, M., Červeň, J., Guziurová, S., Slychko, K. & Pečinka, P. Amino acid composition in various types of nucleic acid-binding proteins. *Int. J. Mol. Sci.* **22**, 922 (2021).

Hacker, S. et al. Global profiling of lysine reactivity and ligandability in the human proteome. *Nature Chem* **9**, 1181–1190 (2017).

Ukmar-Godec, T., Hutten, S., Grieshop, M.P. *et al.* Lysine/RNA-interactions drive and regulate biomolecular condensation. *Nat Commun* **10**, 2909 (2019).

4) Line 98, eith, correct the spelling.

Revision: corrected

5) Line 115, …. Was less efficient (Fig.2C) why???? Please elaborate.

Response: it is quite common and obvious that the enzymatic synthesis of RNA containing higher number of modifications is more difficult and less efficient – especially in the case of reactive modifications. Since we have not studied that in deeper detail and for most of the useful RNA probes one modification is sufficient, we would rather not divert the flow of the discussion into some speculations why the RNA with high density of reactive modifications was formed in lower yield.
Revision: the whole paragraph has been revised based on the other comments of all reviewers.

6) Line 163, mixture of RNA-peptide conjugates….What is the reason for observing multiple bands? Is it because the reaction is not that efficient that within the stipulated time, it is not completed?

Response: With increased excess of the peptide in reaction with **35_RNA_3C^{ESQ}**, we observed higher conversion to the fully (triply) modified conjugate but we never reached the full conversion (there were always some doubly-modified products present). Please note that the tripeptide does not have any special affinity to the RNA and there is no proximity effect here.
Revision: we have slightly modified and clarified the discussion.

7) Line 192, .. Fig. 2b. Correct the numbering of the figure.

Revision: we completely redesigned Figure 2

8) What is the driving force for the reaction of the probes and the lysine residues of the proteins? Why are some RNA binding proteins with less Lys residue showing a higher conversion rate? Please comment on the product yield in Table 1.

Response: the cross-linking reaction needs a proximity effect and so the outcome of the reaction depends not only on the number of Lys but also on their position in the RNA-recognition site and proximity to the reactive group. Since we do not have any structural information, we would rather not speculate too much here.

Revision; we added a sentence:
It should be noted that proximity effect is needed for an efficient cross-linking reaction and hence it is not the number of lysines but the accessibility and optimum positioning of one or more lysines in the vicinity to the reactive group is the driving force and decisive factor for the outcome of these reactions.

9)Use a high resolution image for mass spec in 5d.

Revision: we replaced the figure

10) What are those unintegrated peaks in 1H, 13C NMR spectra of C^{PA}, Page S10. Please pick the 13C peaks on page S10.

Response: The unintegrated peaks in the NMR spectra of C^{PA} molecules were identified as impurities from the reaction. Since it was just a known intermediate, the student did not repurify the bulk material and used it for the next steps.

Revision: We have replaced the 1H-NMR spectrum for the spectrum of pure sample and all the peaks are now integrated. We do not have the 13C NMR of this pure sample but since it is a known compound, it is not needed for the characterization.

11) In the 1H NMR of CESQ, Page S11, the peaks on 8.95 and 9.11 are mentioned by the author as 2 protons but integrated as 1 proton. Please check the spectra.

Response: We thank you for your remark.
Revision: We have fixed the wrong integrations.

12) Please use a high resolution image for the mass spec in the supporting materials as well. (Figure S3).

Revision: We have replaced the figure with the one with a higher resolution. After revisions, initial Figure S3 became S4.

Reviewer #3 (Remarks to the Author):

1: As a follow-up to similar studies, it would be better to describe the base generality. At least, this reviewer would like to know whether this squaramate-linked chemistry is applicable to A analog or not.

Response: we have originally synthesized only the CTP analog because we have previously observed that the modifications work the same way on every nucleobase. However, to prove that we have now synthesized also the A^{ESQ}TP and performed the IVT and PEX with it as well as the reactions with peptides.

Revision: we added the synthesis of A^{ESQ}TP and the IVT and PEX with it as well as the reactions with peptides. Figures 2 and 3 have been extended with new data.

2: Why did the authors select cytidine analog for squaramate-linkage? It would be better to describe some more molecular design and strategy.

Response: we selected cytidine because we have previously used adenosine for the complementary chloroacetamide modification. But based on the previous request, we have now added the **A^{ESQ}TP** as well.

3: If possible, it would be interesting to see differences in RNA-protein cross-linking between squaramate- and chloroacetamide-modified RNAs.

Revision: We have performed the experiments as asked – see Figure 3E,F. The dPAGE analysis showed specificity of each particular probe to either Cys or Lys residues.

4: In conclusion, it would be better to briefly compare the present results with previous studies and discuss the advantages of this study. Probably, amine reactivity will be advantage compared to chloroacetamide-modified nucleotide.

Revision: We added these two sentences to the Conclusions:
Squaramate moiety in RNA probes showed its specificity for cross-linking reactions with lysine-containing peptides and no reactivity with cysteine-containing peptides and thus is fully complementary to the previously reported[34] chloroacetamide (CA) modification that reacts with Cys (and His) but not Lys.

It is complementary to the previously reported chloroacetamide reactive group[34] and together or even in combination they can be used for RNA proteomics to identify new RNA-binding proteins.

4: Figure 1 (right chemical structure): I think N should be NH.

Thank you for your remark.
Revision: We have fixed the structure.

5: P. 5 line 98: …guided the incorporation of one to eith of CESQ nucleotide into RNA transcript. I think eith should be eight

Revision: We have fixed the mistake.

6: P. 7 line 139: Model cross-linking reaction…

Revision: We rewrote the original sentence "model cross-linking reaction" with "model conjugation reaction"

7: P. 9 line 178: Single → single

Revision: We have fixed it.

8: P. 11 Second Figure 2 should be Figure 4.

Revision: We have fixed the numbering.

9: P. 12 Please check Fig numbers.

Revision: All figures were renumbered in revision.
